# Supplementary material for: A cybergenetic framework for engineering intein-mediated integral feedback control systems
Source: Nat Commun. 2023 Mar 11;14:1337. doi: 10.1038/s41467-023-36863-9 (PMC10008564; doi:10.1038/s41467-023-36863-9)
Supplement: Supplementary file 1 — Supplementary Information [file 41467_2023_36863_MOESM1_ESM.pdf]

Supplementary Information
A cybergenetic framework for engineering intein-mediated integral feedback control systems

Stanislav Anastassov[a,1], Maurice Filo[a,1], Ching-Hsiang Chang[a], and Mustafa Khammash[a,*]

[a]Department of Biosystems Science and Engineering, ETH Zürich, 4058 Basel, Switzerland
[1]S.A. and M.F. contributed equally to this work
[*]Correspondence: mustafa.khammash@bsse.ethz.ch

Contents

1 Notation & Preliminaries **3**

2 Robust Perfect Adaptation & Model Reduction Theorems **5**
- 2.1 Assumptions 6
- 2.2 Robust Perfect Adaptation 7
- 2.3 Zero-Deficiency of a Network of Reversible Binding and Conversion Reactions 9
- 2.4 Model Reduction 11

3 Mathematical Analysis of the Various Genetically Engineered Controller Circuits **20**
- 3.1 TetR Controller Circuit 20
  - 3.1.1 Full Model Description 20
  - 3.1.2 Model Reduction 20
  - 3.1.3 Monotonicity of Actuation 22
  - 3.1.4 Simulation Results 23
- 3.2 Gal4 Controller Circuit 24
  - 3.2.1 Full Model Description 24
  - 3.2.2 Model Reduction 25
  - 3.2.3 Monotonicity of Actuation 27
  - 3.2.4 Simulation Results 28
- 3.3 intraDD Controller Circuit 29
  - 3.3.1 Full Model Description 29
  - 3.3.2 Model Reduction 30
  - 3.3.3 Monotonicity of Actuation 31
  - 3.3.4 Simulation Results 31
- 3.4 ZF Controller Circuit 32
- 3.5 Inactive-Intein Controller Circuit 33
  - 3.5.1 Full Model Description 33
  - 3.5.2 Model Reduction 34

4 Design and Analysis of Various Integral Controllers Mathematically Realized as Chemical Reaction Networks **36**
- 4.1 Multiple-Dimer Controller Circuit 36
  - 4.1.1 Full Model Description 36
  - 4.1.2 Model Reduction 36
  - 4.1.3 Monotonicity of Actuation 37
  - 4.1.4 Simulation Results 38
- 4.2 Conversion Controller Circuit 39
  - 4.2.1 Full Model Description 39

4.2.2 Model Reduction 39
4.2.3 Monotonicity of Actuation 40
4.2.4 Simulation Results 40
4.3 Trimer Controller Circuit 40
4.3.1 Full Model Description 40
4.3.2 Model Reduction 40
4.3.3 Monotonicity of Actuation 43
4.3.4 Simulation Results 43

5 Design & Implementation Flexibility of Inteins: Examples of Intein-based Integral Controller Circuits 45
5.1 Integral Controllers Based on Transcription Factors & Proteases 45
5.2 Integral Controllers Based on Receptors 47
5.2.1 Full Model Description 47
5.2.2 Model Reduction 50
5.2.3 Monotonicity of Actuation 50
5.2.4 Simulation Results 52

6 A Case Study: Model Reduction in the Stochastic Setting 53

7 Supplementary Figures 55

8 Amino Acid Sequences 62

9 Supplementary Tables 65

1 Notation & Preliminaries

- **Diagonal Operator**: Let $\mathcal{D}$ denote an operator that has two different operations. If it acts on a vector v, it returns a diagonal matrix $D = \mathcal{D}\{v\}$ whose diagonal entries are equal to the vector v, i.e. $D_{ii} = v_i$ and $D_{ij} = 0, \ \forall i \neq j$. If it acts on a matrix A, it returns a column vector v whose entries are equal to the diagonal entries of A, i.e. $v_i = A_{ii}, \ \forall i$.

- **Standard Unit & All-Ones Vectors**: Let e_i denote a column vector, of suitable dimension, whose entries are all equal to 0, except the ith-entry which is equal to 1. Let $\mathbf{1}_n$ denote a column vector of size n whose entries are all ones.

- **Identity & Zero Matrices**: Let I_n denote the identity matrix of size n, and let $0_{n\times m}$ denote the zero matrix of size $n \times m$.

- **Hadamard Product**: The Hadamard (element-wise) product between two vectors is denoted by "$\circ$" and enjoys some useful identities. Let v_1, v_2 and v_3 be three column vectors of the same size, and A and B be two matrices of size $n \times m$, then

  - $v_1 \circ v_2 = \mathcal{D}\{v_1\} v_2$
  - $v_3^T (v_1 \circ v_2) = v_1^T \mathcal{D}\{v_3\} v_2$
  - $v_1 \circ v_2 = \min(v_1, v_2) \circ \max(v_1, v_2)$
  - $\mathcal{D}\{v_1 \circ v_2\} = \mathcal{D}\{v_1\} \mathcal{D}\{v_2\}$
  - $\mathcal{D}\left\{A^T B\right\}^T = \mathbf{1}_n^T (A \circ B)$.

  The proof of these identities are straightforward.

- **Indicator Function**: If A is a matrix whose elements are non-negative, then $\mathbb{1}(A)$ is a binary matrix (its elements are either zeros or ones) of the same size as A whose (i, j) entry is given by

$$\Big(\mathbb{1}(A)\Big)_{ij} := \begin{cases} 1 & \text{if} \quad A_{ij} > 0 \\ 0 & \text{if} \quad A_{ij} = 0. \end{cases}$$

  A useful identity for the indicator function is given by

$$\mathbb{1}(A + B) = \mathbb{1}(A) + \mathbb{1}(B) - \mathbb{1}(A \circ B),$$

  where A and B are two matrices of the same size and whose elements are all non-negative.

- **Selector Matrices**: If M is an $n \times m$ selector matrix, then it is a binary matrix such that only a single entry in each row is 1. A selector matrix selects the entries of a vector, that is $w = Mv$ is a vector whose entries are selected from v and possibly repeated. Here are two useful identities of selector matrices. Let M and N be two selector matrices of size $n \times m$, then

  - $M^T M = \mathcal{D}\left\{\mathbf{1}_n^T M\right\}$
  - If $MN^T = 0$, then $\mathbb{1}\left(M^T M + N^T N\right) = \mathbb{1}\left(M^T M\right) + \mathbb{1}\left(N^T N\right)$.

  The first identity can be seen by partitioning $M = \begin{bmatrix} M_1 & \cdots & M_m \end{bmatrix}$, where M_i is a binary column vector of size n for all $i = 1, \cdots, m$. We have

$$M^T M = \begin{bmatrix} M_1^T \\ \vdots \\ M_m^T \end{bmatrix} \begin{bmatrix} M_1 & \cdots & M_m \end{bmatrix} = \begin{bmatrix} M_1^T M_1 & \cdots & M_1^T M_m \\ \vdots & \ddots & \vdots \\ M_m^T M_1 & \cdots & M_m^T M_m \end{bmatrix}.$$

  For $i \neq j$, $M_i^T M_j = \mathbf{1}_n^T (M_i \circ M_j) = 0$ because M_i and M_j cannot have a 1 at the same entry since each row of M has a single 1 entry and the rest are all zeros. This implies that $M^T M = \mathcal{D}\left\{\begin{bmatrix} M_1^T M_1 & \cdots & M_m^T M_m \end{bmatrix}\right\}$ is a diagonal matrix. But $M_i^T M_i = \mathbf{1}_n (M_i \circ M_i) = \mathbf{1}_n^T M_i, \ \forall i = 1, \cdots, m$ because M_i is a binary vector. Therefore,

$M^T M = \mathcal{D}\left\{\mathbf{1}_n^T M\right\}$. The second identity can be seen by exploiting the first identity and the indicator function identity and by proceeding as follows

$$\begin{aligned}\mathbb{1}\left(M^T M + N^T N\right) &= \mathbb{1}\left(M^T M\right) + \mathbb{1}\left(N^T N\right) - \mathbb{1}\left((M^T M) \circ (N^T N)\right) \\ &= \mathbb{1}\left(M^T M\right) + \mathbb{1}\left(N^T N\right) - \mathbb{1}\left(\mathcal{D}\left\{\mathbf{1}_n^T M\right\} \circ \mathcal{D}\left\{\mathbf{1}_n^T N\right\}\right) \\ &= \mathbb{1}\left(M^T M\right) + \mathbb{1}\left(N^T N\right) - \mathbb{1}\left(\mathcal{D}\left\{(\mathbf{1}_n^T M) \circ (\mathbf{1}_n^T N)\right\}\right) \\ &= \mathbb{1}\left(M^T M\right) + \mathbb{1}\left(N^T N\right) - \mathbb{1}\left(\mathcal{D}\left\{\sum_{i,j=1}^{n} \begin{bmatrix} M_{i1}N_{j1} & \cdots & M_{im}N_{jm}\end{bmatrix}\right\}\right).\end{aligned}$$

But the last term is zero since

$$0 = MN^T = \begin{bmatrix} M_1 & \cdots & M_m \end{bmatrix} \begin{bmatrix} N_1^T \\ \vdots \\ N_m^T \end{bmatrix} = \sum_{j=1}^{m} M_j N_j^T = \sum_{j=1}^{m} \begin{bmatrix} M_{1j} \\ \vdots \\ M_{nj} \end{bmatrix} \begin{bmatrix} N_{1j} & \cdots & N_{nj} \end{bmatrix} = \sum_{j=1}^{m} \begin{bmatrix} M_{1j}N_{1j} & \dots & M_{1j}N_{nj} \\ \vdots & \ddots & \vdots \\ M_{nj}N_{1j} & \dots & M_{nj}N_{nj} \end{bmatrix},$$

and the elements of M and N are all non-negative, which implies that $M_{ij}N_{kj} = 0, \ \forall i, j, k$.

- **Species, Concentrations & Copy Numbers**: Bold uppercase letters denote the names of the species. Their deterministic concentrations are denoted by lowercase letters; whereas their stochastic copy numbers are denoted by uppercase letters. For example, $x(t)$ and $X(t)$ denote the deterministic concentration and stochastic copy numbers of species $\mathbf{X}$ at time t, respectively.

- **Steady-State & Stationary Values**: The steady-state concentration of $\mathbf{X}$ is denoted by $\bar{x}$ in the deterministic setting, while its stationary expectation is denoted by $\mathbb{E}_\pi[X]$ in the stochastic setting.

- **Multiple Reactions:** Let a and b be two column vectors of size n whose entries are all non-negative integers, and let A, B and C be three matrices of size n by m whose entries are all non-negative integers. Furthermore, let $\mathbf{X_1}, \cdots, \mathbf{X_m}$ denote m molecular species, and let $\mathbf{X} := \{\mathbf{X_1}, \cdots, \mathbf{X_m}\}$. Then the following notation

$$\mathrm{A}\,\mathbf{X} + \mathrm{B}\,\mathbf{X} \underset{b}{\overset{a}{\rightleftharpoons}} \mathrm{C}\,\mathbf{X},$$

is a compact representation for the following n reactions

$$\sum_{j=1}^{m} A_{ij}\mathbf{X_j} + \sum_{j=1}^{m} B_{ij}\mathbf{X_j} \underset{b_i}{\overset{a_i}{\rightleftharpoons}} \sum_{j=1}^{m} C_{ij}\mathbf{X_j}\ , \qquad \text{for } i = 1, \cdots, n,$$

where $A_{ij}, B_{ij}, C_{ij}, a_i$ and b_i are the entries of A, B, C, a and b.

- **Abbreviations:** Supplementary Table 1 lists all the abbreviations used in the main text and the supplementary information.

DBD	DNA binding domain	AD	activation domain	RD	repression domain
DD	dimerization domain	VPR	synthetic activator (VP64, p65, Rta)	Int^{C}	intein, carboxyl-terminus
Int^{N}	intein, amino-terminus	GP41-1	intein [1]	NrdJ-1	intein [1]
ZF	Zinc Finger	tTA	tetracycline transactivator	tetR	tetracycline repressor
Gal4	Gal4 transactivator	TF	transcription factor	P2A	self-cleaving peptide
T2A	self-cleaving peptide	NES	nuclear export signal	NLS	nuclear localization signal
N-Deg	N-terminus of split degradation domain	C-Deg	C-terminus of split degradation domain	C-DBD	C-terminus DNA binding domain
S-DBD	split DNA binding domain	SD	split degradation tag	N-Pro	N-terminus protease
C-Pro	C-terminus protease	CP	cytoplasm	PM	plasma membrane
ECS	extracellular space	STD	signal transduction domain	TMD	transmembrane domain
ECD	extracelluar domain	VP16	viral particle 16	RPA	robust perfect adaptation
PID	proportional integral derivative	ODE	ordinary differential equation	DAE	differential algebraic equation
AIF	antithetic integral feedback				

Supplementary Table 1: List of Abbreviations.

2 Robust Perfect Adaptation & Model Reduction Theorems

Following the general framework for biomolecular feedback controllers developed in [2], consider a general network, depicted in Supplementary Fig. 1, comprised of L species $\mathbf{X} := \{\mathbf{X_1}, \cdots, \mathbf{X_L}\}$ that react with each other through K reaction channels labeled as $\mathcal{R} := \{\mathcal{R}_1, \cdots, \mathcal{R}_K\}$. Each reaction $\mathcal{R}_k$ has a stoichiometry vector denoted by $\zeta_k \in \mathbb{Z}^L$

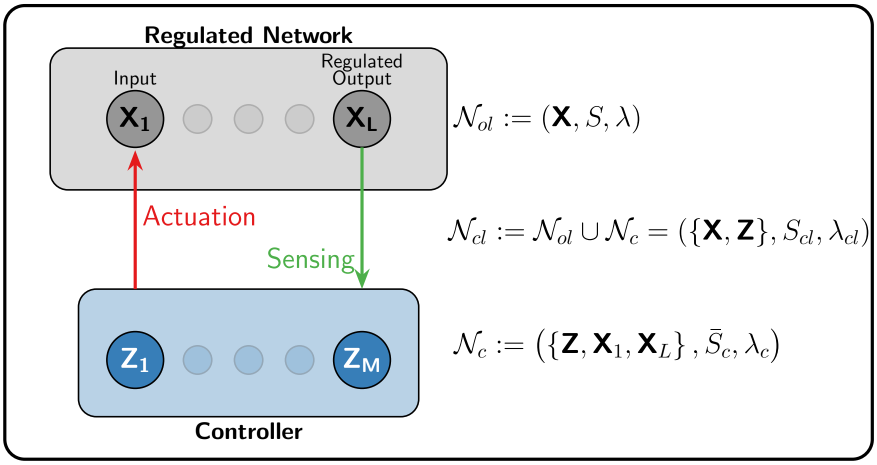

Supplementary Figure 1: A general framework for biomolecular feedback controllers adopted from [2]. The closed-loop network, denoted by $\mathcal{N}_{cl}$, is comprised of the regulated network (or open-loop network) $\mathcal{N}_{ol}$ interconnected in a feedback configuration with the controller network $\mathcal{N}_c$. Each network is fully represented by the list of species, stoichiometry matrix and propensity function. The controller network communicates with the regulated network by sensing the regulated output species $\mathbf{X_L}$ and by the actuation (influencing the production and/or degradation) of the input species $\mathbf{X_1}$.

and a propensity function $\lambda_k : \mathbb{R}^L_{\geq 0} \to \mathbb{R}_{\geq 0}$. Let $S := \begin{bmatrix} \zeta_1 & \cdots & \zeta_K \end{bmatrix} \in \mathbb{Z}^{L \times K}$ denote the stoichiometry matrix, and let $\lambda := \begin{bmatrix} \lambda_1 & \cdots & \lambda_K \end{bmatrix}^T : \mathbb{R}^L_{\geq 0} \to \mathbb{R}^K_{\geq 0}$ denote the (vector-valued) propensity function. We shall refer to this network as the regulated network or open-loop network which is fully characterized by the triplet $\mathcal{N}_{ol} = (\mathbf{X}, S, \lambda)$. Furthermore, consider another chemical reaction network comprised of M species $\mathbf{Z} := \{\mathbf{Z_1}, \cdots, \mathbf{Z_M}\}$ whose objective is to act as a feedback controller that regulates the species $\mathbf{X_L}$ to some prescribed concentration referred to as the *setpoint* or *reference*. We assume that all the species in the regulated network are not directly accessible by the controller except for two species. In particular and without loss of generality, the first species $\mathbf{X_1}$ is assumed to be the only species in the regulated network that is accessible for actuation by the controller; whereas, the last species $\mathbf{X_L}$ is assumed to be the only species in the regulated network that is sensed by the controller. To this end, $\mathbf{X_1}$ and $\mathbf{X_L}$ are respectively referred to as the input and regulated output species. The controller species are allowed to react with each other and with the input/output species of the regulated network through K_c reaction channels labeled as $R^c := \{\mathcal{R}^c_1, \cdots, \mathcal{R}^c_{K_c}\}$. Let $\bar{S}_c \in \mathbb{Z}^{(M+2) \times K_c}$ and $\lambda_c : \mathbb{R}^{M+2}_{\geq 0} \to \mathbb{R}^{K_c}_{\geq 0}$ respectively denote the stoichiometry matrix and propensity function of the controller network. Since the controller reactions $\mathcal{R}^c$ involve the controller species $\mathbf{Z}$ and the input/output species $\mathbf{X_1}/\mathbf{X_L}$ of the regulated network, the stoichiometry matrix $\bar{S}_c$ can be partitioned as

$$\bar{S}_c := \begin{bmatrix} S_1 \\ S_L \\ S_c \end{bmatrix},$$

where S_1 and $S_L \in \mathbb{Z}^{1 \times K_c}$ encrypt the stoichiometry coefficients of the input and output species $\mathbf{X_1}$ and $\mathbf{X_L}$, respectively, among the controller reaction channels $\mathcal{R}^c$. Furthermore, $S_c \in \mathbb{Z}^{M \times K_c}$ encrypts the stoichiometry coefficients of the controller species $\mathbf{Z_1}, \cdots, \mathbf{Z_M}$. The network involving the species $\mathbf{Z}$, $\mathbf{X_1}$ and $\mathbf{X_L}$ reacting among each other through the reaction channels $\mathcal{R}^c$ shall be referred to as the controller network and is fully represented by the triplet $\mathcal{N}_c = \left(\{\mathbf{Z}, \mathbf{X_1}, \mathbf{X_L}\}, \bar{S}_c, \lambda_c\right)$. Finally, the closed-loop system constitutes the regulated network augmented with the controller network so that it includes all the species $\mathbf{X} \cup \mathbf{Z}$ and reactions $\mathcal{R} \cup \mathcal{R}^c$. Thus, the closed-loop network $\mathcal{N}_{cl} := \mathcal{N}_{ol} \cup \mathcal{N}_c$ can be fully represented by the closed-loop stoichiometry matrix S_{cl} and propensity function λ_{cl} given by

$$
S_{cl} = \begin{array}{c}
\overbrace{\mathcal{R}_1 \;\; \mathcal{R}_2 \;\; \cdots \;\; \mathcal{R}_K}^{\mathcal{R}} \quad \overbrace{\mathcal{R}_1^c \;\; \mathcal{R}_2^c \;\; \cdots \;\; \mathcal{R}_{K_c}^c}^{\mathcal{R}^c} \\
\left[\begin{array}{c|c}
 & S_1 \\ \cdots & \text{- - - - - -} \\
S & 0 \\
 & \text{- - - - - -} \\
 & S_L \\ \hline
0 & S_c
\end{array}\right]
\end{array}
\begin{array}{l}
\left.\begin{array}{l}\mathbf{X}_1 \\ \mathbf{X}_2 \\ \vdots \\ \mathbf{X}_{L-1} \\ \mathbf{X}_L\end{array}\right\} \mathbf{X} \\
\left.\begin{array}{l}\mathbf{Z}_1 \\ \vdots \\ \mathbf{Z}_M\end{array}\right\} \mathbf{Z}
\end{array}
\qquad (1)
$$

$$
\lambda_{cl}(x,z) = \left[\begin{array}{c|c} \lambda^T(x) & \lambda_c^T(x_1, x_L, z) \end{array}\right]^T.
$$

2.1 Assumptions

In this section, we state various assumptions that describe particular structures of the controller networks. These assumptions will be used for establishing RPA and for deriving a reduced model as well.

Assumption 1. *The controller network includes the following reactions:*

- *The production of the controller species* $\mathbf{Z_i}$ *for* $i = 1, \cdots, M$ *are constitutive and/or catalytically driven by the output species* $\mathbf{X_L}$*. That is, using the compact notation for reactions presented in Section 1, we have*

$$\mathcal{R}_s : \quad \varnothing \xrightarrow{\mu + \theta x_L} \mathbf{Z} \quad (M - \textbf{Setpoint/Sensing Reactions}),$$

where $\mu = \begin{bmatrix}\mu_1 & \cdots & \mu_M\end{bmatrix}^T$ *and* $\theta = \begin{bmatrix}\theta_1 & \cdots & \theta_M\end{bmatrix}^T$ *with* $\mu_i \geq 0$ *denoting the constitutive production rate of* $\mathbf{Z_i}$ *while* $\theta_i x_L \geq 0$ *denoting the catalytic production rate of* $\mathbf{Z_i}$ *which is proportional to the output species* $\mathbf{X_L}$*, for* $i = 1, \cdots, M$*. Furthermore, at least one* μ_i *and one* θ_j *with* $i \neq j$ *are non-zeros.*

- *The controller actuates the input species* $\mathbf{X_1}$ *via a production and/or a removal reaction, that is*

$$
\begin{aligned}
\mathcal{R}_a^+ : &\quad \varnothing \xrightarrow{u^+} \mathbf{X_1} \quad (\textbf{Production-Actuation Reaction}) \\
\mathcal{R}_a^- : &\quad \mathbf{X_1} \xrightarrow{u^-} \varnothing \quad (\textbf{Removal-Actuation Reaction}),
\end{aligned}
$$

where the actuation rates $u^{\pm} = h^{\pm}(z, x_L)$ *are functions of the controller and output species.*

Assumption 2. *The controller network respects the following structure:*

- *The various reactions between a pair of controller species are either irreversible sequestration reactions (at least one) or reversible binding reactions. That is, we have*

$$
\begin{aligned}
\mathcal{R}_Q : &\quad Q_1\mathbf{Z} + Q_2\mathbf{Z} \xrightarrow{\eta v} Q_3\mathbf{Z} \quad (m - \textbf{Sequestration Reactions}) \\
\mathcal{R}_B : &\quad B_1\mathbf{Z} + B_2\mathbf{Z} \underset{d}{\overset{a}{\rightleftharpoons}} B_3\mathbf{Z} \quad (p - \textbf{Reversible Binding Reactions}),
\end{aligned}
$$

where $\eta > 0$ *is a scalar;* v *is an* $m \times 1$ *positive vector such that* ηv_ℓ *denotes the sequestration rate of the* ℓ^{th}*-sequestration reaction for* $\ell = 1, 2, \cdots, m$ *with* $m \geq 1$*;* a *and* d *are* $p \times 1$ *positive vectors such that* a_ℓ *and* d_ℓ *denote the association and dissociation rates of the* ℓ^{th}*-binding reaction for* $\ell = 1, 2, \cdots, p$ *with* $p \geq 0$*. Furthermore,* Q_1 *and* Q_2 *are* $m \times M$ *selector matrices (see Section 1) and* Q_3 *is a binary* $m \times M$ *matrix such that their rows are pairwise different, more precisely, we have* $Q_1 \circ Q_2 = Q_2 \circ Q_3 = Q_1 \circ Q_3 = 0$ *(i.e. a controller species* $\mathbf{Z_i}$ *can only be sequestered by another controller species* $\mathbf{Z_j}$ *to yield yet another controller species) and the rows of* $\begin{bmatrix}Q_1 & Q_2\end{bmatrix}$ *cannot be repeated (i.e. the same reactants cannot participate in multiple sequestration reactions). Note that the ordering of the reactants of the sequestration reactions are such that* $Q_1 Q_2^T = 0$ *(i.e. if a species is selected by* Q_1*, it cannot be selected by* Q_2 *and vice versa). Since there exists at least one sequestration reaction, we assume without loss of generality that the first sequestration reaction is*

$$\mathcal{R}_{Q_1} : \quad \mathbf{Z_1} + \mathbf{Z_2} \xrightarrow{\eta} \mathbf{Z_3} + \cdots,$$

that is $e_1^T Q_1 = e_1^T, e_1^T Q_2 = e_2^T$ *and* $e_1^T Q_3 e_3 = 1$*. Lastly,* B_1 *and* B_2 *are* $p \times M$ *selector matrices and* B_3 *is a binary* $p \times M$ *matrix such that* $B_1 \circ B_3 = B_2 \circ B_3 = 0$ *(i.e. a complex is a different species from its constituents). Note that the rows of* $B_1 + B_2$ *cannot be repeated (i.e. the same reactants cannot participate in multiple binding reactions), and* $e_i^T(B_1 + B_2) \neq e_j^T B_3$ *for all* $i, j = 1, \cdots, p$ *(i.e. products of binding reactions cannot bind with each other).*

- *Controller species that are not sequestered by any other controller species are degraded. That is*

$$\mathcal{R}_D: \quad Q_0\,\mathbf{Z} \xrightarrow{\delta_0} \varnothing \quad (m_0 - \textbf{Degradation Reactions}),$$

where $\delta_0 > 0$ *is a scalar denoting the degradation rate. Furthermore,* Q_0 *is an* $m_0 \times M$ *selector matrix with* $m_0 \geq 0$ *such that* $Q_0 Q_1^T = Q_0 Q_2^T = 0$ *(i.e. only species that are not sequestered are degraded). Note that the rows of* Q_0 *are not repeated (i.e. the same controller species cannot be involved in multiple degradation reactions).*

- *Controller species may undergo reversible conversion reactions given by*

$$\mathcal{R}_C: \quad C_1\,\mathbf{Z} \underset{c_2}{\overset{c_1}{\rightleftharpoons}} C_2\,\mathbf{Z} \quad (p_0 - \textbf{Reversible Conversion Reactions}),$$

where c_1 *and* c_2 *are* $p_0 \times 1$ *positive vectors such that* $(c_1)_\ell$ *and* $(c_2)_\ell$ *denote the conversion rates of the* ℓ^{th}*-conversion reaction for* $\ell = 1, 2, \cdots, p_0$*. Furthermore,* C_1 *and* C_2 *are selector matrices such that* $C_1 \circ C_2 = 0$ *(i.e. a controller species is converted to a different controller species).*

- *The controller species are all diluted. That is*

$$\mathcal{R}_\delta: \quad \mathbf{Z} \xrightarrow{\delta} \varnothing \quad (M - \textbf{Dilution Reactions}),$$

where $\delta \geq 0$ *is a scalar representing the dilution rate of all the controller species.*

2.2 Robust Perfect Adaptation

Under Assumption 1, the controller stoichiometry matrix S_c in (1) can be partitioned as

$$S_c = \begin{array}{c} \begin{array}{cccc} \mathcal{R}_a^+ \mathcal{R}_a^- & \mathcal{R}_s & \mathcal{R}_r \end{array} \\ \left[\begin{array}{cc|c|c} 0 & 0 & & \\ \vdots & \vdots & I_M & S_r \\ 0 & 0 & & \end{array}\right] \end{array}, \tag{2}$$

where S_r denote the remaining partition of S_c associated with all the controller reactions excluding the setpoint/sensing reactions and actuation reactions given in Assumption 1. The following theorem provides a guarantee for achieving RPA in the regulated output species $\mathbf{X_L}$.

Supplementary Theorem 1 (RPA).
Consider a controller network satisfying Assumption 1. If the closed-loop system is stable and there exists a vector $q := \begin{bmatrix} q_1 & \cdots & q_M \end{bmatrix}^T$ *of size* M *satisfying* $q^T S_r = 0$*, then the controller achieves Robust Perfect Adaptation (RPA) such that the steady-state concentration of the regulated output species* $\mathbf{X_L}$ *is given by*

$$\bar{x}_L := \lim_{t \to \infty} x_L(t) = -\frac{q^T \mu}{q^T \theta}. \tag{3}$$

Proof. Under Assumption 1, the controller propensity function λ_c in (1) can be partitioned as

$$\lambda_c(x_1, x_L, z) = \left[\begin{array}{c} h^+(z, x_L) \\ h^-(z, x_L) x_1 \\ \hline \mu + \theta x_L \\ \hline \lambda_r(z) \end{array}\right] \begin{array}{l} \mathcal{R}_a^+ \\ \mathcal{R}_a^- \\ \mathcal{R}_s \\ \mathcal{R}_r \end{array} \tag{4}$$

where λ_r denotes the propensity function associated with the controller reactions $\mathcal{R}_r$ excluding the setpoint/sensing reactions and acutation reactions given in Assumption 1. By exploiting equations (1-4), the closed-loop dynamics can be written as

$$\begin{bmatrix} \dot{x} \\ \dot{z} \end{bmatrix} = S_{cl}\lambda_{cl}(x,z) = \left[\begin{array}{c|cc|c|c} & 1 & -1 & & \\ & 0 & 0 & & \\ S & \vdots & \vdots & 0 & 0 \\ & 0 & 0 & & \\ & 0 & 0 & & \\ \hline & 0 & 0 & & \\ 0 & \vdots & \vdots & I_M & S_r \\ & 0 & 0 & & \end{array}\right] \left[\begin{array}{c} \lambda(x) \\ \hline h^+(z,x_L) \\ h^-(z,x_L)x_1 \\ \hline \mu + \theta x_L \\ \hline \lambda_r(z) \end{array}\right]$$

$$= \begin{bmatrix} S\lambda(x) + \Big(h^+(z,x_L) - h^-(z,x_L)x_1\Big)e_1 \\ \mu + \theta x_L + S_r\lambda_r(z) \end{bmatrix}.$$

Assuming that the closed-loop system is stable, the dynamics at steady state converge to the fixed point that satisfies the following nonlinear algebraic equation

$$\begin{cases} S\lambda(\bar{x}) + \Big(h^+(\bar{z},\bar{x}_L) - h^-(\bar{z},\bar{x}_L)\bar{x}_1\Big)e_1 = 0 \\ \mu + \theta\bar{x}_L + S_r\lambda_r(\bar{z}) = 0. \end{cases}$$

Multiplying both sides of the second equation by q^T and invoking the condition that $q^T S_r = 0$ yield the following expression for the steady-state output concentration

$$q^T(\mu + \theta\bar{x}_L) = 0 \quad \Longrightarrow \bar{x}_L = -\frac{q^T\mu}{q^T\theta},$$

which finishes the proof since $\bar{x}_L$ is independent of the initial conditions and parameters of the regulated network. □

Note that this theorem can be easily extended to the stochastic setting by looking at the mean dynamics of Z that are given by

$$\frac{d\mathbb{E}[Z]}{dt} = \mu + \theta\mathbb{E}[X_L] + S_r\mathbb{E}[\lambda_r(Z)].$$

An argument similar to the deterministic setting can be invoked at stationarity where, assuming ergodicity, we have

$$q^T(\mu + \theta\mathbb{E}_\pi[X_L]) = 0 \quad \Longrightarrow \mathbb{E}_\pi[X_L] = -\frac{q^T\mu}{q^T\theta},$$

since $q^T S_r = 0$.

Next, we prove Theorem 1 (of the main text) by applying Supplementary Theorem 1 to the closed-loop network of Fig. 2 in the main text where the controller is particularly intein-based.

Proof of Theorem 1 of the main text.

Proof. Let q_i^+ and q_i^- respectively denote the number of active IntC and IntN segments present in controller species $\mathbf{Z}_i$ for $i = 1, \cdots M$, and define $q^+ := \begin{bmatrix} q_1^+ & \cdots & q_M^+ \end{bmatrix}^T$, $q^- := \begin{bmatrix} q_1^- & \cdots & q_M^- \end{bmatrix}^T$ and $q := q^+ - q^-$. We now explicitly write the reactions that are listed as rules in Fig. 2 in the main text. Adopting the compact notation presented in Section 1, we list the allowed reactions in Supplementary Table 2 accompanied with their associated stoichiometry matrices and propensity functions. Observe that these reactions clearly satisfy Assumption 1.

Note that "$\circ$" denotes the Hadamard (element-wise) product between two vectors. The rate parameters $\mu, \theta, \bar{\eta}, c, a, d$ are all nonnegative vectors and $Q_1, Q_2, C_1, C_2, B_1, B_2, B_3$ are all selector matrices (see Section 1). According to Reaction Rule 2 in Fig. 2 of the main text, the selector matrices Q_1 and Q_2 select controller species from the $\mathcal{C}$- and $\mathcal{N}$-classes, respectively; whereas Q_3 is allowed to select multiple controller species at least one of which belongs to

Rule #	Reactions	Stoichiometry Matrix	Propensity Function
Rule 1	$\emptyset \xrightarrow{\mu+\theta x_L} \mathbf{Z}$	I_M	$\mu + \theta x_L$
Rule 2	$Q_1\,\mathbf{Z} + Q_2\,\mathbf{Z} \xrightarrow{\bar{\eta}} Q_3\,\mathbf{Z}$	$S_Q := (Q_3 - Q_2 - Q_1)^T$	$\bar{\eta} \circ (Q_1 z) \circ (Q_2 z)$
Rule 3	$\emptyset \xrightarrow{h^+(z,x_L)} \mathbf{X_1} \xrightarrow{h^-(z,x_L)} \emptyset$	$\pm\begin{bmatrix}1 & 0 & \cdots & 0\end{bmatrix}^T$	$h^+(z,x_L), \quad h^-(z,x_L)x_1$
Rule 4	$C_1\,\mathbf{Z} \xrightarrow{c} C_2\,\mathbf{Z}$	$S_C := (C_2 - C_1)^T$	$c \circ (C_1 z)$
Rules 5,6,7	$B_1\,\mathbf{Z} + B_2\,\mathbf{Z} \underset{d}{\overset{a}{\rightleftharpoons}} B_3\,\mathbf{Z}$	$\pm S_B := \pm(B_3 - B_2 - B_1)^T$	$a \circ (B_1 z) \circ (B_2 z), \quad d \circ (B_3 z)$

Supplementary Table 2: List of allowed controller reactions that respect the rules given in Fig. 2 of the main text.

the $\mathcal{S}$-class. These reactions (rule 2) encode for intein-splicing reactions where active IntN and IntC segements are stoichiometrically inactivated. Mathematically, this means that

$$q^{+T} S_Q = q^{-T} S_Q \qquad \implies q^T S_Q = 0.$$

Furthermore, according to reaction rule 4 in Fig. 2 of the main text, the selector matrices C_1 and C_2 select controller species from the same class. These conversion reactions (rule 4) preserve the number of IntC or IntN. Mathematically this means that

$$q^{+T} S_C = q^{-T} S_C = 0 \qquad \implies q^T S_C = 0.$$

Lastly, the last row of Supplementary Table 2 compactly encodes for the reaction rules 5,6 and 7 in Fig. 2 of the main text. These reversible binding reactions, similar to the conversion reactions, preserve the number of IntC or IntN. Mathematically this means that

$$q^{+T} S_B = q^{-T} S_B = 0 \qquad \implies q^T S_B = 0.$$

To apply Supplementary Theorem 1, we note that the partition S_r of the controller stoichiometry matrix S_c given in (2) can be further partitioned to

$$S_r = \begin{bmatrix} S_Q & S_C & S_B & -S_B & S_F \end{bmatrix},$$

where S_F represents the stoichiometry matrix of remaining free reactions that follow rule 8 in Fig. 2 of the main text which involves species belonging to the $\mathcal{S}$-class that have no active inteins. Mathematically, this means that

$$q^T S_F = 0,$$

since if $\mathbf{Z}_i \in \mathcal{S}$ then $q_i = 0$. Therefore, we have $q^T S_r = 0$ and, by Supplementary Theorem 1, the intein-based controller respecting the rules given in Fig. 2 of the main text achieves RPA as long as the closed-loop network is stable. □

Note that the theorem also applies in the stochastic setting as previously mentioned.

2.3 Zero-Deficiency of a Network of Reversible Binding and Conversion Reactions

Consider a chemical reaction network $\mathcal{N}_{BC}$ that is comprised of M species $\mathbf{Z} := \{\mathbf{Z_1}, \cdots, \mathbf{Z_M}\}$ reacting among each other solely via reversible binding and conversion reaction channels. That is, using the compact reaction notation introduced in Section 1 we write

$$\mathcal{N}_{BC} : \begin{cases} \mathrm{B}_1\mathbf{Z} + \mathrm{B}_2\mathbf{Z} \underset{d}{\overset{a}{\rightleftharpoons}} \mathrm{B}_3\mathbf{Z} & (p - \text{Reversible Binding Reactions}) \\ \mathrm{C}_1\mathbf{Z} \underset{c_2}{\overset{c_1}{\rightleftharpoons}} \mathrm{C}_2\mathbf{Z} & (p_0 - \text{Reversible Conversion Reactions}), \end{cases} \tag{5}$$

where $B_1, B_2 \in \mathbb{N}^{p\times M}$ and $C_1, C_2 \in \mathbb{N}^{p_0\times M}$ are selector matrices (see Section 1), $B_3 \in \mathbb{N}^{p\times M}$ is a binary matrix and $a, d \in \mathbb{R}^p_{>0}$ and $c_1, c_2 \in \mathbb{R}^{p_0}_{>0}$ are positive vectors. The following Lemma will be useful for the proof of the model reduction result established in the subsequent section.

Lemma 1. *Consider the reversible binding-conversion network $\mathcal{N}_{BC}$ in (5). Let $S_B := (B_3 - B_2 - B_1)^T$ and $S_C := (C_2 - C_1)^T$ respectively denote the stoichiometry matrices of the binding and conversion reactions. Under the following assumptions:*

- *Binding products cannot be binding reactants, i.e. $e_i^T(B_1 + B_2) \neq e_j^T B_3, \forall i, j \in \{1, \cdots, p\}$.*

- *A reactant complex cannot participate in more than one binding reaction, i.e.* $e_i^T(B_1 + B_2) \neq e_j^T(B_1 + B_2), \forall i \neq j \in \{1, \cdots, p\}$*,*

we have that the deficiency of the network is zero if and only if S_B *is full column rank and* S_C *is linearly independent of* S_B*, i.e.*

$$\text{rank}(S_B) = p \qquad \textit{and} \qquad \text{rank}\left(\left[\begin{array}{c|c} S_B & S_C \end{array}\right]\right) = \text{rank}(S_B) + \text{rank}(S_C).$$

The lemma essentially states that a network of reversible binding and conversion reactions has zero deficiency if and only if all binding and conversion reactions are not a linear combination of other binding reactions. We provide the proof next.

Proof. A graphical depiction of network $\mathcal{N}_{BC}$ is shown in Supplementary Fig. 2 where the complexes of the various reactions can be classified into two main categories. The first category (blue complexes in Supplementary Fig. 2) constitutes the binding reactant complexes, i.e. $e_i^T(B_1\mathbf{Z} + B_2\mathbf{Z})$ for $i = 1, \cdots, p$. The second category (red and green complexes in Supplementary Fig. 2) constitutes both the binding product complexes, $e_i^T B_3\mathbf{Z}$ for $i = 1, \cdots, p$, and the conversion complexes, $e_i^T C_1\mathbf{Z}$ and $e_i^T C_2\mathbf{Z}$ for $i = 1, \cdots, p_0$. The binding reactant complexes, $e_i^T(B_1\mathbf{Z} + B_2\mathbf{Z})$ are comprised of two different or similar species (e.g. homo/heterodimerization reactions); whereas, the binding product complexes may be formed of single species (if the row of B_3 corresponding to the reaction yielding the binding product has only one non-zero element) or multiple species (if the row of B_3 corresponding to the reaction yielding the binding product has multiple non-zero elements). Hence, complexes in the second category can be divided into two subcategories: (1) complexes comprised of single species (red complexes in Supplementary Fig. 2 which can either be the products of binding reactions or species participating in conversion reactions or both, and (2) complexes comprised of multiple species (green complexes in Supplementary Fig. 2) which cannot participate in conversion reactions since conversions can only happen between complexes comprised of single species.

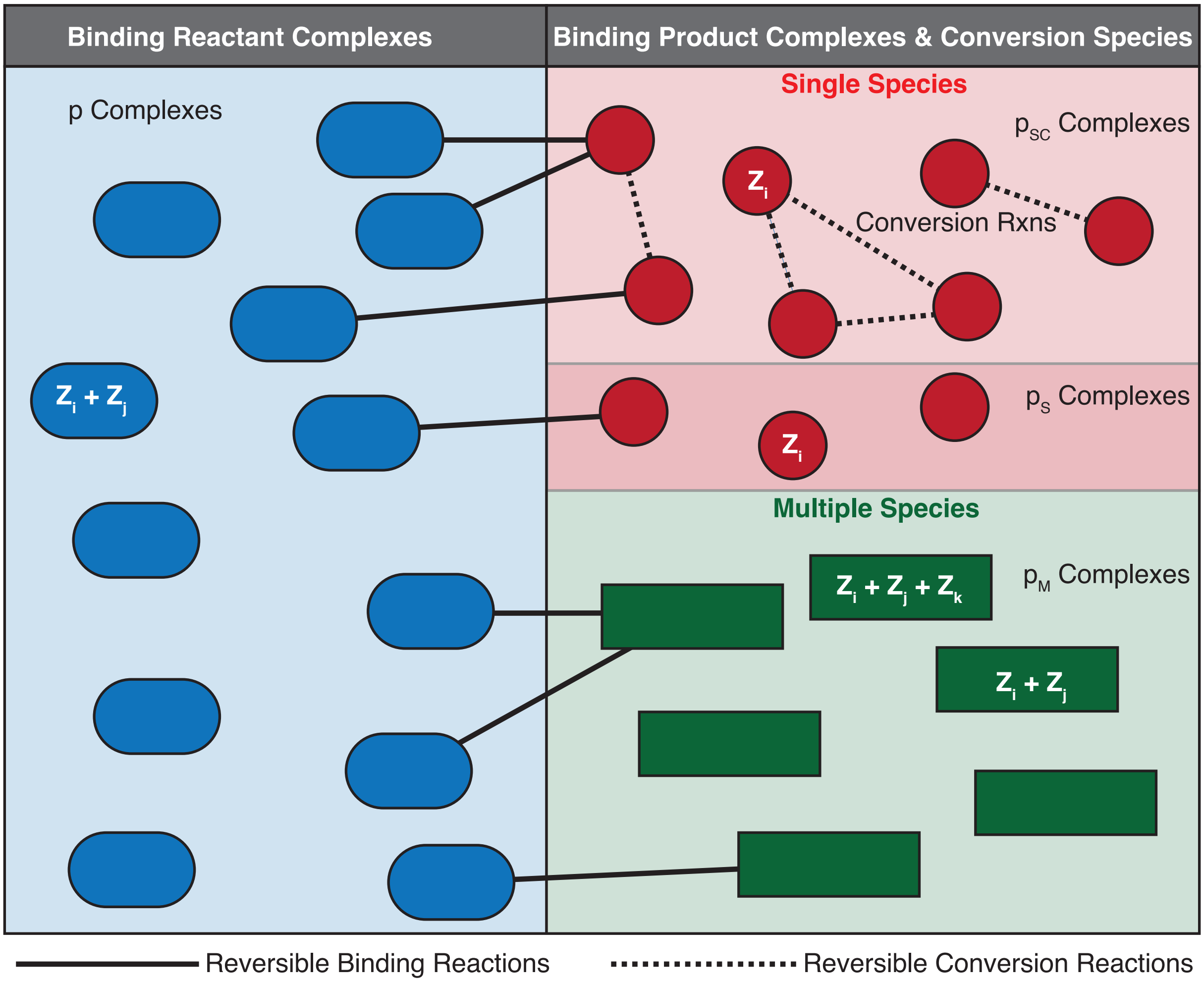

Supplementary Figure 2: Binding-Conversion Network.

Let n, ℓ and s denote the number of complexes, number of linkage classes and dimension of the stoichiometry subspace of the binding-conversion network $\mathcal{N}_{BC}$. Since a reactant complex cannot participate in more than one binding reaction, then there are p reactant complexes (as many as the number of binding reactions). Furthermore, Let p_{SC} (resp. p_S) denote the number of binding product complexes that are comprised of single species and participate (resp. do not participate) in conversion reactions, and let p_M denote the binding product complexes that are comprised of multiple species. The total number of complexes in the network is thus given by

$$n = p + p_S + p_M + p_{SC}.$$

Let ℓ_C denote the number of linkage classes among the reversible conversion reactions alone (top red box in Supplementary Fig. 2). Hence, the number of linkage classes ℓ' of all the binding product complexes (red and green in Supplementary Fig. 2) without the reversible binding reactions (solid lines in Supplementary Fig. 2) is given by

$$\ell' = \ell_C + p_S + p_M.$$

Now adding the binding reactant complexes and reversible binding reactions does not decrease the number of linkage classes since each binding reactant complex can only participate in a single binding reaction (i.e. separate linkage classes will not be connected when adding the binding reactions), and thus $\ell = \ell'$. The dimension of the stoichiometry subspace s is given by

$$s = \text{rank}\left(\left[\begin{array}{c|c} S_B & S_C \end{array}\right]\right) = \text{rank}(S_B) + \text{rank}(S_C) - \dim\left\{\text{Im}(S_B) \cap \text{Im}(S_C)\right\},$$

where "Im(.)" denotes the image of a matrix and "dim{.}" denotes the dimension of a subspace. However, we have that $\text{rank}(S_C) = p_{SC} - \ell_C$. This can be shown by first arranging the species $\mathbf{Z_1}, \cdots, \mathbf{Z_M}$ such that the conversion stoichiometry matrix S_C can be partitioned as

$$S_C = \left[\begin{array}{cccc} S_C^1 & 0 & \cdots & 0 \\ 0 & S_C^2 & \ddots & \vdots \\ \vdots & \ddots & \ddots & 0 \\ 0 & \cdots & 0 & S_C^{\ell_C} \\ \hline \\ 0 & \cdots & \cdots & 0 \\ \\ \end{array}\right] \quad \text{with} \quad S_C^i \in \mathbb{N}^{p_i \times \tilde{p}_i} \text{ for } i = 1, \cdots, \ell_c, \quad \text{such that } \sum_{i=1}^{\ell_C} p_i = p_{SC} \quad \text{and} \quad \sum_{i=1}^{\ell_C} \tilde{p}_i = p_0,$$

where each block S_C^i correspond to a separate linkage class, and the last zero rows corresponds to the species among $\mathbf{Z_1}, \cdots, \mathbf{Z_M}$ that do not participate in any conversion reaction. But each block matrix S_C^i is rank deficient by exactly one because within its associated linkage class, the number of independent conversion reactions is less than the number of species by one. Then, we have

$$\text{rank}(S_C) = \sum_{i=1}^{\ell_C} \text{rank}(S_C^i) = \sum_{i=1}^{\ell_C} (p_i - 1) = p_{SC} - \ell_C.$$

Therefore the deficiency of the full network $\mathcal{N}_{BC}$ is given by

$$\delta = n - \ell - s = p - \text{rank}(S_B) + \dim\left\{\text{Im}(S_B) \cap \text{Im}(S_C)\right\}.$$

Clearly, if $p = \text{rank}(S_B)$ and $\text{rank}\left(\left[\begin{array}{c|c} S_B & S_C \end{array}\right]\right) = \text{rank}(S_B) + \text{rank}(S_C)$, then $\delta = 0$ which completes the proof for sufficiency. To complete the proof for necessity, observe that the only way δ can be zero is if

$$\text{rank}(S_B) = p + \dim\left\{\text{Im}(S_B) \cap \text{Im}(S_C)\right\}.$$

But $\text{rank}(S_B) \leq p$, then $\text{rank}(S_B)$ must be equal to p and $\text{Im}(S_B) \cap \text{Im}(S_C) = \{0\}$ and thus $\text{rank}\left(\left[\begin{array}{c|c} S_B & S_C \end{array}\right]\right)$ must be equal to $\text{rank}(S_B) + \text{rank}(S_C)$. □

2.4 Model Reduction

Consider the closed-loop network depicted in Supplementary Fig. 1 where the controller network satisfies Assumptions 1 and 2. Let $S_Q := (Q_3 - Q_2 - Q_1)^T$, $S_B := (B_3 - B_2 - B_1)^T$, $S_D := -Q_0^T$, $S_C := (C_2 - C_1)^T$ and $S_\delta := -I_M$ respectively denote the partitions of the controller stoichiometry matrix associated with the irreversible sequestration, reversible binding, degradation, conversion and dilution reactions. Furthermore, let q^+, q^- and q^0 be three non-negative vectors of size M satisfying the following conditions.

Condition 1.

$$\begin{cases} {q^{\pm}}^T S_B = {q^0}^T S_B = {q^{\pm}}^T S_C = {q^0}^T S_C = {q^{\pm}}^T S_D = 0 \\ \mathbb{1}\left(q^+ {q^-}^T\right) = Q_1^T Q_2, \quad \mathbb{1}\left(q^+\right) = \mathbb{1}\left(Q_1^T \mathbf{1}_m\right) \quad and \quad \mathbb{1}\left(q^-\right) = \mathbb{1}\left(Q_2^T \mathbf{1}_m\right) \\ \min\left(Q_1 q^+, Q_2 q^-\right) = -S_Q^T q^{\pm} = S_Q^T q^0 \\ \max\left(Q_1 q^+, Q_2 q^-\right) = v. \end{cases} \tag{6}$$

Condition 1 is invoked in the following theorem which provides a *structural* model reduction result.

Supplementary Theorem 2 (Model Reduction). *Consider the controller network satisfying Assumptions 1 and 2. Let* q^+*,* q^-*,* $q^0 = \begin{bmatrix} 0 & 0 & \star & \end{bmatrix}^T$*, with* $q^0 e_3 > 0$*, be three non-negative vectors of size* M *satisfying Condition 1. Introduce the following state transformations and nonnegative scalar parameters*

$$z^i := {q^i}^T z, \qquad \mu^i := {q^i}^T \mu, \qquad \theta^i := {q^i}^T \theta, \qquad i \in \{+,-,0\}. \tag{7}$$

Let $\lambda_B(z) := a \circ (B_1 z) \circ (B_2 z) - d \circ (B_3 z)$ *and* $\lambda_C(z) := c_1 \circ (C_1 z) - c_2 \circ (C_2 z)$ *respectively denote the propensity functions of the reversible binding and conversion reactions, and define the following matrices and vectors*

$$\begin{cases} \bar{C} := \begin{bmatrix} I_3 & 0_{3\times(M-3)} \end{bmatrix} \\ \tilde{C} := \begin{bmatrix} 0_{(M-3)\times 3} & I_{M-3} \end{bmatrix} \end{cases}, \qquad q_{tot} = \begin{bmatrix} q^+ & q^- & q^0 \end{bmatrix}, \qquad z^{tot} := \begin{bmatrix} z^+ \\ z^- \\ z^0 \end{bmatrix}, \qquad \begin{cases} W_1 := \left(\bar{C} q_{tot}\right)^{-1} \bar{C} \\ W_2 := \tilde{C}\Big(I_M - q_{tot}\left(\bar{C} q_{tot}\right)^{-1} \bar{C}\Big). \end{cases}$$

If $a = \tilde{a}/\epsilon$*,* $d = \tilde{d}/\epsilon$*,* $c_1 = \tilde{c}_1/\epsilon$*,* $c_2 = \tilde{c}_2/\epsilon$*,* S_B *is full-column rank, the columns of* S_C *are linearly independent from those of* S_B *and* $p + rank(S_C) = M - 3$*, then as* $\epsilon \to 0$*, the controller dynamics reduce to*

$$\begin{cases} \dot{z}^+ = \mu^+ + \theta^+ x_L - \eta z^+ z^- - \delta z^+ \\ \dot{z}^- = \mu^- + \theta^- x_L - \eta z^+ z^- - \delta z^- \\ \dot{z}^0 = \mu^0 + \theta^0 x_L + \eta z^+ z^- - (\delta + \delta_0) z^0 + \delta_0 \left(\mathbb{1}\left(q^+ + q^-\right) \circ q^0\right)^T \psi(z^{tot}) \end{cases} \tag{8}$$

$$\text{Control Action:} \quad u^{\pm} = \mathcal{U}^{\pm}\left(z^{tot}, x_L\right) := h^{\pm}\Big(\psi(z^{tot}), x_L\Big),$$

where $\psi(z^{tot}) := W_1^T z^{tot} + W_2^T \tilde{z} > 0$ *and* $\tilde{z} > 0$ *solves the following algebraic equation in terms of* z^{tot}

$$\tilde{C} S_B \tilde{\lambda}_B\left(W_1^T z^{tot} + W_2^T \tilde{z}\right) + \tilde{C} S_C \tilde{\lambda}_C\left(W_1^T z^{tot} + W_2^T \tilde{z}\right) = 0, \qquad with \quad \tilde{\lambda}_B := \epsilon \lambda_B \quad and \quad \tilde{\lambda}_C := \epsilon \lambda_C. \tag{9}$$

Proof. Under Assumptions 1 and 2, the partition S_r of the controller stoichiometry matrix S_c given in (2) and its associated propensity function $\lambda_r(z)$ can be further partitioned to

$$S_r := \left[\begin{array}{c|cc|cc|c|c} \overbrace{\mathcal{R}_{Q_1} \cdots \mathcal{R}_{Q_m}}^{\mathcal{R}_Q} & \multicolumn{2}{c|}{\overbrace{\mathcal{R}_{C_1}^+ \cdots \mathcal{R}_{C_{p_0}}^+ \; \mathcal{R}_{C_1}^- \cdots \mathcal{R}_{C_{p_0}}^-}^{\mathcal{R}_C}} & \multicolumn{2}{c|}{\overbrace{\mathcal{R}_{B_1}^+ \cdots \mathcal{R}_{B_p}^+ \; \mathcal{R}_{B_1}^- \cdots \mathcal{R}_{B_p}^-}^{\mathcal{R}_B}} & \overbrace{\mathcal{R}_{D_1} \cdots \mathcal{R}_{D_{m_0}}}^{\mathcal{R}_D} & \overbrace{\mathcal{R}_{\delta_1} \cdots \mathcal{R}_{\delta_M}}^{\mathcal{R}_\delta} \\ S_Q & S_C & -S_C & S_B & -S_B & S_D & -I_M \end{array}\right] \left.\begin{array}{c} \mathbf{Z}_1 \\ \mathbf{Z}_2 \\ \vdots \\ \vdots \\ \mathbf{Z}_M \end{array}\right\} \mathbf{Z}$$

$$\lambda_r(z) := \left[\begin{array}{c|cc|cc|c|c} \lambda_Q(z) & \lambda_C^+(z) & \lambda_C^-(z) & \lambda_B^+(z) & \lambda_B^-(z) & \lambda_D(z) & \lambda_\delta(z) \end{array}\right]^T,$$

where S_r is an $M \times (m + 2p_0 + 2p + m_0 + M)$ matrix and λ_r is an $(m + 2p_0 + 2p + m_0 + M) \times 1$ vector-valued function with

$$\begin{cases} \lambda_Q(z) = \eta v \circ (Q_1 z) \circ (Q_2 z) & \text{(Sequestration)} \\ \lambda_C(z) = \lambda_C^+(z) - \lambda_C^-(z) = c_1 \circ (C_1 z) - c_2 \circ (C_2 z) & \text{(Reversible Conversion)} \\ \lambda_B(z) = \lambda_B^+(z) - \lambda_B^-(z) = a \circ (B_1 z) \circ (B_2 z) - d \circ (B_3 z) & \text{(Reversible Binding)} \\ \lambda_D(z) = \delta_0 Q_0 z & \text{(Degradation)} \\ \lambda_\delta(z) = \delta z & \text{(Dilution).} \end{cases} \tag{10}$$

Proof of Embedded Generalized AIF Motif with Dilution: We first prove that the state transformation given in (7) yields a generalized embedded (non-ideal) antithetic integral feedback (AIF) motif with dilution. The ODEs governing the dynamics of z^+, z^- and z^0 can be calculated as follows

$$
\begin{aligned}
\frac{d}{dt}\begin{bmatrix} z^+ \\ z^- \\ z^0 \end{bmatrix} &= \begin{bmatrix} {q^+}^T \\ {q^-}^T \\ {q^0}^T \end{bmatrix} \frac{d}{dt} z = \begin{bmatrix} {q^+}^T \\ {q^-}^T \\ {q^0}^T \end{bmatrix} S_c \lambda_c(z, x_1, x_L) \\
&= \begin{bmatrix} {q^+}^T \\ {q^-}^T \\ {q^0}^T \end{bmatrix} [0 \; 0 \; I_M \; S_r] \begin{bmatrix} h^+ z(z, x_L) \\ h^-(z, x_L) x_1 \\ \mu + \theta x_L \\ \lambda_r(z) \end{bmatrix} = \begin{bmatrix} {q^+}^T \\ {q^-}^T \\ {q^0}^T \end{bmatrix} \Big(\mu + \theta x_L + S_r \lambda_r(z) \Big) \\
&= \begin{bmatrix} {q^+}^T \\ {q^-}^T \\ {q^0}^T \end{bmatrix} \Big(\mu + \theta x_L + S_Q \lambda_Q(z) + S_C \lambda_C(z) + S_B \lambda_B(z) + S_D \lambda_D(z) - \lambda_\delta(z) \Big) \\
&\implies \begin{cases} \dot{z}^+ = {q^+}^T (\mu + \theta x_L) + \eta {q^+}^T S_Q \left[v \circ (Q_1 z) \circ (Q_2 z) \right] - \delta {q^+}^T z \\ \dot{z}^- = {q^-}^T (\mu + \theta x_L) + \eta {q^-}^T S_Q \left[v \circ (Q_1 z) \circ (Q_2 z) \right] - \delta {q^-}^T z \\ \dot{z}^0 = {q^0}^T (\mu + \theta x_L) + \eta {q^0}^T S_Q \left[v \circ (Q_1 z) \circ (Q_2 z) \right] - \delta {q^0}^T z - \delta_0 {q^0}^T Q_0^T Q_0 z, \end{cases}
\end{aligned}
$$

which follows from the first condition in (6). We first analyze the quadratic term ${q^+}^T S_Q \left[v \circ (Q_1 z) \circ (Q_2 z) \right]$. Exploiting the third and fourth conditions in (6), we proceed as follows

$$
\begin{aligned}
{q^+}^T S_Q \left[v \circ (Q_1 z) \circ (Q_2 z) \right] &= {q^+}^T S_Q \left[(\mathcal{D}\{v\} Q_1 z) \circ (Q_2 z) \right] \\
&= z^T Q_1^T \mathcal{D}\{v\} \mathcal{D}\{S_Q^T q^+\} Q_2 z \\
&= -z^T Q_1^T \mathcal{D}\{\max\left(Q_1 q^+, Q_2 q^-\right)\} \mathcal{D}\left\{\min\left(Q_1 q^+, Q_2 q^-\right)\right\} Q_2 z \\
&= -z^T Q_1^T \mathcal{D}\left\{\max\left(Q_1 q^+, Q_2 q^-\right) \circ \min\left(Q_1 q^+, Q_2 q^-\right)\right\} Q_2 z \\
&= -z^T Q_1^T \mathcal{D}\left\{\left(Q_1 q^+\right) \circ \left(Q_2 q^-\right)\right\} Q_2 z \\
&= -z^T Q_1^T \mathcal{D}\left\{Q_1 q^+\right\} \mathcal{D}\left\{Q_2 q^-\right\} Q_2 z \\
&= -\left[\left(Q_1 q^+\right) \circ (Q_1 z)\right]^T \left[\left(Q_2 q^-\right) \circ (Q_2 z)\right],
\end{aligned}
$$

where the algebraic identities of the Hadamard product in Section 1 are exploited. Observe that the term $(Q_1 q^+) \circ (Q_1 z)$ can be simplified by partitioning $Q_1 = \left[Q_1^1 \cdots Q_1^M\right]$ and $q^+ = \left[q_1^+ \cdots q_M^+\right]^T$ where Q_1^i are $m \times 1$ vectors while q_i^+ are scalars for $i = 1, \cdots, M$ and by proceeding as follows

$$
\begin{aligned}
\left(Q_1 q^+\right) \circ (Q_1 z) &= \left(\sum_{i=1}^{M} q_i^+ Q_1^i \right) \circ \left(\sum_{j=1}^{M} z_j Q_1^j \right) \\
&= \sum_{i,j=1}^{M} \left(Q_1^i \circ Q_1^j \right) q_i^+ z_j \\
&= \sum_{i=1}^{M} \left(Q_1^i \circ Q_1^i \right) q_i^+ z_i \\
&= \sum_{i=1}^{M} Q_1^i q_i^+ z_i \\
&= Q_1 \left[q^+ \circ z \right],
\end{aligned}
$$

where the third and fourth equalities follow from the fact that Q_1 is a selector matrix (i.e. $Q_1^i \circ Q_1^j = 0$ for $i \neq j$ and $Q_1^i \circ Q_1^i = Q_1^i$). Similarly, we have

$$
\left(Q_2 q^-\right) \circ (Q_2 z) = Q_2 \left[q^- \circ z \right].
$$

Substituting for $(Q_1 q^+)\circ(Q_1 z)$ and $(Q_2 q^-)\circ(Q_2 z)$ in the quadratic term ${q^+}^T S_Q\left[v\circ(Q_1 z)\circ(Q_2 z)\right]$ yields

$$\begin{aligned}
{q^+}^T S_Q\left[v\circ(Q_1 z)\circ(Q_2 z)\right] &= -\left[(Q_1 q^+)\circ(Q_1 z)\right]^T\left[(Q_2 q^-)\circ(Q_2 z)\right]\\
&= -\left[q^+\circ z\right]^T Q_1^T Q_2\left[q^-\circ z\right]\\
&= -\left[q^+\circ z\right]^T \mathbb{1}\left(q^+{q^-}^T\right)\left[q^-\circ z\right]\\
&= -\left[q^+\circ z\right]^T\sum_{j=1}^{M}\mathbb{1}\left(q^+ q_j^-\right)q_j^- z_j\\
&= -\sum_{i=1}^{M} q_i^+ z_i\sum_{j=1}^{M}\mathbb{1}\left(q_i^+ q_j^-\right)q_j^- z_j\\
&= -\sum_{i,j=1}^{M}\mathbb{1}\left(q_i^+ q_j^-\right)q_i^+ q_j^- z_i z_j\\
&= -\sum_{i,j=1}^{M} q_i^+ q_j^- z_i z_j\\
&= -\sum_{i=1}^{M} q_i^+ z_i\sum_{j=1}^{M} q_j^+ z_j\\
&= -({q^+}^T z)({q^-}^T z)\\
&= -z^+ z^-,
\end{aligned}$$

where the third equality follows from the second condition in (6), and the seventh equality follows from the fact that q^+ and q^- are non-negative. Furthermore, invoking the third condition in (6), particularly $S_Q^T q^+ = S_Q^T q^- = -S_Q^T q^0$, yields the remaining quadratic terms, that is

$${q^+}^T S_Q\left[v\circ(Q_1 z)\circ(Q_2 z)\right] = {q^-}^T S_Q\left[v\circ(Q_1 z)\circ(Q_2 z)\right] = -{q^0}^T S_Q\left[v\circ(Q_1 z)\circ(Q_2 z)\right] = -z^+ z^-.$$

Finally, we analyze the remaining linear term ${q^0}^T Q_0^T Q_0 z$. Assumption 2 can be exploited here to get an expression for $Q_0^T Q_0$ in terms of Q_1 and Q_2. The $M\times M$ matrices $Q_0^T Q_0$, $Q_1^T Q_1$ and $Q_2^T Q_2$ are diagonal matrices because Q_0, Q_1 and Q_2 are selector matrices. The ith entry on the diagonal of $Q_0^T Q_0$ and $Q_1^T Q_1 + Q_2^T Q_2$ denote the number of reactions in which species $\mathbf{Z}_i$ participates in a degradation reaction and sequestration reaction, respectively. Since, $\mathbf{Z}_i$ degrades only if it does not participate in any sequestration reaction (Assumption 2), then

$$\begin{aligned}
Q_0^T Q_0 &= I_M - \mathbb{1}\left(Q_1^T Q_1 + Q_2^T Q_2\right)\\
&= I_M - \mathbb{1}\left(Q_1^T Q_1\right) - \mathbb{1}\left(Q_2^T Q_2\right)\\
&= I_M - \mathbb{1}\left(\mathcal{D}\left\{Q_1^T\mathbf{1}_m\right\}\right) - \mathbb{1}\left(\mathcal{D}\left\{Q_2^T\mathbf{1}_m\right\}\right)\\
&= I_M - \mathcal{D}\left\{\mathbb{1}\left(q^+\right)+\mathbb{1}\left(q^-\right)\right\},
\end{aligned}$$

where the second and third equalities follow by exploiting $Q_1 Q_2^T = 0$ (Assumption 2) in the selector matrix identities of Section 1, and the last equality follows from the second condition in (6). Hence we can proceed as follows

$$\begin{aligned}
{q^0}^T Q_0^T Q_0 z &= {q^0}^T\left[I_M - \mathcal{D}\left\{\mathbb{1}\left(q^+\right)+\mathbb{1}\left(q^-\right)\right\}\right]z\\
&= {q^0}^T z - {q^0}^T\mathcal{D}\left\{\mathbb{1}\left(q^+ + q^-\right)+\mathbb{1}\left(q^+\circ q^-\right)\right\}z\\
&= {q^0}^T z - {q^0}^T\mathcal{D}\left\{\mathbb{1}\left(q^+ + q^-\right)\right\}z\\
&= z^0 - \left[\mathbb{1}\left(q^+ + q^-\right)\circ q^0\right]^T z,
\end{aligned}$$

where the second equality follows from the indicator function identity in Section 1, and the third equality follows from the fact that $q^+\circ q^- = 0$ which can be seen by invoking the condition $\mathbb{1}\left(q^+{q^-}^T\right) = Q_1^T Q_2$ in (6). More precisely, we have

$$\begin{aligned}
\mathbb{1}\left(q^+\circ q^-\right) &= \mathcal{D}\left\{\mathbb{1}\left(q^+{q^-}^T\right)\right\}\\
&= \mathcal{D}\left\{Q_1^T Q_2\right\}^T\\
&= \mathbf{1}_m^T(Q_1\circ Q_2)\\
&= 0,
\end{aligned}$$

where the third equality follows from a Hadamard product identity in Section 1, and $Q_1 \circ Q_2 = 0$ follows from the fact the species cannot sequester themselves (Assumption 2). This completes the proof of the embedded (non-ideal) AIF motif with dilution described by the following ODEs

$$\begin{cases} \dot{z}^+ = {q^+}^T(\mu + \theta x_L) - \eta z^+ z^- - \delta z^+ \\ \dot{z}^- = {q^-}^T(\mu + \theta x_L) - \eta z^+ z^- - \delta z^- \\ \dot{z}^0 = {q^0}^T(\mu + \theta x_L) + \eta z^+ z^- - (\delta + \delta_0) z^0 + \delta_0 \left[\mathbb{1}\left(q^+ + q^-\right) \circ q^0\right]^T z. \end{cases} \tag{11}$$

Proof of Model Reduction: Next, we prove the validity of the reduced model based on singular perturbation theory [3] and the deficiency-zero theorem [4]. Since there exists at least one sequestration reaction that is assumed, without loss of generality, to occur between $\mathbf{Z_1}$ and $\mathbf{Z_2}$ to yield $\mathbf{Z_3}$ and possibly other products as well (Assumption 2), then there exists a subset of species indices $K \subset \{4, \cdots, M\}$ such that

$$\mathcal{R}_{Q_1}: \qquad \mathbf{Z_1} + \mathbf{Z_2} \xrightarrow{\eta} \mathbf{Z_3} + \sum_{k \in K} \mathbf{Z}_{\boldsymbol{k}}.$$

Based on this reaction, we will partition the vectors q^+, q^- and q^0. First we have that $q_1^+, q_2^- > 0$ and $q_2^+ = q_1^- = 0$. This can be seen by observing that

$$Q_1^T e_1 = e_1, \qquad Q_2^T e_1 = e_2, \qquad Q_3^T e_1 = e_3 + \sum_{k \in K} e_k \ \forall k \in K,$$

and invoking Condition 1, particularly $\mathbb{1}\left(q^+\right) = \mathbb{1}\left(Q_1^T \mathbf{1}_m\right)$ and $\mathbb{1}\left(q^-\right) = \mathbb{1}\left(Q_2^T \mathbf{1}_m\right)$ to obtain

$$\begin{aligned} \mathbb{1}\left(q_1^+\right) &= e_1^T \mathbb{1}\left(q^+\right) = e_1^T \mathbb{1}\left(Q_1^T \mathbf{1}_m\right) = \mathbb{1}\left(e_1^T Q_1^T \mathbf{1}_m\right) = \mathbb{1}\left(e_1^T Q_1^T e_1 + \sum_{k=2}^m e_1^T Q_1^T e_k\right) = \mathbb{1}\left(1 + \sum_{k=2}^m e_1^T Q_1^T e_k\right) = 1 \\ &\implies q_1^+ > 0 \\ \mathbb{1}\left(q_2^-\right) &= e_2^T \mathbb{1}\left(q^-\right) = e_2^T \mathbb{1}\left(Q_2^T \mathbf{1}_m\right) = \mathbb{1}\left(e_2^T Q_2 \mathbf{1}_m\right) = \mathbb{1}\left(e_2^T Q_2^T e_1 + \sum_{k=2}^m e_2^T Q_2^T e_k\right) = \mathbb{1}\left(1 + \sum_{k=2}^m e_2^T Q_2^T e_k\right) = 1 \\ &\implies q_2^- > 0, \end{aligned}$$

and $q_2^+ = q_1^- = 0$ directly follows from $q^+ \circ q^- = 0$. Furthermore, we have that $q^0 = \left[0 \ 0 \ {q^0}_3 \quad \star \quad \right]^T$ with $q_3^0 > 0$ by assumption then we can partition q^+, q^- and q^0 as follows

$$q_{\text{tot}}^T := \begin{bmatrix} q^+ & q^- & q^0 \end{bmatrix}^T = \left[\begin{array}{ccc|ccc} q_1^+ & 0 & q_3^+ & q_4^+ & \cdots & q_M^+ \\ 0 & q_2^- & q_3^- & q_4^- & \cdots & q_M^- \\ 0 & 0 & q_3^0 & q_4^0 & \cdots & q_M^0 \end{array}\right] =: \left[\begin{array}{c|c} \bar{q}^T & \tilde{q}^T \end{array}\right],$$

where $\bar{q} \in \mathbb{N}^{3 \times 3}$ is invertible and $\tilde{q} \in \mathbb{N}^{(M-3) \times 3}$. Let $z^{\text{tot}} := \left[z^+ \ z^- \ z^0\right]^T$ and $\tilde{z} := \left[z_4 \cdots z_M\right]^T =: \tilde{C} z$. Hence the state transformation in (7) can be rewritten using $\bar{q}$ and $\tilde{q}$ as

$$z^{\text{tot}} = q_{\text{tot}}^T z = \bar{q}^T \begin{bmatrix} z_1 \\ z_2 \\ z_3 \end{bmatrix} + \tilde{q}^T \tilde{z} \qquad \Longleftrightarrow \qquad \begin{bmatrix} z_1 \\ z_2 \\ z_3 \end{bmatrix} = \left(\bar{q}^T\right)^{-1} \left(z^{\text{tot}} - \tilde{q}^T \tilde{z}\right).$$

The full state transformation between z and $(z^{\text{tot}}, \tilde{z})$ is thus given by

$$\begin{bmatrix} z^{\text{tot}} \\ \tilde{z} \end{bmatrix} = \underbrace{\left[\begin{array}{c|c} \bar{q}^T & \tilde{q}^T \\ \hline 0_{(M-3)\times 3} & I_{M-3} \end{array}\right]}_{T^{-1}} z \Longleftrightarrow z = \underbrace{\left[\begin{array}{c|c} \left(\bar{q}^T\right)^{-1} & -\left(\bar{q}^T\right)^{-1} \tilde{q}^T \\ \hline 0_{(M-3)\times 3} & I_{M-3} \end{array}\right]}_{T} \begin{bmatrix} z^{\text{tot}} \\ \tilde{z} \end{bmatrix} = \bar{C}^T \left(\bar{q}^T\right)^{-1} z^{\text{tot}} + (\tilde{C}^T - \bar{C}^T \left(\bar{q}^T\right)^{-1} \tilde{q}^T) \tilde{z}$$

$$\text{with} \quad \tilde{C} := \left[0_{(M-3)\times 3} \ I_{M-3}\right], \qquad \bar{C} := \left[I_3 \ 0_{3 \times (M-3)}\right].$$

By letting $a = \tilde{a}/\epsilon, d = \tilde{d}/\epsilon, c_1 = \tilde{c}_1/\epsilon$ and $c_2 = \tilde{c}_2/\epsilon$ such that $\lambda_B =: \tilde{\lambda}_d/\epsilon$ and $\lambda_C =: \tilde{\lambda}_C/\epsilon$, the dynamics of $\tilde{z}$ can be written as

$$\begin{aligned}\dot{\tilde{z}} &= \tilde{C}\dot{z} = \tilde{C}\Big(\mu + \theta x_L + S_r\lambda_r(z)\Big) \\ &= \tilde{C}\Big(\mu + \theta x_L + S_Q\lambda_Q(z) + S_C\lambda_C(z) + S_B\lambda_B(z) + S_D\lambda_D(z) - \lambda_\delta(z)\Big) \\ &= \tilde{C}\Big(\mu + \theta x_L + S_Q\lambda_Q(z) + \frac{1}{\epsilon}S_C\tilde{\lambda}_C(z) + \frac{1}{\epsilon}S_B\tilde{\lambda}_B(z) + S_D\lambda_D(z) - \lambda_\delta(z)\Big) \\ \implies \epsilon\dot{\tilde{z}} &= \tilde{C}\Big(S_B\tilde{\lambda}_B(z) + S_C\tilde{\lambda}_C(z)\Big) + \epsilon\tilde{C}\Big(\mu + \theta x_L + S_Q\lambda_Q(z) + S_D\lambda_D(z) - \delta z\Big).\end{aligned}$$

By including the dynamics of z^{tot} and the regulated network, one can now write the full closed-loop dynamics in the standard singular perturbation form given by

$$\begin{cases} \dot{x} = f(x) + \left(u^+ - u^- x_1\right)e_1; \qquad u^\pm = h^\pm(z, x_L); \qquad \text{with} \qquad z = \bar{C}^T\left(\bar{q}^T\right)^{-1}z^{\text{tot}} + (\tilde{C}^T - \bar{C}^T\left(\bar{q}^T\right)^{-1}\tilde{q}^T)\tilde{z} \\ \dot{z}^{\text{tot}} = F(\tilde{z}, z^{\text{tot}}, x_L) := \begin{bmatrix} {q^+}^T(\mu + \theta x_L) - \eta z^+ z^- - \delta z^+ \\ {q^-}^T(\mu + \theta x_L) - \eta z^+ z^- - \delta z^- \\ {q^0}^T(\mu + \theta x_L) + \eta z^+ z^- - (\delta + \delta_0)z^0 \ + \delta_0\left[\mathbb{1}\left(q^+ + q^-\right)\circ q^0\right]^T z \end{bmatrix} \\ \epsilon\dot{\tilde{z}} = G(\tilde{z}, z^{\text{tot}}; \epsilon) := \tilde{C}\Big(S_B\tilde{\lambda}_B(z) + S_C\tilde{\lambda}_C(z)\Big) + \epsilon\tilde{C}\Big(\mu + \theta x_L + S_Q\lambda_Q(z) + S_D\lambda_D(z) - \delta z\Big), \end{cases}$$

where f encodes for the dynamics of the regulated network and $h^\pm$ are the production and removal actuation rates (see Assumption 1). Therefore the slow manifold is given by the following nonlinear algebraic equation

$$G\left(\tilde{z}, z^{\text{tot}}; 0\right) = 0 \qquad \iff \qquad \tilde{C}\Big(S_B\tilde{\lambda}_B(z) + S_C\tilde{\lambda}_C(z)\Big) = 0 \quad \text{where} \quad z = \bar{C}^T\left(\bar{q}^T\right)^{-1}z^{\text{tot}} + (\tilde{C}^T - \bar{C}^T\left(\bar{q}^T\right)^{-1}\tilde{q}^T)\tilde{z}.$$

This set of algebraic equations defines implicitly the relationship between the slow variables $z^{\text{tot}} = \left[z^+ \ z^- \ z^0\right]^T$ and the fast variables $\tilde{z}$. It remains to show that the slow manifold indeed exists and is locally stable. More precisely, for any "frozen" z^{tot} there exists a $\tilde{z} \geq 0$ such that $\tilde{z} = \mathcal{G}(z^{\text{tot}})$ satisfying $G(\tilde{z}, z^{\text{tot}}; 0) = 0$ is locally stable. This can be done by first writing the fast dynamics in the original z-space and then invoking the zero-deficiency theorem [4]. The fast dynamics are given by

$$\dot{\tilde{z}} = \tilde{C}\Big(S_B\tilde{\lambda}_B(z) + S_C\tilde{\lambda}_C(z)\Big) \quad \text{where} \quad z = \bar{C}^T\left(\bar{q}^T\right)^{-1}z^{\text{tot}} + (\tilde{C}^T - \bar{C}^T\left(\bar{q}^T\right)^{-1}\tilde{q}^T)\tilde{z}.$$

To write the fast dynamics in the original z-space, we treat z^+, z^- and z^0 as constants and proceed as follows

$$\begin{aligned}\dot{z} &= \frac{d}{dt}\left(\bar{C}^T\left(\bar{q}^T\right)^{-1}z^{\text{tot}} + \left(\tilde{C}^T - \bar{C}^T\left(\bar{q}^T\right)^{-1}\tilde{q}^T\right)\tilde{z}\right) \\ &= \left(\tilde{C}^T - \bar{C}^T\left(\bar{q}^T\right)^{-1}\tilde{q}^T\right)\dot{\tilde{z}} \\ &= \left(\tilde{C}^T - \bar{C}^T\left(\bar{q}^T\right)^{-1}\tilde{q}^T\right)\tilde{C}\Big(S_B\tilde{\lambda}_B(z) + S_C\tilde{\lambda}_C(z)\Big) \\ &= \left(\tilde{C}^T\tilde{C}S_B - \bar{C}^T\left(\bar{q}^T\right)^{-1}\tilde{q}^T\tilde{C}S_B\right)\tilde{\lambda}_B(z) + \left(\tilde{C}^T\tilde{C}S_C - \bar{C}^T\left(\bar{q}^T\right)^{-1}\tilde{q}^T\tilde{C}S_C\right)\tilde{\lambda}_C(z).\end{aligned}$$

But we know that $\left(\bar{q}^T\right)^{-1}\tilde{q}^T\tilde{C}S_B = -\bar{C}S_B$ and $\left(\bar{q}^T\right)^{-1}\tilde{q}^T\tilde{C}S_C = -\bar{C}S_C$ because the first condition ${q^\pm}^T S_B = {q^0}^T S_B = {q^\pm}^T S_C = {q^0}^T S_C = 0$ in Condition 1 can be rewritten as

$$\begin{aligned} &q_{\text{tot}}^T S_B = 0 \quad \implies \quad \left[\bar{q}^T|\tilde{q}^T\right]S_B = 0 \quad \implies \quad (\bar{q}^T\bar{C} + \tilde{q}^T\tilde{C})S_B = 0 \quad \implies \quad \left(\bar{q}^T\right)^{-1}\tilde{q}^T\tilde{C}S_B = -\bar{C}S_B \\ &q_{\text{tot}}^T S_C = 0 \quad \implies \quad \left[\bar{q}^T|\tilde{q}^T\right]S_C = 0 \quad \implies \quad (\bar{q}^T\bar{C} + \tilde{q}^T\tilde{C})S_C = 0 \quad \implies \quad \left(\bar{q}^T\right)^{-1}\tilde{q}^T\tilde{C}S_C = -\bar{C}S_C.\end{aligned}$$

Then we have

$$\begin{aligned}\dot{z} &= \left(\tilde{C}^T\tilde{C}S_B + \bar{C}^T\bar{C}S_B\right)\tilde{\lambda}_B(z) + \left(\tilde{C}^T\tilde{C}S_C + \bar{C}^T\bar{C}S_C\right)\tilde{\lambda}_C(z) \\ &= \left(\tilde{C}^T\tilde{C} + \bar{C}^T\bar{C}\right)\left(S_B\tilde{\lambda}_B(z) + S_C\tilde{\lambda}_C(z)\right) \\ \implies \dot{z} &= S_B\tilde{\lambda}_B(z) + S_C\tilde{\lambda}_C(z).\end{aligned}$$

This means that the fast dynamics are governed by the binding and conversion reactions only, with the scaled association, dissociation and conversion rates $\tilde{a}, \tilde{d}, \tilde{c}_1$ and $\tilde{c}_2$, respectively, that is

$$\textbf{Binding-Conversion Network:} \begin{cases} B_1\mathbf{Z} + B_2\mathbf{Z} \underset{\tilde{d}}{\overset{\tilde{a}}{\rightleftharpoons}} B_3\mathbf{Z} \\ C_1\mathbf{Z} \underset{\tilde{c}_2}{\overset{\tilde{c}_1}{\rightleftharpoons}} C_2\mathbf{Z}. \end{cases}$$

By applying Lemma 1, we can conclude that since S_B is full-column rank and S_C is not a linear combination of S_B then the binding-conversion network has a zero deficiency. It then follows from the deficiency zero theorem [4] that the binding-conversion network is locally asymptotically stable with a single equilibrium within each positive compatibility class, that is $\forall z_0 \in \mathbb{R}^M_{>0}$ we have that $\dot{z} = S_B\tilde{\lambda}_B(z) + S_C\tilde{\lambda}_C(z)$ has a single fixed point and it is locally stable within $\left(z_0 + \mathrm{Im}\left(\left[S_B\ S_C\right]\right)\right) \cap \mathbb{R}^M_{>0}$.

To establish that the relationship $\tilde{z} = \mathcal{G}(z^{\text{tot}}) > 0$ is unique and is locally asymptotically stable within each compatibility class, we need to translate the result from z to $\tilde{z}$. This translation is established next by showing that the mapping between z and $\tilde{z}$ for any fixed z^{tot} is a bijection within each compatibility class. Define the following stoichiometry matrix and propensity function

$$S_{BC} := \left[S_B\ S_C\right] \qquad \text{and} \qquad \tilde{\lambda}_{BC}(z) := \begin{bmatrix} \tilde{\lambda}_B(z) \\ \tilde{\lambda}_C(z) \end{bmatrix}$$

to rewrite the two dynamical systems as

$$\mathcal{S} : \begin{cases} \dot{z} = S_{BC}\tilde{\lambda}_{BC}(z); \quad z(0) = z_0 \\ q_{\text{tot}}^T z = q_{\text{tot}}^T z_0 = z^{\text{tot}} \end{cases} \quad \xrightarrow{\ \tilde{z} = \tilde{C}z\ } \quad \tilde{\mathcal{S}} : \dot{\tilde{z}} = \tilde{C}S_{BC}\tilde{\lambda}_{BC}(T_1 z^{\text{tot}} + T_2\tilde{z}); \quad \tilde{z}(0) = \tilde{z}_0, \qquad \tilde{\mathcal{S}} \xrightarrow{\ z = T_1 z^{\text{tot}} + T_2\tilde{z}\ } \mathcal{S} \tag{12}$$

where T_1 and T_2 are the partitions of the transformation matrix T defined as

$$T = \left[\begin{array}{c|c} \left(\bar{q}^T\right)^{-1} & -\left(\bar{q}^T\right)^{-1}\tilde{q}^T \\ 0_{(M-3)\times 3} & I_{M-3} \end{array}\right] =: \left[\begin{array}{c|c} T_1 & T_2 \end{array}\right].$$

Note that $q_{\text{tot}}^T z = q_{\text{tot}}^T z_0 = z^{\text{tot}}$ represents the conservation laws for $\mathcal{S}$ which follows immediately from the condition that $q_{\text{tot}}^T S_{BC} = 0$ (condition 1). In fact, there are no other conservation laws since $\dim\left\{\ker\left(S_{BC}^T\right)\right\} = 3 = \mathrm{rank}(q_{\text{tot}})$ which immediately follows from the theorem condition that $\mathrm{rank}(S_{BC}) = p + \mathrm{rank}(S_C) = M - 3$. Define the following mapping

$$\begin{array}{cccl} \mathcal{F}_{z^{\text{tot}}} : & \mathbb{R}^{M-3}_{>0} & \longmapsto & \mathcal{Z} = \left\{z \in \mathbb{R}^M_{>0} : q_{\text{tot}}^T z = q_{\text{tot}}^T z_0 =: z^{\text{tot}}\right\} \\ & \tilde{z} & \longmapsto & z = \mathcal{F}_{z^{\text{tot}}}(\tilde{z}) := T_1 z^{\text{tot}} + T_2\tilde{z}. \end{array}$$

Note that $\mathcal{Z}$ is the compatibility class of $\mathcal{S}$ associated with the initial condition z_0 because

$$\begin{aligned} \left(z_0 + \mathrm{Im}\left(S_{BC}\right)\right) \cap \mathbb{R}^M_{>0} &= \left(z_0 + \mathrm{Ker}\left(S_{BC}^T\right)^{\perp}\right) \cap \mathbb{R}^M_{>0} \\ &= \left(z_0 + \mathrm{Im}\left(q_{\text{tot}}\right)^{\perp}\right) \cap \mathbb{R}^M_{>0} \\ &= \left(z_0 + \mathrm{Ker}\left(q_{\text{tot}}^T\right)\right) \cap \mathbb{R}^M_{>0} \\ &= \left\{z = z_0 + y \in \mathbb{R}^M : q_{\text{tot}}^T y = 0\right\} \cap \mathbb{R}^M_{>0} \\ &= \left\{z \in \mathbb{R}^M : q_{\text{tot}}^T (z - z_0) = 0\right\} \cap \mathbb{R}^M_{>0} \\ &= \left\{z \in \mathbb{R}^M_{>0} : q_{\text{tot}}^T z = q_{\text{tot}}^T z_0 =: z^{\text{tot}}\right\} = \mathcal{Z}, \end{aligned}$$

where the second equality follows from the fact that $q_{\text{tot}}^T z = z^{\text{tot}}$ are the only conservation laws of $\mathcal{S}$. The goal here is to prove that $\mathcal{F}_{z^{\text{tot}}}$ is a bijection. First, we prove by contradiction that it is an injection. Let $\tilde{z}_1 \neq \tilde{z}_2 \in \mathbb{R}^{M-3}_{>0}$ such that $\mathcal{F}_{z^{\text{tot}}}(\tilde{z}_1) = \mathcal{F}_{z^{\text{tot}}}(\tilde{z}_2)$. This implies that

$$T_1 z^{\text{tot}} + T_2\tilde{z}_1 = T_1 z^{\text{tot}} + T_2\tilde{z}_2 \qquad \Longrightarrow \qquad T_2(\tilde{z}_1 - \tilde{z}_2) \qquad \Longrightarrow \tilde{z}_1 = \tilde{z}_2,$$

which follows from the fact that T_2 is full column rank and thus $\mathrm{Ker}(T_2) = \{0\}$. This equality gives a contradiction proving that $\mathcal{F}_{z^{\text{tot}}}$ is an injection. Next, we prove that it is a surjection within each compatibility class. Let $z \in \mathcal{Z}$ and define $\tilde{z} = \tilde{C}z$, then we have

$$\begin{aligned} \mathcal{F}_{z^{\text{tot}}}(\tilde{z}) &= \mathcal{F}_{z^{\text{tot}}}(\tilde{C}z) \\ &= T_1 z^{\text{tot}} + T_2\tilde{C}z \\ &= (T_1 q_{\text{tot}}^T + T_2\tilde{C})z \\ &= \left[T_1\ T_2\right]\begin{bmatrix} q_{\text{tot}}^T \\ \tilde{C} \end{bmatrix} z \\ &= TT^{-1}z = z, \end{aligned}$$

and thus establishing that $\mathcal{F}_{z^{\text{tot}}}$ is a surjection within each compatibility class. This is essentially the outcome of having $q_{\text{tot}}^T z$ = constant as the only conservation laws in the binding-conversion network which is guaranteed if $\text{rank}(S_{BC}) = M - 3$. Therefore $\mathcal{F}_{z^{\text{tot}}}$ is a bijection within each compatibility class. This translates the local stability of the unique equilibrium within each compatibility class from the system $\mathcal{S}$ to $\tilde{\mathcal{S}}$ and thus establishes that the slow manifold exists and is locally stable. The final form of the reduced model can thus be calculated by first recalling $\bar{q} = \bar{C}q_{\text{tot}}$ and $\tilde{q} = \tilde{C}q_{\text{tot}}$ to rewrite z as

$$z = \bar{C}^T \left(\bar{q}^T\right)^{-1} z^{\text{tot}} + (\tilde{C}^T - \bar{C}^T \left(\bar{q}^T\right)^{-1} \tilde{q}^T)\tilde{z} = \underbrace{\bar{C}^T \left(\bar{C}q_{\text{tot}}\right)^{-T}}_{W_1^T} z^{\text{tot}} + \underbrace{(I_M - \bar{C}^T \left(\bar{C}q_{\text{tot}}\right)^{-T} q_{\text{tot}}^T)\tilde{C}^T}_{W_2^T} \tilde{z},$$

where $\tilde{z} = \mathcal{G}(z^{\text{tot}})$ is calculated from the slow manifold equation by solving the following nonlinear algebraic equations

$$\tilde{C}S_B\tilde{\lambda}_B\left(W_1^T z^{\text{tot}} + W_2^T \tilde{z}\right) + \tilde{C}S_C\tilde{\lambda}_C\left(W_1^T z^{\text{tot}} + W_2^T \tilde{z}\right) = 0,$$

and the control action $u^{\pm}$ is given by (see Assumption 1)

$$u^{\pm} = h^{\pm}(z, x_L) = h^{\pm}\left(\psi(z^{\text{tot}}), x_L\right) =: \mathcal{U}^{\pm}(z^{\text{tot}}, x_L),$$

where $\psi(z^{\text{tot}}) := W_1^T z^{\text{tot}} + W_2^T \tilde{z}$. Finally, the linear term $\delta_0 \left(\mathbb{1}\left(q^+ + q^-\right) \circ q^0\right)^T z$ in (11) is calculated on the slow manifold, $\tilde{z} = \mathcal{G}(z^{\text{tot}})$, as

$$\delta_0 \left(\mathbb{1}\left(q^+ + q^-\right) \circ q^0\right)^T \psi(z^{\text{tot}}),$$

which completes the proof of the model reduction. □

Next, we prove Theorem 2 (of the main text) by applying Supplementary Theorem 2 to the closed-loop network of Fig. 2 in the main text where the controller is particularly intein-based.

Proof of Theorem 1 of the main text.

Proof. To apply Supplementary Theorem 2, we first demonstrate that Conditions 1 are satisfied. Let q_i^+, q_i^- and q_i^- respectively denote the number of active IntC, active IntN and monomers with no active intein segments present in controller species $\mathbf{Z}_i$ for $i = 1, \cdots M$, and define

$$\begin{aligned} q^+ &:= \begin{bmatrix} q_1^+ & \cdots & q_M^+ \end{bmatrix} \\ q^- &:= \begin{bmatrix} q_1^- & \cdots & q_M^- \end{bmatrix} \\ q^0 &:= \begin{bmatrix} q_1^0 & \cdots & q_M^0 \end{bmatrix}. \end{aligned}$$

By adopting the compact notation for multiple reactions presented in Section 1, we now update Supplementary Table 2 with the additional reaction rules (9-13) to obtain Supplementary Table 3. Observe that these reactions satisfy Assumptions 1 and 2.

**Rule #**	**Reactions**	**Stoichiometry Matrix**	**Propensity Function**
Rule 1	$\varnothing \xrightarrow{\mu + \theta x_L} \mathbf{Z}$	I_M	$\mu + \theta x_L$
Rule 2	$Q_1\,\mathbf{Z} + Q_2\,\mathbf{Z} \xrightarrow{\bar{\eta}} Q_3\,\mathbf{Z}$	$S_Q := (Q_3 - Q_2 - Q_1)^T$	$\bar{\eta} \circ (Q_1 z) \circ (Q_2 z)$
Rule 3	$\varnothing \xrightarrow{h^+(z,x_L)} \mathbf{X_1} \xrightarrow{h^-(z,x_L)} \varnothing$	$\pm\begin{bmatrix}1 & 0 & \cdots & 0\end{bmatrix}^T$	$h^+(z, x_L), \quad h^-(z, x_L)x_1$
Rule 4	$C_1\,\mathbf{Z} \underset{c_2}{\overset{c_1}{\rightleftharpoons}} C_2\,\mathbf{Z}$	$\pm S_C := (C_2 - C_1)^T$	$c_1 \circ (C_1 z) - c_2 \circ (C_2 z)$
Rules 5,6,7	$B_1\,\mathbf{Z} + B_2\,\mathbf{Z} \underset{d}{\overset{a}{\rightleftharpoons}} B_3\,\mathbf{Z}$	$\pm S_B := \pm(B_3 - B_2 - B_1)^T$	$a \circ (B_1 z) \circ (B_2 z), \quad d \circ (B_3 z)$
Rule 11	$Q_0\,\mathbf{Z} \xrightarrow{\delta_0} \varnothing$	$S_D := -Q_0^T$	$\delta_0 Q_0 z$
Rule 12	$\mathbf{Z} \xrightarrow{\delta} \varnothing$	$-I_M$	δz

Supplementary Table 3: List of allowed controller reactions that respect Species Rules 1-3 and Reaction Rules 1-7,9-13 in the main text.

Note that "$\circ$" denotes the Hadamard (element-wise) product between two vectors. Furthermore, the rate parameters $\mu, \theta, \bar{\eta}, c_1, c_2, a, d, \delta_0, \delta$ are all nonnegative vectors and $Q_0, Q_1, Q_2, C_1, C_2, B_1, B_2, B_3$ are all selector matrices (see Section 1). According to Reaction Rule 2 in Fig. 2 of the main text, the selector matrices Q_1 and Q_2 select controller

species from the $\mathcal{C}$- and $\mathcal{N}$-classes, respectively; whereas Q_3 is allowed to select multiple controller species one of which belongs to the $\mathcal{S}$-class. Mathematically this implies that

$$\mathbb{1}\left(q^{+}\right) = \mathbb{1}\left(Q_1^T \mathbf{1}_m\right) \quad \text{and} \quad \mathbb{1}\left(q^{-}\right) = \mathbb{1}\left(Q_2^T \mathbf{1}_m\right),$$

with m being the number of intein-splicing (sequestration) reactions where active IntN and IntC segments are stoichiometrically inactivated. Mathematically, this means that

$$q^{+T} S_Q = q^{-T} S_Q = -q^{0T} S_Q = -\min\left(q^{+T} Q_1^T, q^{-T} Q_2^T\right),$$

because each active IntN segment stoichiometrically heterodimerizes with an active IntC segment to produce an inactive IntN-IntC segment. Furthermore, according to Reaction Rule 9 in the main text, all molecules with an active IntC segment can undergo an intein-splicing reaction with all molecules with an active IntN segment. Mathematically, this means that

$$\mathbb{1}\left(q^{+} q^{-T}\right) = Q_1^T Q_2.$$

Next, according to Reaction Rules 4 and 10 in the main text, the selector matrices C_1 and C_2 select controller species from the same class. These reversible conversion reactions (Reaction Rules 4,10) and binding reactions (Reaction Rules 5,6,7,10) conserve the number active IntC, active IntN and inactive IntC-IntN. Mathematically this means that

$$q^{+T} S_C = q^{-T} S_c = q^{0T} S_C = 0 \qquad \text{and} \qquad q^{+T} S_B = q^{-T} S_B = q^{0T} S_B = 0.$$

Since only controller molecules in the $\mathcal{S}$-class are allowed to degrade (Reaction Rule 11 in the main text), then controller molecules that contain active intein segments cannot degrade. Mathematically, this means that

$$q^{+T} S_D = q^{-T} S_D = 0.$$

Finally, according to Reaction Rule 13 in the main text stating that the rate of the $\ell^{\text{th}}-$intein splicing reaction between species $\mathbf{Z}_i$ and $\mathbf{Z}_j$ is given by

$$\bar{\eta}_\ell = \eta \max\left(q_i^{+}, q_j^{-}\right) \qquad \Longrightarrow \qquad \bar{\eta} = \eta v, \quad \text{with} \quad v := \max\left(Q_1 q^{+}, Q_2 q^{-}\right),$$

we conclude that Conditions 1 are satisfied. Therefore, applying Supplementary Theorem 2 finishes the proof. □

3 Mathematical Analysis of the Various Genetically Engineered Controller Circuits

In this section, we carry out the detailed mathemtical analysis of the various intein-based controller circuits presented in the main text. Theorems 1 and 2 are invoked here to establish RPA and derive reduced models for all our genetically built controller circuits. Furthermore, simulations are carried out to verify that the reduced models indeed capture the dynamics accurately.

3.1 TetR Controller Circuit

3.1.1 Full Model Description

Let DBD, DD and AD denote the DNA binding domain, dimerization domain and activation domain, respectively. The various controller species and reactions are listed in Supplementary Tables 4 and 5, respectively.

Species	Symbol	Species	Symbol
DBD:DD:Int^{C}:AD	$\mathbf{Z_1}$	$\mathbf{Z_1}$:$\mathbf{Z_1}$	$\mathbf{Z_5}$
Int^{N}	$\mathbf{Z_2}$	DBD:DD:Int^{C}:Int^{N}	$\mathbf{Z_3}$
$\mathbf{Z_3}$:$\mathbf{Z_3}$	$\mathbf{Z_4}$	$\mathbf{Z_3}$:$\mathbf{Z_1}$	$\mathbf{Z_6}$

Supplementary Table 4: List of Biochemical Species

Reaction	Mechanism	Constants
Set-Point	$\varnothing \xrightarrow{\mu_1} \mathbf{Z_1}$	
Sensing	$\mathbf{X_L} \xrightarrow{\theta_2} \mathbf{X_L} + \mathbf{Z_2}$	
Binding 1	$\mathbf{Z_1} + \mathbf{Z_1} \underset{d_1}{\overset{a_1}{\rightleftharpoons}} \mathbf{Z_5}$	$\kappa_1 := \frac{d_1}{a_1}$
Binding 2	$\mathbf{Z_1} + \mathbf{Z_3} \underset{d_2}{\overset{a_2}{\rightleftharpoons}} \mathbf{Z_6}$	$\kappa_2 := \frac{d_2}{a_2}$
Binding 3	$\mathbf{Z_3} + \mathbf{Z_3} \underset{d_3}{\overset{a_3}{\rightleftharpoons}} \mathbf{Z_4}$	$\kappa_3 := \frac{d_3}{a_3}$
Intein-Splicing 1	$\mathbf{Z_1} + \mathbf{Z_2} \xrightarrow{\eta} \mathbf{Z_3}$	
Intein-Splicing 2	$\mathbf{Z_5} + \mathbf{Z_2} \xrightarrow{2\eta} \mathbf{Z_6}$	
Intein-Splicing 3	$\mathbf{Z_6} + \mathbf{Z_2} \xrightarrow{\eta} \mathbf{Z_4}$	
Degradation	$\mathbf{Z_i} \xrightarrow{\delta_0} \varnothing, \qquad i = 3, 4$	
Dilution	$\mathbf{Z_i} \xrightarrow{\delta} \varnothing, \qquad i = 1, \cdots, 6$	
Actuation	$\varnothing \xrightarrow{u} \mathbf{X_1}$	$u := \frac{kz_5 + k'z_6}{1 + z_4/\kappa_u}$

Supplementary Table 5: List of Biochemical Reactions

3.1.2 Model Reduction

In this section, we apply Theorem 2 to obtain a reduced model for the detailed controller described in Supplementary Tables 4 and 5. The full controller model is schematically presented in Fig. 3 where $\mathbf{Z_5}$ and $\mathbf{Z_6}$ act as activators while $\mathbf{Z_4}$ act as a repressor as indicated in the actuation function $u = h^+(z) = \frac{kz_5 + k'z_6}{1 + z_4/\kappa_u}$. Under the assumption that the dimerization reactions are fast (i.e. $a_i = \tilde{a}_i/\epsilon, d_i = \tilde{d}_i/\epsilon$, for $i = 1, 2, 3$, as $\epsilon \to 0$), one can easily check that all the conditions of Theorem 2 are satisfied using the charge vectors q^+, q^- and q^0 given in Fig. 3. Applying Theorem 2

yields the following state variables

$$\begin{cases} z^+ = z_1 + 2z_5 + z_6 \\ z^- = z_2 \\ z^0 = z_3 + 2z_4 + z_6, \end{cases}$$

to arrive at the reduced model given by

$$\begin{cases} \dot{z}^+ = \mu_1 - \eta z^+ z^- - \delta z^+ \\ \dot{z}^- = \theta_2 x_L - \eta z^+ z^- - \delta z^- \\ \dot{z}^0 = \eta z^+ z^- - (\delta + \delta_0) z^0 + \delta_0 \tilde{z}_3, \end{cases}$$

where $\tilde{z} = \begin{bmatrix} \tilde{z}_1 & \tilde{z}_2 & \tilde{z}_3 \end{bmatrix}^T$ is given implicitly by the following algebraic equations

$$\tilde{C} S_B \tilde{\lambda}_B \left(W_1^T z^{\text{tot}} + W_2^T \tilde{z} \right) = 0 \implies \begin{cases} \tilde{a}_3 \left(z^0 - 2\tilde{z}_1 - \tilde{z}_3 \right)^2 - \tilde{d}_3 \tilde{z}_1 = 0 \\ \tilde{a}_1 \left(z^+ - 2\tilde{z}_2 - \tilde{z}_3 \right)^2 - \tilde{d}_1 \tilde{z}_2 = 0 \\ \tilde{a}_2 \left(z^0 - 2\tilde{z}_1 - \tilde{z}_3 \right) \left(z^+ - 2\tilde{z}_2 - \tilde{z}_3 \right) - \tilde{d}_2 \tilde{z}_3 = 0 \end{cases}$$

$$\implies \begin{cases} \left(z^0 - 2\tilde{z}_1 - \tilde{z}_3 \right)^2 = \kappa_3 \tilde{z}_1 \\ \left(z^+ - 2\tilde{z}_2 - \tilde{z}_3 \right)^2 = \kappa_1 \tilde{z}_2 \\ \kappa_1 \kappa_3 \tilde{z}_1 \tilde{z}_2 = \kappa_2^2 \tilde{z}_3^2. \end{cases} \tag{13}$$

Finally, the control action u is calculated as

$$u = h^+ \left(W_1^T z^{\text{tot}} + W_2^T \tilde{z} \right) = h^+ \left(\begin{bmatrix} z^+ - 2\tilde{z}_2 - \tilde{z}_3 \\ z^- \\ z^0 - 2\tilde{z}_1 - \tilde{z}_3 \\ \tilde{z}_1 \\ \tilde{z}_2 \\ \tilde{z}_3 \end{bmatrix}, x_L \right) = \frac{k\tilde{z}_2 + k'\tilde{z}_3}{1 + \frac{\tilde{z}_1}{\kappa_u}},$$

As a result, one can think of the control action u as a function $\mathcal{U}(z^+, z^0)$ defined implicitly as

$$u = \mathcal{U}(z^+, z^0) = \frac{k\tilde{z}_2 + k'\tilde{z}_3}{1 + \frac{\tilde{z}_1}{\kappa_u}},$$

where $\tilde{z}_1, \tilde{z}_2$ and $\tilde{z}_3$ solve the algebraic equations given implicitly in (13).

To summarize the result, the reduced model of the controller is described by the following *Differential Algebraic Equations* (DAEs).

$$\begin{aligned} &\text{Differential Equations:} && \begin{cases} \dot{z}^+ = \mu_1 - \eta z^+ z^- - \delta z^+ \\ \dot{z}^- = \theta_2 x_L - \eta z^+ z^- - \delta z^- \\ \dot{z}^0 = \eta z^+ z^- - (\delta + \delta_0) z^0 + \delta_0 \tilde{z}_3 \end{cases} \\ &\text{Control Action:} && u = \mathcal{U}(z^+, z^0) = \frac{k\tilde{z}_2 + k'\tilde{z}_3}{1 + \frac{\tilde{z}_1}{\kappa_u}} \\ &\text{Algebraic Equations:} && \begin{cases} \left(z^0 - 2\tilde{z}_1 - \tilde{z}_3 \right)^2 = \kappa_3 \tilde{z}_1 \\ \left(z^+ - 2\tilde{z}_2 - \tilde{z}_3 \right)^2 = \kappa_1 \tilde{z}_2 \\ \kappa_1 \kappa_3 \tilde{z}_1 \tilde{z}_2 = \kappa_2^2 \tilde{z}_3^2. \end{cases} \end{aligned} \tag{14}$$

Remark 1. *The result obtained in Fig. 6 is for the special case of $\delta_0 = 0$ which assumes that protein degradation is negligible compared to dilution.*

Remark 2. *The Differential Algebraic Equations (DAEs) describing the reduced model can be easily solved numerically. In fact, the algebraic equations can be recast as a single fourth order polynomial in $y_1 := \sqrt{\tilde{z}_1}$ as*

$$2\left[4\kappa_2^2 - \kappa_1\kappa_3\right] y_1^4 + \sqrt{\kappa_3}\left[8\kappa_2^2 - \kappa_1(\kappa_3 + 2\kappa_2)\right] y_1^3 + \left[2\kappa_2^2(\kappa_3 - 4z^0) - \kappa_1\kappa_3(\kappa_2 + z^+ - z^0)\right] y_1^2 + \cdots$$
$$\cdots + \kappa_2\sqrt{\kappa_3} z^0 \left[\kappa_1 - 4\kappa_2\right] y_1 + 2\left(\kappa_2 z^0\right)^2 = 0,$$

which can be numerically solved in MATLAB using the command "roots" within each iteration of an ODE solver without having to provide an initial guess. This gives us $\tilde{z}_1$. The other two variables $\tilde{z}_2$ and $\tilde{z}_3$ can thus be calculated as

$$\tilde{z}_2 = \frac{\kappa_2^2\left(\sqrt{\kappa_3\tilde{z}_1} - z^0 + 2\tilde{z}_1\right)^2}{\kappa_1\kappa_3\tilde{z}_1} \quad \textit{and} \quad \tilde{z}_3 = \frac{\sqrt{\kappa_1\kappa_3\tilde{z}_1\tilde{z}_2}}{\kappa_2}.$$

3.1.3 Monotonicity of Actuation

Next, we study the monotonicity of the control action u with respect to z^+ and z^0 , that is the monotonicity of the actuation function $\mathcal{U}$. For convenience, we rewrite the three algebraic equations in (14) as

$$\begin{cases} 2\tilde{z}_1 + \tilde{z}_3 + \sqrt{\kappa_3\tilde{z}_1} = z^0 \\ 2\tilde{z}_2 + \tilde{z}_3 + \sqrt{\kappa_1\tilde{z}_2} = z^+ \\ -\kappa_2\tilde{z}_3 + \sqrt{\kappa_1\kappa_3\tilde{z}_1\tilde{z}_2} = 0. \end{cases}$$

By differentiating the three equations with respect to z^+, we obtain the following linear system of equations

$$\underbrace{\begin{bmatrix} 2 + \frac{1}{2}\sqrt{\frac{\kappa_3}{\tilde{z}_1}} & 0 & 1 \\ 0 & 2 + \frac{1}{2}\sqrt{\frac{\kappa_1}{\tilde{z}_2}} & 1 \\ \frac{1}{2}\sqrt{\frac{\kappa_1\kappa_3\tilde{z}_2}{\tilde{z}_1}} & \frac{1}{2}\sqrt{\frac{\kappa_1\kappa_3\tilde{z}_1}{\tilde{z}_2}} & -\kappa_2 \end{bmatrix}}_{A} \begin{bmatrix} \frac{\partial\tilde{z}_1}{\partial z^+} \\ \frac{\partial\tilde{z}_2}{\partial z^+} \\ \frac{\partial\tilde{z}_3}{\partial z^+} \end{bmatrix} = \underbrace{\begin{bmatrix} 0 \\ 1 \\ 0 \end{bmatrix}}_{b^+}.$$

Then by Cramer's rule we obtain

$$\frac{\partial\tilde{z}_1}{\partial z^+} = \frac{\frac{1}{2}\sqrt{\frac{\kappa_1\kappa_3\tilde{z}_1}{\tilde{z}_2}}}{\det(A)}, \qquad \frac{\partial\tilde{z}_2}{\partial z^+} = -\frac{\kappa_2\left(2 + \frac{1}{2}\sqrt{\frac{\kappa_3}{\tilde{z}_1}}\right) + \frac{1}{2}\sqrt{\frac{\kappa_1\kappa_3\tilde{z}_2}{\tilde{z}_1}}}{\det(A)}, \qquad \frac{\partial\tilde{z}_3}{\partial z^+} = -\frac{\frac{1}{2}\sqrt{\frac{\kappa_1\kappa_3\tilde{z}_1}{\tilde{z}_2}}\left(2 + \frac{1}{2}\sqrt{\frac{\kappa_3}{\tilde{z}_1}}\right)}{\det(A)},$$

where

$$\det(A) = -\left(2 + \frac{1}{2}\sqrt{\frac{\kappa_3}{\tilde{z}_1}}\right)\left[\kappa_2\left(2 + \frac{1}{2}\sqrt{\frac{\kappa_1}{\tilde{z}_2}}\right) + \frac{1}{2}\sqrt{\frac{\kappa_1\kappa_3\tilde{z}_1}{\tilde{z}_2}}\right] - \frac{1}{2}\sqrt{\frac{\kappa_1\kappa_3\tilde{z}_2}{\tilde{z}_1}}\left(2 + \frac{1}{2}\sqrt{\frac{\kappa_1}{\tilde{z}_2}}\right) < 0.$$

Hence, we have

$$\frac{\partial\tilde{z}_1}{\partial z^+} < 0, \qquad \frac{\partial\tilde{z}_2}{\partial z^+} > 0, \qquad \text{and} \qquad \frac{\partial\tilde{z}_3}{\partial z^+} > 0 \qquad \Longrightarrow \frac{\partial\mathcal{U}(z^+, z^0)}{\partial z^+} > 0.$$

This means that the control action $u = \mathcal{U}(z^+, z^0)$ is a strictly monotonically increasing function of z^+.

Next, by differentiating the three equations with respect to z^0 , we obtain the following linear system of equations

$$\underbrace{\begin{bmatrix} 2 + \frac{1}{2}\sqrt{\frac{\kappa_3}{\tilde{z}_1}} & 0 & 1 \\ 0 & 2 + \frac{1}{2}\sqrt{\frac{\kappa_1}{\tilde{z}_2}} & 1 \\ \frac{1}{2}\sqrt{\frac{\kappa_1\kappa_3\tilde{z}_2}{\tilde{z}_1}} & \frac{1}{2}\sqrt{\frac{\kappa_1\kappa_3\tilde{z}_1}{\tilde{z}_2}} & -\kappa_2 \end{bmatrix}}_{A} \begin{bmatrix} \frac{\partial\tilde{z}_1}{\partial z^0} \\ \frac{\partial\tilde{z}_2}{\partial z^0} \\ \frac{\partial\tilde{z}_3}{\partial z^0} \end{bmatrix} = \underbrace{\begin{bmatrix} 1 \\ 0 \\ 0 \end{bmatrix}}_{b^0}.$$

Then by Cramer's rule we obtain

$$\frac{\partial\tilde{z}_1}{\partial z^0} = -\frac{\kappa_2\left(2 + \frac{1}{2}\sqrt{\frac{\kappa_1}{\tilde{z}_2}}\right) + \frac{1}{2}\sqrt{\frac{\kappa_1\kappa_3\tilde{z}_1}{\tilde{z}_2}}}{\det(A)}, \qquad \frac{\partial\tilde{z}_2}{\partial z^0} = \frac{\frac{1}{2}\sqrt{\frac{\kappa_1\kappa_3\tilde{z}_2}{\tilde{z}_1}}}{\det(A)}, \qquad \frac{\partial\tilde{z}_3}{\partial z^0} = -\frac{\frac{1}{2}\sqrt{\frac{\kappa_1\kappa_3\tilde{z}_2}{\tilde{z}_1}}\left(2 + \frac{1}{2}\sqrt{\frac{\kappa_1}{\tilde{z}_2}}\right)}{\det(A)}.$$

Hence, we have

$$\frac{\partial\tilde{z}_1}{\partial z^0} > 0, \qquad \frac{\partial\tilde{z}_2}{\partial z^0} < 0, \qquad \text{and} \qquad \frac{\partial\tilde{z}_3}{\partial z^0} > 0.$$

Since $\frac{\partial \tilde{z}_2}{\partial z^0}$ and $\frac{\partial \tilde{z}_3}{\partial z^0}$ have opposite signs, it is still unclear how the control action u varies with respect to z^0 . Hence to unravel the monotonicity of u with respect to z^0 , we proceed as follows

$$\frac{\partial \mathcal{U}(z^+, z^0)}{\partial z^0} = \frac{\left(k\frac{\partial \tilde{z}_2}{\partial z^0} + k'\frac{\partial \tilde{z}_3}{\partial z^0}\right)\left(1 + \frac{\tilde{z}_1}{\kappa_u}\right) - \frac{1}{\kappa_u}\frac{\partial \tilde{z}_1}{\partial z^0}(k\tilde{z}_2 + k'\tilde{z}_3)}{\left(1 + \frac{\tilde{z}_1}{\kappa_u}\right)^2}$$

$$= -\frac{\frac{1}{2}\sqrt{\frac{\kappa_1\kappa_3\tilde{z}_2}{\tilde{z}_1}}\left[k - k'\left(2 + \frac{1}{2}\sqrt{\frac{\kappa_1}{\tilde{z}_2}}\right)\right](1 + \frac{\tilde{z}_1}{\kappa_u}) + \frac{1}{\kappa_u}\left[\kappa_2\left(2 + \frac{1}{2}\sqrt{\frac{\kappa_1}{\tilde{z}_2}}\right) + \frac{1}{2}\sqrt{\frac{\kappa_1\kappa_3\tilde{z}_1}{\tilde{z}_2}}\right](k\tilde{z}_2 + k'\tilde{z}_3)}{|\det(A)|\left(1 + \frac{\tilde{z}_1}{\kappa_u}\right)^2}.$$

Observe that $\frac{\partial \mathcal{U}(z^+, z^0)}{\partial z^0}$ is not sign definite. The first term in the numerator is the only term that can make the expression positive; otherwise all the other terms are driving the expression to be negative. In fact, if $k' = 0$, the monotonicity of $\mathcal{U}$ is strictly decreasing in z^0 . To demonstrate this, let us examine two extreme cases. First, in the limit as $k \to \infty$ and finite κ_u, we have $\frac{\partial \mathcal{U}(z^+, z^0)}{\partial z^0} < 0$ for any $z^+, z^0 > 0$. Hence in this case, $\mathcal{U}$ is monotonically decreasing in z^0 . In the second case, in the limit as $\kappa_u \to \infty$ and $k < 2k'$, we have $\frac{\partial \mathcal{U}(z^+, z^0)}{\partial z^0} > 0$ for any $z^+, z^0 > 0$. Hence, in this case $\mathcal{U}$ is monotonically increasing in z^0 . These two extreme cases demonstrate that $\mathcal{U}$ is not sign definite. Furthermore, it is not difficult to see that for fixed parameter values, the function $\mathcal{U}$ switches monotonicity depending on the particular values of (z^+, z^0). In particular, when z^+ and z^0 are close to zero, $\tilde{z}_1, \tilde{z}_2$ and $\tilde{z}_3$ are also close to zero, and thus $\frac{\partial \mathcal{U}(z^+, z^0)}{\partial z^0}$ behaves like $\frac{k'}{\kappa_2}\sqrt{\kappa_1\tilde{z}_2} > 0$. As a result, if the dynamics are starting from a zero initial condition, u is initially an increasing function of z^0 for any choice of parameters. However, as the dynamics evolve and the levels of (z^+, z^0) rise sufficiently enough, the partial derivative $\frac{\partial \mathcal{U}(z^+, z^0)}{\partial z^0}$ switches its sign to become negative.

The switching of the monotonicity of u with respect to z^0 has a dynamically attractive feature. For low concentrations of controller species (reflecting low concentration level x_L of the regulated output), the control action u is an increasing function in both arguments (z^+, z^0). This makes the control action capable of positively actuating the controlled network quickly in the initial phase to rapidly increase the levels x_L of the output species. However, assuming that κ_u is sufficiently small (that is, the binding of the protein that is lacking an activation domain to the promoter is strong), as the dynamics evolve and the levels of z^+ and/or z^0 rise, the monotonicity switches sign so that the control action u becomes a decreasing function of z^0 , and thus adding a negative feedback to the standalone antithetic-integral motif. This additional negative feedback gives rise to a filtered proportional-integral control topology [5], but with a switching sign of the proportional gain. This is the reason behind switching behaviour, depicted in Fig. 6, of the underlying proportional component since the proportional gain K_P is defined as the partial derivative of the control action $\mathcal{U}$ with respect to z^0 . The switching threshold can be tuned by k' and κ_u. The aforementioned quantitative analysis is demonstrated numerically in Supplementary Fig. 3. The reduced model is depicted in Supplementary Fig. 3(a) and numerical simulations are shown in Supplementary Fig. 3(b) and (c) for the ideal scenario where the dilution rate is $\delta = 0$. The simulations demonstrate that increasing k' introduces a switch in monotonicity of the control action $\mathcal{U}$ in terms of z^0 (Panel c). This leads to to more aggressive control action at low levels of (z^+, z^0) and thus speeding up the output response as demonstrated in Panel b.

3.1.4 Simulation Results

To demonstrate the accuracy of the model reduction result via simulations, we use the two regulated networks depicted in Supplementary Fig. 4(a) as examples. Regulated Network 1 is comprised of two species $\mathbf{X_1}$ and $\mathbf{X_2}$ and may represent a simple model for gene expression; whereas Regulated Network 2 is more complex and is comprised of six species $\mathbf{X_1}$ through $\mathbf{X_6}$ (taken from [2, 6]). The simulation results for both regulated networks are depicted in Supplementary Fig. 4(c) demonstrating that the reduced model captures the dynamics of the full model to a high degree of accuracy as expected.

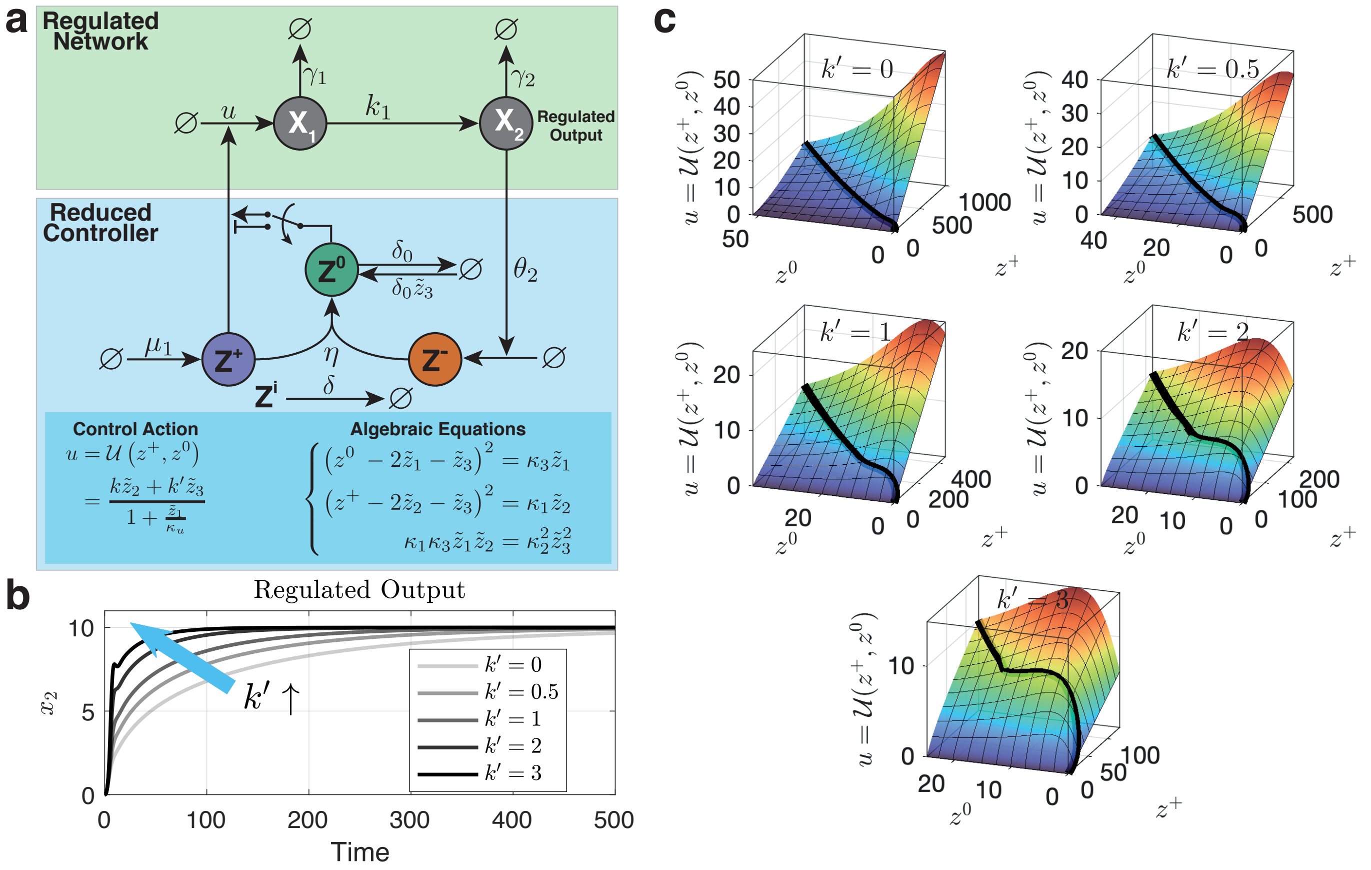

Supplementary Figure 3: Monotonicity of the control action of the TetR controller. (a) The reduced model of the TetR controller connected in a feedback interconnection with a regulated network comprised of two species $\mathbf{X_1}$ and $\mathbf{X_2}$ as an example, where $\mathbf{X_2}$ is the regulated output. (b) Simulation of the closed-loop dynamics for different values of k'. The concentration response of the regulated output is plotted for $k' \in \{0, 0.5, 1, 2, 3\}$. The parameters of the regulated network are intentionally picked to yield a slow response for $k' = 0$ to demonstrate that, in this case, k' can be conveniently increased to speed up the response. (c) The control map $u = \mathcal{U}(z^+, z^0)$ plotted as a function of z^+ and z^0 for the various values of k' used for the numerical simulations in panel b. The solid black line shows how the control action evolves for each of the associated simulations shown in panel b. For $k' = 0$, the control map is strictly monotonically increasing in z^+ and strictly monotonically decreasing in z^0. As k' is increased, a change in monotonicity of $\mathcal{U}$, as a function of z^0, is introduced such that at low levels of (z^+, z^0), the control map $\mathcal{U}$ is an increasing function of z^0. Observe that for all five values of k', the steady-state control action $\bar{u} = 10$ is the same. The difference between the control actions in the five cases is how the control action evolves transiently on the control map to reach the steady-state value. The increasing monotonicity of the control map for higher values of k' allows the control action to increase more aggressively towards the steady-state value. This leads to speeding up the regulated output response as demonstrated in panel b.
Numerical values. $k_1 = \gamma_1 = \gamma_2 = \theta_2 = \kappa_u = \delta_0 = 1, \kappa_1 = \kappa_2 = \kappa_3 = 1, \mu_1 = \eta = 10, k = 0.1, \delta = 0.$

3.2 Gal4 Controller Circuit

3.2.1 Full Model Description

Let DBD, DD and AD denote the DNA binding domain, dimerization domain and activation domain, respectively. The various controller species and reactions are listed in Supplementary Tables 6 and 7, respectively.

Species	Symbol	Species	Symbol
DBD:Int^{C}:DD:AD	$\mathbf{Z_1}$	$\mathbf{Z_1}$:$\mathbf{Z_1}$	$\mathbf{Z_5}$
Int^{N}	$\mathbf{Z_2}$	DD:AD	$\mathbf{Z_3}$
$\mathbf{Z_3}$:$\mathbf{Z_3}$	$\mathbf{Z_4}$	$\mathbf{Z_3}$:$\mathbf{Z_1}$	$\mathbf{Z_6}$

Supplementary Table 6: List of Biochemical Species

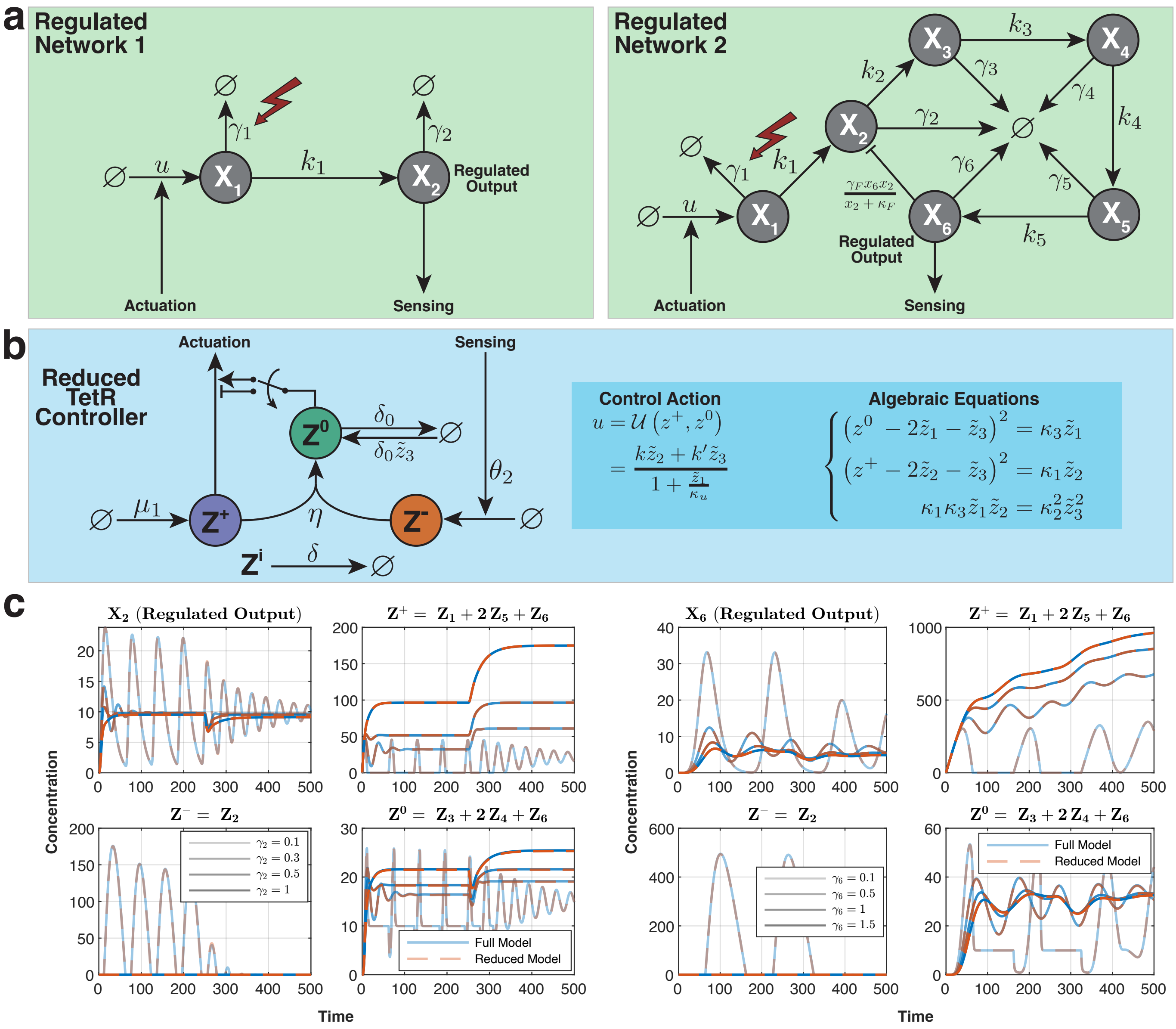

Supplementary Figure 4: Accuracy of the reduced model of the TetR controller. (a) Two test regulated networks. The first regulated network is comprised of two species $\mathbf{X_1}$ and $\mathbf{X_2}$, where $\mathbf{X_2}$ denotes the regulated output. The second regulated network is comprised of six species $\mathbf{X_1}$ through $\mathbf{X_6}$, where $\mathbf{X_6}$ denotes the regulated output. Both regulated networks are actuated by the control action u entering the dynamics as a production rate of the input species $\mathbf{X_1}$. (b) The reduced model of the TetR controller. The motif of the reduced model is comprised of the three effective species $\mathbf{Z^+}$, $\mathbf{Z^-}$ and $\mathbf{Z^0}$, as per Theorem 2, coupled to the three algebraic equations in $\tilde{z}_1, \tilde{z}_2$ and $\tilde{z}_3$. (c) Simulations demonstrating the accuracy of the reduced model of the controller. The simulations are carried out for the closed-loops where the TetR controller is connected in a feedback interconnection with each of the two regulated networks. The simulations are carried out starting from zero initial conditions at $t = 0$ and by doubling the degradation rate γ_1 of the input species γ_1 as a disturbance at $t = 250$. The simulations are also carried out for four different (initial) values of the degradation rates of the regulated outputs. The simulation results show that the reduced model is capable of accurately capturing the full dynamics of the full controller.
Numerical values. Regulated Network 1: $\gamma_1 = 0.1, k_1 = 1, k = 0.1, k' = 0.05, \kappa_u = \delta_0 = \theta_2 = 1, \delta = 0.005, \mu = \eta = 10, a_i = d_i = 100$ for $i = 1, 2, 3$.
Regulated Network 2: $\gamma_i = k_i = 0.1$ for $i = 1, \cdots, 5$, $\gamma_F = 0.3, \kappa_F = 1, k = 0.1, k' = 0.05, \kappa_u = \delta_0 = \theta_2 = 1, \delta = 0.005, \mu = \eta = 10, a_i = d_i = 100$ for $i = 1, 2, 3$.

3.2.2 Model Reduction

In this section, we apply Theorem 2 to obtain a reduced model for the detailed controller described in Supplementary Tables 6 and 7. The full controller model is schematically presented in Fig. 3 where $\mathbf{Z_5}$ acts as an activator as indicated in the actuation function $u = h(z) = kz_5$. Under the assumption that the dimerization reactions are fast (i.e. $a_i = \tilde{a}_i/\epsilon, d_i = \tilde{d}_i/\epsilon$, for $i = 1, 2, 3$, as $\epsilon \to 0$), one can easily check that all the assumptions and conditions of Theorem 2 are satisfied using the charge vectors q^+, q^- and q^0 depicted in Fig. 3. Applying Theorem 2 yields the

Reaction	Mechanism	Constants
Set-Point	$\varnothing \xrightarrow{\mu_1} \mathbf{Z_1}$	
Sensing	$\mathbf{X_L} \xrightarrow{\theta_2} \mathbf{X_L} + \mathbf{Z_2}$	
Binding 1	$\mathbf{Z_1} + \mathbf{Z_1} \underset{d_1}{\overset{a_1}{\rightleftharpoons}} \mathbf{Z_5}$	$\kappa_1 := \frac{d_1}{a_1}$
Binding 2	$\mathbf{Z_1} + \mathbf{Z_3} \underset{d_2}{\overset{a_2}{\rightleftharpoons}} \mathbf{Z_6}$	$\kappa_2 := \frac{d_2}{a_2}$
Binding 3	$\mathbf{Z_3} + \mathbf{Z_3} \underset{d_3}{\overset{a_3}{\rightleftharpoons}} \mathbf{Z_4}$	$\kappa_3 := \frac{d_3}{a_3}$
Intein-Splicing 1	$\mathbf{Z_1} + \mathbf{Z_2} \xrightarrow{\eta} \mathbf{Z_3}$	
Intein-Splicing 2	$\mathbf{Z_5} + \mathbf{Z_2} \xrightarrow{2\eta} \mathbf{Z_6}$	
Intein-Splicing 3	$\mathbf{Z_6} + \mathbf{Z_2} \xrightarrow{\eta} \mathbf{Z_4}$	
Degradation	$\mathbf{Z_i} \xrightarrow{\delta_0} \varnothing, \qquad i = 3, 4$	
Dilution	$\mathbf{Z_i} \xrightarrow{\delta} \varnothing, \qquad i = 1, \cdots, 6$	
Actuation	$\varnothing \xrightarrow{u} \mathbf{X_1}$	$u := kz_5$

Supplementary Table 7: List of Biochemical Reactions

following state variables

$$\begin{cases} z^+ = z_1 + 2z_5 + z_6 \\ z^- = z_2 \\ z^0 = z_3 + 2z_4 + z_6, \end{cases}$$

to arrive at the reduced model given by

$$\begin{cases} \dot{z}^+ = \mu_1 - \eta z^+ z^- - \delta z^+ \\ \dot{z}^- = \theta_2 x_L - \eta z^+ z^- - \delta z^- \\ \dot{z}^0 = \eta z^+ z^- - (\delta + \delta_0) z^0 + \delta_0 \tilde{z}_3, \end{cases}$$

where $\tilde{z} = \begin{bmatrix} \tilde{z}_1 & \tilde{z}_2 & \tilde{z}_3 \end{bmatrix}^T$ is given implicitly by the following algebraic equations

$$\tilde{C}S_B\tilde{\lambda}_B \left(W_1^T z^{\text{tot}} + W_2^T \tilde{z}\right) = 0 \implies \begin{cases} \tilde{a}_3 \left(z^0 - 2\tilde{z}_1 - \tilde{z}_3\right)^2 - \tilde{d}_3 \tilde{z}_1 = 0 \\ \tilde{a}_1 \left(z^+ - 2\tilde{z}_2 - \tilde{z}_3\right)^2 - \tilde{d}_1 \tilde{z}_2 = 0 \\ \tilde{a}_2 \left(z^0 - 2\tilde{z}_1 - \tilde{z}_3\right)\left(z^+ - 2\tilde{z}_2 - \tilde{z}_3\right) - \tilde{d}_2 \tilde{z}_3 = 0 \end{cases}$$
$$\implies \begin{cases} \left(z^0 - 2\tilde{z}_1 - \tilde{z}_3\right)^2 = \kappa_3 \tilde{z}_1 \\ \left(z^+ - 2\tilde{z}_2 - \tilde{z}_3\right)^2 = \kappa_1 \tilde{z}_2 \\ \kappa_1 \kappa_3 \tilde{z}_1 \tilde{z}_2 = \kappa_2^2 \tilde{z}_3^2. \end{cases} \tag{15}$$

Finally, the control action u is calculated as

$$u = h^+ \left(W_1^T z^{\text{tot}} + W_2^T \tilde{z}\right) = h^+ \left(\begin{bmatrix} z^+ - 2\tilde{z}_2 - \tilde{z}_3 \\ z^- \\ z^0 - 2\tilde{z}_1 - \tilde{z}_3 \\ \tilde{z}_1 \\ \tilde{z}_2 \\ \tilde{z}_3 \end{bmatrix}, x_L \right) = k\tilde{z}_2,$$

As a result, one can think of the control action u as a function $\mathcal{U}(z^+, z^0)$ defined implicitly as

$$u = \mathcal{U}(z^+, z^0) = k\tilde{z}_2,$$

where $\tilde{z}_1, \tilde{z}_2$ and $\tilde{z}_3$ solve the algebraic equations given implicitly in (15).

To summarize the result, the reduced model of the controller is described by the following *Differential Algebraic Equations* (DAEs).

$$
\begin{array}{ll}
\text{Differential Equations:} & \begin{cases} \dot{z}^+ = \mu_1 - \eta z^+ z^- - \delta z^+ \\ \dot{z}^- = \theta_2 x_L - \eta z^+ z^- - \delta z^- \\ \dot{z}^0 = \eta z^+ z^- - (\delta + \delta_0) z^0 + \delta_0 \tilde{z}_3 \end{cases} \\
\text{Control Action:} & u = \mathcal{U}(z^+, z^0) = k \tilde{z}_2 \\
\text{Algebraic Equations:} & \begin{cases} \left(z^0 - 2\tilde{z}_1 - \tilde{z}_3\right)^2 = \kappa_3 \tilde{z}_1 \\ \left(z^+ - 2\tilde{z}_2 - \tilde{z}_3\right)^2 = \kappa_1 \tilde{z}_2 \\ \kappa_1 \kappa_3 \tilde{z}_1 \tilde{z}_2 = \kappa_2^2 \tilde{z}_3^2. \end{cases}
\end{array}
\tag{16}
$$

Remark 3. *The result obtained in Fig. 6 is for the special case of $\delta_0 = 0$ which assumes that protein degradation is negligible compared to dilution.*

Remark 4. *The Differential Algebraic Equations (DAEs) describing the reduced model can be easily solved numerically. In fact, the algebraic equations can be recast as a single fourth order polynomial in $y_1 := \sqrt{\tilde{z}_1}$ as*

$$
2\left[4\kappa_2^2 - \kappa_1\kappa_3\right] y_1^4 + \sqrt{\kappa_3}\left[8\kappa_2^2 - \kappa_1(\kappa_3 + 2\kappa_2)\right] y_1^3 + \left[2\kappa_2^2(\kappa_3 - 4z^0) - \kappa_1\kappa_3(\kappa_2 + z^+ - z^0)\right] y_1^2 + \cdots
$$
$$
\cdots + \kappa_2\sqrt{\kappa_3} z^0 \left[\kappa_1 - 4\kappa_2\right] y_1 + 2\left(\kappa_2 z^0\right)^2 = 0,
$$

which can be numerically solved in MATLAB using the command "roots" within each iteration of an ODE solver without having to provide an initial guess. This gives us $\tilde{z}_1$. The other two variables $\tilde{z}_2$ and $\tilde{z}_3$ can thus be calculated as

$$
\tilde{z}_2 = \frac{\kappa_2^2\left(\sqrt{\kappa_3\tilde{z}_1} - z^0 + 2\tilde{z}_1\right)^2}{\kappa_1\kappa_3\tilde{z}_1} \quad \textit{and} \quad \tilde{z}_3 = \frac{\sqrt{\kappa_1\kappa_3\tilde{z}_1\tilde{z}_2}}{\kappa_2}.
$$

3.2.3 Monotonicity of Actuation

Next, we study the monotonicity of the control action u with respect to z^+ and z^0, that is the monotonicity of the actuation function $\mathcal{U}$. For convenience, we rewrite the three equations in (16) as

$$
\begin{cases} 2\tilde{z}_1 + \tilde{z}_3 + \sqrt{\kappa_3\tilde{z}_1} = z^0 \\ 2\tilde{z}_2 + \tilde{z}_3 + \sqrt{\kappa_1\tilde{z}_2} = z^+ \\ -\kappa_2\tilde{z}_3 + \sqrt{\kappa_1\kappa_3\tilde{z}_1\tilde{z}_2} = 0. \end{cases}
$$

By differentiating the three equations with respect to z^+, we obtain the following linear system of equations

$$
\underbrace{\begin{bmatrix} 2 + \frac{1}{2}\sqrt{\frac{\kappa_3}{\tilde{z}_1}} & 0 & 1 \\ 0 & 2 + \frac{1}{2}\sqrt{\frac{\kappa_1}{\tilde{z}_2}} & 1 \\ \frac{1}{2}\sqrt{\frac{\kappa_1\kappa_3\tilde{z}_2}{\tilde{z}_1}} & \frac{1}{2}\sqrt{\frac{\kappa_1\kappa_3\tilde{z}_1}{\tilde{z}_2}} & -\kappa_2 \end{bmatrix}}_{A} \begin{bmatrix} \frac{\partial\tilde{z}_1}{\partial z^+} \\ \frac{\partial\tilde{z}_2}{\partial z^+} \\ \frac{\partial\tilde{z}_3}{\partial z^+} \end{bmatrix} = \underbrace{\begin{bmatrix} 0 \\ 1 \\ 0 \end{bmatrix}}_{b^+}.
$$

Then by Cramer's rule we obtain

$$
\frac{\partial\tilde{z}_2}{\partial z^+} = -\frac{\kappa_2\left(2 + \frac{1}{2}\sqrt{\frac{\kappa_3}{\tilde{z}_1}}\right) + \frac{1}{2}\sqrt{\frac{\kappa_1\kappa_3\tilde{z}_2}{\tilde{z}_1}}}{\det(A)},
$$

where

$$
\det(A) = -\left(2 + \frac{1}{2}\sqrt{\frac{\kappa_3}{\tilde{z}_1}}\right)\left[\kappa_2\left(2 + \frac{1}{2}\sqrt{\frac{\kappa_1}{\tilde{z}_2}}\right) + \frac{1}{2}\sqrt{\frac{\kappa_1\kappa_3\tilde{z}_1}{\tilde{z}_2}}\right] - \frac{1}{2}\sqrt{\frac{\kappa_1\kappa_3\tilde{z}_2}{\tilde{z}_1}}\left(2 + \frac{1}{2}\sqrt{\frac{\kappa_1}{\tilde{z}_2}}\right) < 0.
$$

Hence, we have

$$
\frac{\partial\tilde{z}_2}{\partial z^+} > 0 \qquad \Longrightarrow \frac{\partial\mathcal{U}(z^+, z^0)}{\partial z^+} > 0.
$$

This means that the control action $u = \mathcal{U}(z^+, z^0)$ is a strictly monotonically increasing function of z^+.

Next, by differentiating the three equations with respect to z^0 , we obtain the following linear system of equations

$$\underbrace{\begin{bmatrix} 2+\frac{1}{2}\sqrt{\frac{\kappa_3}{\tilde{z}_1}} & 0 & 1 \\ 0 & 2+\frac{1}{2}\sqrt{\frac{\kappa_1}{\tilde{z}_2}} & 1 \\ \frac{1}{2}\sqrt{\frac{\kappa_1\kappa_3\tilde{z}_2}{\tilde{z}_1}} & \frac{1}{2}\sqrt{\frac{\kappa_1\kappa_3\tilde{z}_1}{\tilde{z}_2}} & -\kappa_2 \end{bmatrix}}_{A} \begin{bmatrix} \frac{\partial \tilde{z}_1}{\partial z^0} \\ \frac{\partial \tilde{z}_2}{\partial z^0} \\ \frac{\partial \tilde{z}_3}{\partial z^0} \end{bmatrix} = \underbrace{\begin{bmatrix} 1 \\ 0 \\ 0 \end{bmatrix}}_{b^0}.$$

Then by Cramer's rule we obtain

$$\frac{\partial \tilde{z}_2}{\partial z^0} = \frac{\frac{1}{2}\sqrt{\frac{\kappa_1\kappa_3\tilde{z}_2}{\tilde{z}_1}}}{\det(A)}.$$

Hence, we have

$$\frac{\partial \tilde{z}_2}{\partial z^+} < 0 \qquad \Longrightarrow \frac{\partial \mathcal{U}(z^+, z^0\ )}{\partial z^+} < 0.$$

Therefore, $\mathcal{U}(z^+, z^0\ )$ is monotonically increasing in z^+ and monotonically decreasing in z^0 .

3.2.4 Simulation Results

To demonstrate the accuracy of the model reduction result via simulations, we use the two regulated networks depicted in Supplementary Fig. 5(a) as examples. Regulated Network 1 is comprised of two species $\mathbf{X_1}$ and $\mathbf{X_2}$ and may represent a simple model for gene expression; whereas Regulated Network 2 is more complex and is comprised of six species $\mathbf{X_1}$ through $\mathbf{X_6}$ (taken from [2, 6]). The simulation results for both regulated networks are depicted in Supplementary Fig. 5(c) demonstrating that the reduced model captures the dynamics of the full model to a high degree of accuracy as expected.

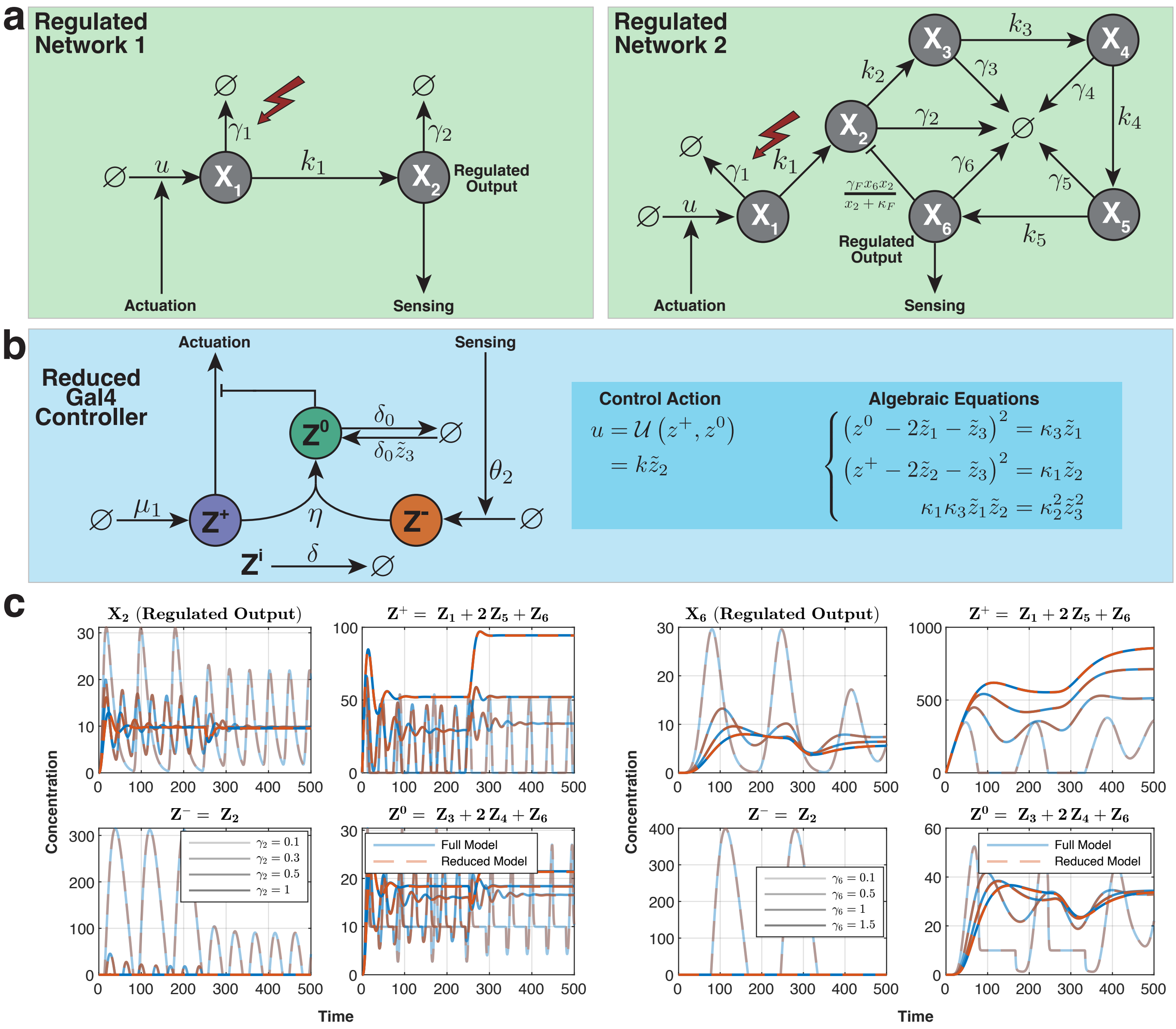

Supplementary Figure 5: Accuracy of the reduced model of the Gal4 controller. (a) Two test regulated networks. The first regulated network is comprised of two species $\mathbf{X_1}$ and $\mathbf{X_2}$, where $\mathbf{X_2}$ denotes the regulated output. The second regulated network is comprised of six species $\mathbf{X_1}$ through $\mathbf{X_6}$, where $\mathbf{X_6}$ denotes the regulated output. Both regulated networks are actuated by the control action u entering the dynamics as a production rate of the input species $\mathbf{X_1}$. (b) The reduced model of the Gal4 controller. The motif of the reduced model is comprised of the three effective species $\mathbf{Z^+}$, $\mathbf{Z^-}$ and $\mathbf{Z^0}$, as per Theorem 2, coupled to the three algebraic equations in $\tilde{z}_1, \tilde{z}_2$ and $\tilde{z}_3$. (c) Simulations demonstrating the accuracy of the reduced model of the controller. The simulations are carried out for the closed-loops where the Gal4 controller is connected in a feedback interconnection with each of the two regulated networks. The simulations are carried out starting from zero initial conditions at $t = 0$ and by doubling the degradation rate γ_1 of the input species $\mathbf{X_1}$ as a disturbance at $t = 250$. The simulations are also carried out for four different (initial) values of the degradation rates of the regulated outputs. The simulation results show that the reduced model is capable of accurately capturing the full dynamics of the full controller.
Numerical values. Regulated Network 1: $\gamma_1 = 0.1, k_1 = 1, k = 0.05, \delta_0 = \theta_2 = 1, \delta = 0.005, \mu_1 = \eta = 10, a_i = d_i = 100$ for $i = 1, 2, 3$.
Regulated Network 2: $\gamma_i = k_i = 0.1$ for $i = 1, \cdots, 5$, $\gamma_F = 0.3, \kappa_F = 1, k = 0.05, \delta_0 = \theta_2 = 1, \delta = 0.005, \mu_1 = \eta = 10, a_i = d_i = 100$ for $i = 1, 2, 3$.

3.3 intraDD Controller Circuit

3.3.1 Full Model Description

Let DBD, DD and AD denote the DNA binding domain, dimerization domain and activation domain, respectively. Furthermore, let $DD_{1/2}$ denote half of the dimerization domain which is not functional. The various controller species and reactions are listed in Supplementary Tables 8 and 9, respectively.

Species	Symbol	Species	Symbol
DBD:$DD_{1/2}$:Int^C:$DD_{1/2}$:AD	$\mathbf{Z_1}$	$\mathbf{Z_3}$	DBD:$DD_{1/2}$
Int^N	$\mathbf{Z_2}$	$\mathbf{Z_1}$:$\mathbf{Z_1}$	$\mathbf{Z_4}$

Supplementary Table 8: List of Biochemical Species

Reaction	Mechanism	Constants
Set-Point	$\varnothing \xrightarrow{\mu_1} \mathbf{Z_1}$	
Sensing	$\mathbf{X_L} \xrightarrow{\theta_2} \mathbf{X_L} + \mathbf{Z_2}$	
Binding	$\mathbf{Z_1} + \mathbf{Z_1} \underset{d}{\overset{a}{\rightleftharpoons}} \mathbf{Z_4}$	$\kappa := \frac{d}{a}$
Intein-Splicing 1	$\mathbf{Z_1} + \mathbf{Z_2} \xrightarrow{\eta} \mathbf{Z_3}$	
Intein-Splicing 2	$\mathbf{Z_4} + \mathbf{Z_2} \xrightarrow{2\eta} \mathbf{Z_1} + \mathbf{Z_3}$	
Degradation	$\mathbf{Z_3} \xrightarrow{\delta_0} \varnothing$	
Dilution	$\mathbf{Z}_i \xrightarrow{\delta} \varnothing, \qquad i = 1, \cdots, 4$	
Actuation	$\varnothing \xrightarrow{u} \mathbf{X_1}$	$u := kz_4$

Supplementary Table 9: List of Biochemical Reactions

3.3.2 Model Reduction

In this section, we apply Theorem 2 to obtain a reduced model for the detailed controller described in Supplementary Tables 8 and 9. The full controller model is schematically presented in Fig. 3 where $\mathbf{Z_4}$ act as an activator as indicated in the actuation function $u = h^+(z) = kz_4$. Under the assumption that the dimerization reaction is fast (i.e. $a = \tilde{a}/\epsilon, d = \tilde{d}/\epsilon$, as $\epsilon \to 0$), one can easily check that all the conditions of Theorem 2 are satisfied using the charge vectors q^+, q^- and q^0 given in Fig. 3. Applying Theorem 2 yields the following state variables

$$\begin{cases} z^+ &= z_1 + 2z_4 \\ z^- &= z_2 \\ z^0 &= z_3, \end{cases}$$

to arrive at the reduced model given by

$$\begin{cases} \dot{z}^+ &= \mu_1 - \eta z^+ z^- - \delta z^+ \\ \dot{z}^- &= \theta_2 x_L - \eta z^+ z^- - \delta z^- \\ \dot{z}^0 &= \eta z^+ z^- - (\delta + \delta_0) z^0 \ , \end{cases}$$

and the control action u calculated as

$$u = h^+ \left(W_1^T z^{\text{tot}} + W_2^T \tilde{z} \right) = h^+ \left(\begin{bmatrix} z^+ - 2\tilde{z} \\ z^- \\ z^0 \\ \tilde{z} \end{bmatrix}, x_L \right) = k\tilde{z},$$

where $\tilde{z}$ is given implicitly by the following algebraic equation

$$\begin{aligned} \tilde{C} S_B \tilde{\lambda}_B \left(W_1^T z^{\text{tot}} + W_2^T \tilde{z} \right) = 0 &\implies \tilde{a} \left(z^+ - 2\tilde{z} \right)^2 - \tilde{d}\tilde{z} = 0 \\ &\implies \left(z^+ - 2\tilde{z} \right)^2 - \kappa \tilde{z} = 0 \\ &\implies \tilde{z} = \frac{1}{8} \left(4z^+ + \kappa \pm \sqrt{8\kappa z^+ + \kappa^2} \right). \end{aligned} \tag{17}$$

Observe that $\tilde{z}$ has two possible values. However, recalling that $z^+ - 2\tilde{z} \geq 0$ allows us to rule out one the two solutions to obtain

$$\tilde{z} = \frac{1}{8}\left(4z^+ + \kappa - \sqrt{8\kappa z^+ + \kappa^2}\right).$$

As a result, one can think of the control action u as a function $\mathcal{U}(z^+)$ defined explicitly as

$$u = \mathcal{U}(z^+) = \frac{k}{8}\left(4z^+ + \kappa - \sqrt{8\kappa z^+ + \kappa^2}\right).$$

Remark 5. *Observe that the reduced model for the controller dynamics is governed by a set of Ordinary Differential Equations (ODEs) that can be numerically solved easily. This is due to the fact that the algebraic equation of the reduced model can be explicitly solved for $\tilde{z}$ here.*

Remark 6. *Observe that the motif of the reduced model does not involve the production term $\delta_0(q^{\pm,0})^T\psi(z^{tot})$ for z^0, because no controller species carries mixed charges simultaneously, that is $q^{\pm,0} = 0$.*

3.3.3 Monotonicity of Actuation

Next, we study the monotonicity of the control action u with respect to z^+, that is the monotonicity of the actuation function $\mathcal{U}$. We have

$$\frac{\partial\mathcal{U}(z^+)}{\partial z^+} = \frac{k}{2}\left(1 - \frac{1}{\sqrt{1 + 8z^+/\kappa}}\right) > 0,$$

and thus the control action u is monotonically increasing in z^+.

3.3.4 Simulation Results

To demonstrate the accuracy of the model reduction result via simulations, we use the two regulated networks depicted in Supplementary Fig. 6(a) as examples. Regulated Network 1 is comprised of two species $\mathbf{X_1}$ and $\mathbf{X_2}$ and may represent a simple model for gene expression; whereas Regulated Network 2 is more complex and is comprised of six species $\mathbf{X_1}$ through $\mathbf{X_6}$ (taken from [2, 6]). The simulation results for both regulated networks are depicted in Supplementary Fig. 6(c) demonstrating that the reduced model captures the dynamics of the full model to a high degree of accuracy as expected.

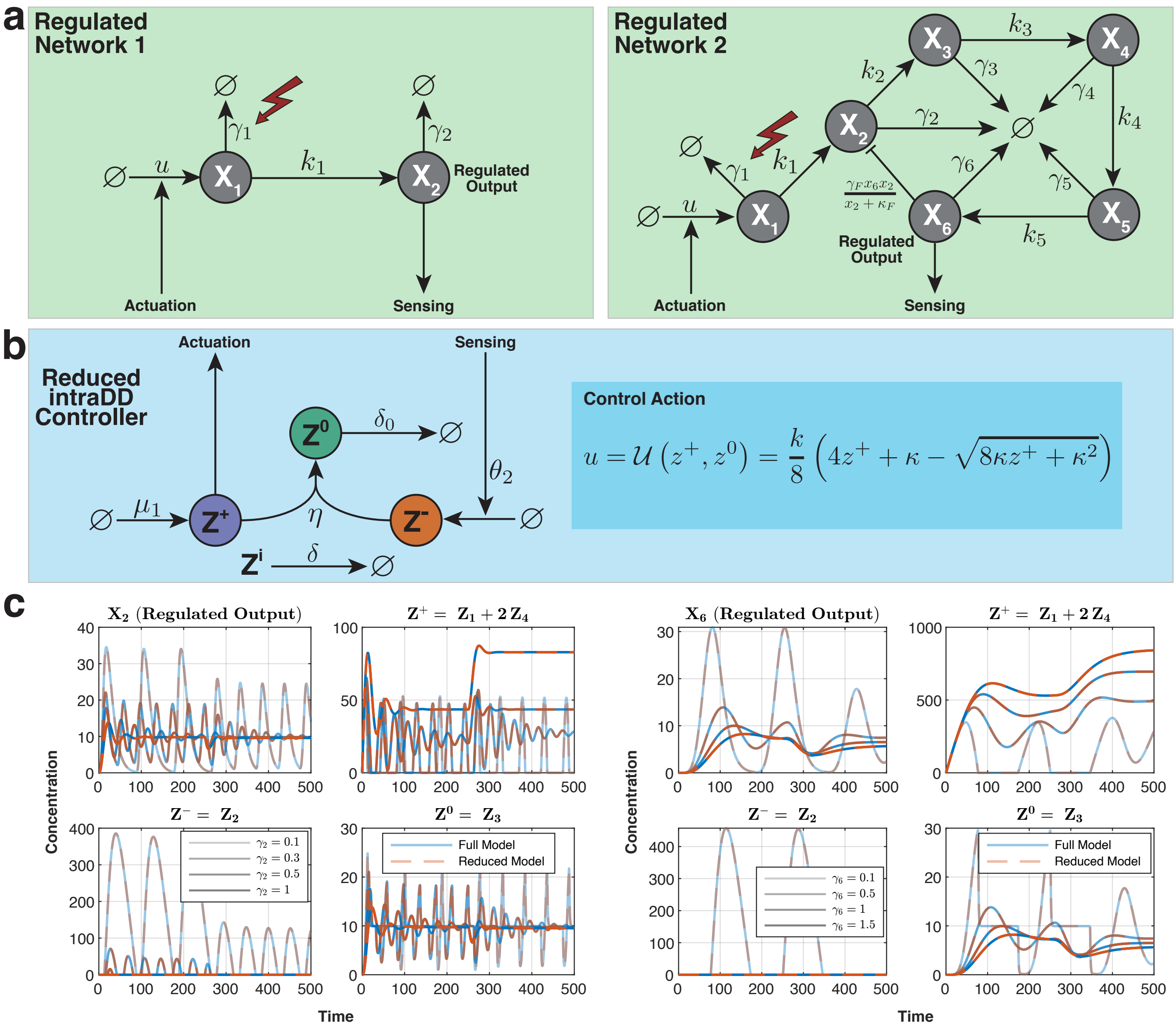

Supplementary Figure 6: Accuracy of the reduced model of the intraDD controller. (a) Two test regulated networks. The first regulated network is comprised of two species $\mathbf{X_1}$ and $\mathbf{X_2}$, where $\mathbf{X_2}$ denotes the regulated output. The second regulated network is comprised of six species $\mathbf{X_1}$ through $\mathbf{X_6}$, where $\mathbf{X_6}$ denotes the regulated output. Both regulated networks are actuated by the control action u entering the dynamics as a production rate of the input species $\mathbf{X_1}$. (b) The reduced model of the intraDD controller. The motif of the reduced model is comprised of the three effective species $\mathbf{Z^+}$, $\mathbf{Z^-}$ and $\mathbf{Z^0}$, as per Theorem 2. (c) Simulations demonstrating the accuracy of the reduced model of the controller. The simulations are carried out for the closed-loops where the intraDD controller is connected in a feedback interconnection with each of the two regulated networks. The simulations are carried out starting from zero initial conditions at $t = 0$ and by doubling the degradation rate γ_1 of the input species $\mathbf{X_1}$ as a disturbance at $t = 250$. The simulations are also carried out for four different (initial) values of the degradation rates of the regulated outputs. The simulation results show that the reduced model is capable of accurately capturing the full dynamics of the full controller.
Numerical values. Regulated Network 1: $\gamma_1 = 0.1, k_1 = 1, k = 0.05, \delta_0 = \theta_2 = 1, \delta = 0.005, \mu_1 = \eta = 10, a = d = 100$.
Regulated Network 2: $\gamma_i = k_i = 0.1$ for $i = 1, \cdots, 5$, $\gamma_F = 0.3, \kappa_F = 1, k = 0.05, \delta_0 = \theta_2 = 1, \delta = 0.005, \mu_1 = \eta = 10, a = d = 100$.

3.4 ZF Controller Circuit

Let DBD, DD and AD denote the DNA binding domain, dimerization domain and activation domain, respectively. The various controller species and reactions are listed in Supplementary Tables 10 and 11, respectively.

The controller dynamics are thus given by the following set of differential equations.

$$\begin{cases} \dot{z}_1 = \mu_1 - \eta z_1 z_2 - \delta z_1 \\ \dot{z}_2 = \theta_2 x_L - \eta z_1 z_2 - \delta z_2 \\ \dot{z}_3 = \eta z_1 z_2 - (\delta + \delta_0) z_3. \end{cases}$$

Species	Symbol	Species	Symbol
DBD:Int^{C}:AD	$\mathbf{Z_1}$	$\mathbf{Z_3}$	DBD
Int^{N}	$\mathbf{Z_2}$		

Supplementary Table 10: List of Biochemical Species

Reaction	Mechanism	Constants
Set-Point	$\varnothing \xrightarrow{\mu_1} \mathbf{Z_1}$	
Sensing	$\mathbf{X_L} \xrightarrow{\theta_2} \mathbf{X_L} + \mathbf{Z_2}$	
Intein-Splicing	$\mathbf{Z_1} + \mathbf{Z_2} \xrightarrow{\eta} \mathbf{Z_3}$	
Degradation	$\mathbf{Z_3} \xrightarrow{\delta_0} \varnothing$	
Dilution	$\mathbf{Z_i} \xrightarrow{\delta} \varnothing, \qquad i = 1,2,3$	
Actuation	$\varnothing \xrightarrow{u} \mathbf{X_1}$	$u := \frac{kz_1}{1+\frac{z_3}{\kappa_u}}$

Supplementary Table 11: List of Biochemical Reactions

Since there are no binding or conversion reactions, the model is already in its reduced form. In fact, we have $z^+ = z_1, z^- = z_2$ and $z^0 = z_3$.

3.5 Inactive-Intein Controller Circuit

3.5.1 Full Model Description

Let DBD, DD, AD and i-Int^{N} denote the DNA binding domain, dimerization domain, activation domain and inactive (dead) Int^{N}, respectively. The various controller species and reactions are listed in Supplementary Tables 12 and 13, respectively.

Species	Symbol	Species	Symbol
Int^{C}:AD	$\mathbf{Z_1}$	$\mathbf{Z_4}$:$\mathbf{Z_4}$	$\mathbf{Z_5}$
Int^{N}	$\mathbf{Z_2}$	$\mathbf{Z_1}$:$\mathbf{Z_5}$	$\mathbf{Z_6}$
Int^{C}:Int^{N} + AD	$\mathbf{Z_3}$	$\mathbf{Z_1}$:$\mathbf{Z_6}$	$\mathbf{Z_7}$
DBD:DD:i-Int^{N}	$\mathbf{Z_4}$	$\mathbf{Z_1}$:$\mathbf{Z_4}$	$\mathbf{Z_8}$

Supplementary Table 12: List of Biochemical Species

The ordinary differential equations describing the controller dynamics are given by

$$\textbf{Full Model}: \begin{cases} \dot{z}_1 = \mu_1 - \eta z_1 z_2 - \delta z_1 - (a_2 z_1 z_5 - d_2 z_6) - (a_3 z_1 z_6 - d_3 z_7) - (a_4 z_1 z_4 - d_4 z_8) \\ \dot{z}_2 = \theta_2 x_L - \eta z_1 z_2 - \delta z_2 \\ \dot{z}_3 = \eta z_1 z_2 - (\delta + \delta_0) z_3 \\ \dot{z}_4 = \mu_4 - (\delta + \delta_0) z_4 - (a_4 z_1 z_4 - d_4 z_8) - (a_6 z_4 z_8 - d_6 z_6) \\ \dot{z}_5 = -(\delta + \delta_0) z_5 + (a_1 z_4^2 - d_1 z_5) - (a_2 z_1 z_5 - d_2 z_6) \\ \dot{z}_6 = -\delta z_6 + (a_2 z_1 z_5 - d_2 z_6) - (a_3 z_1 z_6 - d_3 z_7) + (a_6 z_4 z_8 - d_6 z_6) \\ \dot{z}_7 = -\delta z_7 + (a_3 z_1 z_6 - d_3 z_7) + (a_5 z_8^2 - d_3 z_7) \\ \dot{z}_8 = -\delta z_8 + (a_4 z_1 z_4 - d_4 z_8) - 2(a_5 z_8^2 - d_5 z_7) - (a_6 z_4 z_8 - d_6 z_6). \end{cases}$$

Reaction	Mechanism	Constants
Set-Point	$\varnothing \xrightarrow{\mu_1} \mathbf{Z_1}$	
Sensing	$\mathbf{X_L} \xrightarrow{\theta_2} \mathbf{X_L} + \mathbf{Z_2}$	
Production	$\varnothing \xrightarrow{\mu_4} \mathbf{Z_4}$	
Binding 1	$\mathbf{Z_4} + \mathbf{Z_4} \underset{d_1}{\overset{a_1}{\rightleftharpoons}} \mathbf{Z_5}$	$\kappa_1 := \frac{d_1}{a_1}$
Binding 2	$\mathbf{Z_1} + \mathbf{Z_5} \underset{d_2}{\overset{a_2}{\rightleftharpoons}} \mathbf{Z_6}$	$\kappa_2 := \frac{d_2}{a_2}$
Binding 3	$\mathbf{Z_1} + \mathbf{Z_6} \underset{d_3}{\overset{a_3}{\rightleftharpoons}} \mathbf{Z_7}$	$\kappa_3 := \frac{d_3}{a_3}$
Binding 4	$\mathbf{Z_1} + \mathbf{Z_4} \underset{d_4}{\overset{a_4}{\rightleftharpoons}} \mathbf{Z_8}$	$\kappa_4 := \frac{d_4}{a_4}$
Binding 5	$\mathbf{Z_8} + \mathbf{Z_8} \underset{d_5}{\overset{a_5}{\rightleftharpoons}} \mathbf{Z_7}$	$\kappa_5 := \frac{d_5}{a_5}$
Binding 6	$\mathbf{Z_4} + \mathbf{Z_8} \underset{d_6}{\overset{a_6}{\rightleftharpoons}} \mathbf{Z_6}$	$\kappa_6 := \frac{d_6}{a_6}$
Intein-Splicing	$\mathbf{Z_1} + \mathbf{Z_2} \xrightarrow{\eta} \mathbf{Z_3}$	
Degradation	$\mathbf{Z}_i \xrightarrow{\delta_0} \varnothing, \qquad i = 3, 4, 5$	
Dilution	$\mathbf{Z}_i \xrightarrow{\delta} \varnothing, \qquad i = 1, \cdots, 8$	
Actuation	$\varnothing \xrightarrow{u} \mathbf{X_1}$	$u := \frac{kz_7 + k'z_6}{1 + z_5/\kappa_u}$

Supplementary Table 13: List of Biochemical Reactions

3.5.2 Model Reduction

In this section, we derive a reduced model for the detailed controller described in Supplementary Tables 12 and 13. The full controller model is schematically presented in Fig. 7 where $\mathbf{Z_6}$ and $\mathbf{Z_7}$ act as activators while $\mathbf{Z_5}$ act as a repressor as indicated in the actuation function $u = h(z) = \frac{kz_7 + k'z_6}{1 + z_5/\kappa_u}$. We assume that the dimerization reactions are fast (i.e. $a_i = \tilde{a}_i/\epsilon, d_i = \tilde{d}_i/\epsilon$, for $i = 1, \cdots, 6$, as $\epsilon \to 0$). For this circuit, Theorem 2 does not apply, and in fact the three vectors q^+, q^- and q^0 are not enough to carry out the model reduction. An additional vector $q^\star$ denoting the number of dead $\mathrm{Int}^{\mathrm{N}}$ segements is required here. We have

$$\begin{aligned} q^+ &= \begin{bmatrix} 1 & 0 & 0 & 0 & 0 & 1 & 2 & 1 \end{bmatrix} \\ q^- &= \begin{bmatrix} 0 & 1 & 0 & 0 & 0 & 0 & 0 & 0 \end{bmatrix} \\ q^0 &= \begin{bmatrix} 0 & 0 & 1 & 0 & 0 & 0 & 0 & 0 \end{bmatrix} \\ q^\star &= \begin{bmatrix} 0 & 0 & 0 & 1 & 2 & 2 & 2 & 1 \end{bmatrix}, \end{aligned}$$

and hence with $z^i := q^i z$ for $i \in \{+, -, 0, \star\}$, we define the following state variables

$$\begin{cases} z^+ = z_1 + z_6 + 2z_7 + z_8 \\ z^- = z_2 \\ z^0 = z_3 \\ z^\star = z_4 + 2z_5 + 2z_6 + 2z_7 + z_8. \end{cases}$$

Using this state transformation, the full model of the controller dynamics can be rewritten in the new coordinates

$$\begin{bmatrix} z^+ & z^- & z^0 & z^\star & z_5 & z_6 & z_7 & z_8 \end{bmatrix}^T,$$

as follows

$$\textbf{Transformed Full Model}: \begin{cases} \dot{z}^+ = \mu_1 - \eta z_1 z^- - \delta z^+ \\ \dot{z}^- = \theta_2 x_L - \eta z_1 z^- - \delta z^- \\ \dot{z}^0 = \eta z_1 z^- - (\delta+\delta_0) z^0 \\ \dot{z}^\star = \mu_4 - (\delta+\delta_0) z^\star + \delta_0(2z_6 + 2z_7 + z_8) \\ \dot{z}_5 = -(\delta+\delta_0) z_5 + (a_1 z_4^2 - d_1 z_5) - (a_2 z_1 z_5 - d_2 z_6) \\ \dot{z}_6 = -\delta z_6 + (a_2 z_1 z_5 - d_2 z_6) - (a_3 z_1 z_6 - d_3 z_7) + (a_6 z_4 z_8 - d_6 z_6) \\ \dot{z}_7 = -\delta z_7 + (a_3 z_1 z_6 - d_3 z_7) + (a_5 z_8^2 - d_3 z_7) \\ \dot{z}_8 = -\delta z_8 + (a_4 z_1 z_4 - d_4 z_8) - 2(a_5 z_8^2 - d_5 z_7) - (a_6 z_4 z_8 - d_6 z_6), \end{cases}$$

where $\quad z_1 = z^+ - z_6 - 2z_7 - z_8 \quad$ and $\quad z_4 = z^\star - 2z_5 - 2z_6 - 2z_7 - z_8$.

Note that this transformation managed to separate the time scales since the a_i's and d_i's do not appear in the first four differential equations. As a result, $(z^+, z^-, z^0, z^\star)$ can be viewed as the slow variables while (z_5, z_6, z_7, z_8) can be viewed as the fast variables. In fact, the controller dynamics can be written in the standard singular perturbation form by writing $a_i := \tilde{a}_i/\epsilon$ and $d_i := \tilde{d}_i/\epsilon$ for $i = 1, \cdots, 6$ to yield

$$\textbf{Singular Perturbation Form}: \begin{cases} \dot{z}^+ = \mu_1 - \eta z_1 z^- - \delta z^+ \\ \dot{z}^- = \theta_2 x_L - \eta z_1 z^- - \delta z^- \\ \dot{z}^0 = \eta z_1 z^- - (\delta+\delta_0) z^0 \\ \dot{z}^\star = \mu_4 - (\delta+\delta_0) z^\star + \delta_0(2z_6 + 2z_7 + z_8) \\ \epsilon\dot{z}_5 = -\epsilon(\delta+\delta_0) z_5 + (\tilde{a}_1 z_4^2 - \tilde{d}_1 z_5) - (\tilde{a}_2 z_1 z_5 - \tilde{d}_2 z_6) \\ \epsilon\dot{z}_6 = -\epsilon\delta z_6 + (\tilde{a}_2 z_1 z_5 - \tilde{d}_2 z_6) - (\tilde{a}_3 z_1 z_6 - \tilde{d}_3 z_7) + (\tilde{a}_6 z_4 z_8 - \tilde{d}_6 z_6) \\ \epsilon\dot{z}_7 = -\epsilon\delta z_7 + (\tilde{a}_3 z_1 z_6 - \tilde{d}_3 z_7) + (\tilde{a}_5 z_8^2 - \tilde{d}_3 z_7) \\ \epsilon\dot{z}_8 = -\epsilon\delta z_8 + (\tilde{a}_4 z_1 z_4 - \tilde{d}_4 z_8) - 2(\tilde{a}_5 z_8^2 - \tilde{d}_5 z_7) - (\tilde{a}_6 z_4 z_8 - \tilde{d}_6 z_6), \end{cases}$$

where $\quad z_1 = z^+ - z_6 - 2z_7 - z_8 \quad$ and $\quad z_4 = z^\star - 2z_5 - 2z_6 - 2z_7 - z_8$.

By taking ϵ to zero, the eight differential equations reduce to four differential equations subject to 4 algebraic constraints which dictate the slow manifold. That is, we have

$$\textbf{Reduced Model}: \begin{cases} \dot{z}^+ = \mu_1 - \eta\phi(z^+, z^\star) z^- - \delta z^+ \\ \dot{z}^- = \theta_2 x_L - \eta\phi(z^+, z^\star) z^- - \delta z^- \\ \dot{z}^0 = \eta\phi(z^+, z^\star) z^- - (\delta+\delta_0) z^0 \\ \dot{z}^\star = \mu_4 - (\delta+\delta_0) z^\star + \delta_0 \psi(z^+, z^\star) \end{cases}$$

where $\phi(z^+, z^\star) := z^+ - z_6 - 2z_7 - z_8$, $\psi(z^+, z^\star) := 2z_6 + 2z_7 + z_8$ and the control action $u = \mathcal{U}(z^+, z^\star) := \frac{kz_7 + k' z_6}{1 + z_5/\kappa_u}$ such that z_5, z_6, z_7 and z_8 solve the following nonlinear algebraic equations

$$\begin{cases} (\tilde{a}_1 z_4^2 - \tilde{d}_1 z_5) - (\tilde{a}_2 z_1 z_5 - \tilde{d}_2 z_6) = 0 \\ (\tilde{a}_2 z_1 z_5 - \tilde{d}_2 z_6) - (\tilde{a}_3 z_1 z_6 - \tilde{d}_3 z_7) + (\tilde{a}_6 z_4 z_8 - \tilde{d}_6 z_6) = 0 \\ (\tilde{a}_3 z_1 z_6 - \tilde{d}_3 z_7) + (\tilde{a}_5 z_8^2 - \tilde{d}_3 z_7) = 0 \\ (\tilde{a}_4 z_1 z_4 - \tilde{d}_4 z_8) - 2(\tilde{a}_5 z_8^2 - \tilde{d}_5 z_7) - (\tilde{a}_6 z_4 z_8 - \tilde{d}_6 z_6) = 0. \end{cases}$$

The reduced differential equations coupled with the implicit algebraic equations give rise to a system of *Differential Algebraic Equations (DAEs)* that can be easily solved in MATLAB using ODE15i for example. Note that, for full mathematical rigor, one has to still prove that the slow manifold is stable to establish the validity of the reduced model. Unlike the structural result in Supplementary Theorem 2 which resorts to the zero-deficiency theorem, this task is not easy to do here. To this end, the validation for the reduced model of this circuit is carried out by resorting to the simulations given in Fig. 7 where the two-species network was used as the regulated network.

Numerical values. $k_1 = \gamma_1 = \gamma_2 = \theta_2 = \kappa_u = \delta_0 = 1, a_i = d_i = 100$ for $i = 1, \cdots, 6$, $\eta = 10, \mu_4 = 5; k = 1, k' = 0.5, \delta = 0.01$.

4 Design and Analysis of Various Integral Controllers Mathematically Realized as Chemical Reaction Networks

In this section, we provide several examples of controllers that are mathematically designed based on Theorems 1 and 2. These examples demonstrate that the RPA and model reduction theorems are not restricted to intein-based controllers only, and that they can be applied to more mathematically abstract controllers realized as chemical reaction networks.

4.1 Multiple-Dimer Controller Circuit

4.1.1 Full Model Description

Consider the various controller reactions listed in Supplementary Table 14. Observe that, in this example controller, $\mathbf{Z_2}$ can also dimerize, and not just $\mathbf{Z_1}$ and its derivatives.

Reaction	Mechanism	Constants
Set-Point	$\varnothing \xrightarrow{\mu_1} \mathbf{Z_1}$	
Sensing	$\mathbf{X_L} \xrightarrow{\theta_2} \mathbf{X_L} + \mathbf{Z_2}$	
Binding 1	$\mathbf{Z_1} + \mathbf{Z_1} \underset{d_1}{\overset{a_1}{\rightleftharpoons}} \mathbf{Z_4}$	$\kappa_1 := \frac{d_1}{a_1}$
Binding 2	$\mathbf{Z_2} + \mathbf{Z_2} \underset{d_2}{\overset{a_2}{\rightleftharpoons}} \mathbf{Z_5}$	$\kappa_2 := \frac{d_2}{a_2}$
Binding 3	$\mathbf{Z_3} + \mathbf{Z_3} \underset{d_3}{\overset{a_3}{\rightleftharpoons}} \mathbf{Z_6}$	$\kappa_3 := \frac{d_3}{a_3}$
Sequestration 1	$\mathbf{Z_1} + \mathbf{Z_2} \xrightarrow{\eta} \mathbf{Z_3}$	
Sequestration 2	$\mathbf{Z_4} + \mathbf{Z_5} \xrightarrow{2\eta} \mathbf{Z_6}$	
Sequestration 3	$\mathbf{Z_4} + \mathbf{Z_2} \xrightarrow{2\eta} \mathbf{Z_1} + \mathbf{Z_3}$	
Sequestration 4	$\mathbf{Z_1} + \mathbf{Z_5} \xrightarrow{2\eta} \mathbf{Z_2} + \mathbf{Z_3}$	
Degradation	$\mathbf{Z}_i \xrightarrow{\delta_0} \varnothing, \qquad i = 3, 6$	
Actuation	$\varnothing \xrightarrow{u} \mathbf{X_1}$	$u := \frac{kz_4}{1+\frac{z_5}{\kappa_u}+\frac{z_6}{\kappa_u'}}$

Supplementary Table 14: List of Reactions

4.1.2 Model Reduction

In this section, we apply Supplementary Theorem 2 to obtain a reduced model for the detailed controller described in Supplementary Table 14. The full controller model is schematically presented in Box 1 where $\mathbf{Z_5}$ and $\mathbf{Z_6}$ act as repressors while $\mathbf{Z_4}$ act as an activator as indicated in the actuation function $u = h^+(z) = \frac{kz_4}{1+z_5/\kappa_u+z_6/\kappa_u'}$. Under the assumption that the dimerization reactions are fast (i.e. $a_i = \tilde{a}_i/\epsilon, d_i = \tilde{d}_i/\epsilon$, for $i = 1, 2, 3$, as $\epsilon \to 0$), one can easily check that all the conditions of Supplementary Theorem 2 are satisfied using the charge vectors q^+, q^- and q^0 given in Box 1. Applying Supplementary Theorem 2 yields the following state variables

$$\begin{cases} z^+ &= z_1 + 2z_4 \\ z^- &= z_2 + 2z_5 \\ z^0 &= z_3 + 2z_6, \end{cases}$$

to arrive at the reduced model given by

$$\begin{cases} \dot{z}^+ &= \mu_1 - \eta z^+ z^- \\ \dot{z}^- &= \theta_2 x_L - \eta z^+ z^- \\ \dot{z}^0 &= \eta z^+ z^- - \delta_0 z^0 \ , \end{cases}$$

since $q^{\pm,0} = \mathbb{1}\left(q^+ + q^-\right) \circ q^0 = 0$, and the control action u is calculated as

$$u = h\left(W_1^T z^{\mathrm{tot}} + W_2^T \tilde{z}\right) = h\left(\begin{bmatrix} z^+ - 2\tilde{z}_1 \\ z^- - 2\tilde{z}_2 \\ z^0 - 2\tilde{z}_3 \\ \tilde{z}_1 \\ \tilde{z}_2 \\ \tilde{z}_3 \end{bmatrix}, x_L\right) = \frac{k\tilde{z}_1}{1 + \frac{\tilde{z}_2}{\kappa_u} + \frac{\tilde{z}_3}{\kappa_u'}},$$

where $\tilde{z} := \begin{bmatrix}\tilde{z}_1 & \tilde{z}_2 & \tilde{z}_3\end{bmatrix}^T$ is given implicitly by the following algebraic equation

$$\begin{aligned}
\tilde{C} S_B \tilde{\lambda}_B \left(W_1^T z^{\mathrm{tot}} + W_2^T \tilde{z}\right) = 0 &\implies \begin{cases} \tilde{a}_1 \left(z^+ - 2\tilde{z}_1\right)^2 - \tilde{d}_1 \tilde{z}_1 = 0 \\ \tilde{a}_2 \left(z^- - 2\tilde{z}_2\right)^2 - \tilde{d}_2 \tilde{z}_2 = 0 \\ \tilde{a}_3 \left(z^0 - 2\tilde{z}_3\right)^2 - \tilde{d}_3 \tilde{z}_3 = 0 \end{cases} \\
&\implies \begin{cases} \left(z^+ - 2\tilde{z}_1\right)^2 - \kappa_1 \tilde{z}_1 = 0 \\ \left(z^- - 2\tilde{z}_2\right)^2 - \kappa_2 \tilde{z}_2 = 0 \\ \left(z^0 - 2\tilde{z}_3\right)^2 - \kappa_3 \tilde{z}_3 = 0 \end{cases} \\
&\implies \begin{cases} \tilde{z}_1 = \frac{1}{8}\left(4z^+ + \kappa_1 \pm \sqrt{8\kappa_1 z^+ + \kappa_1^2}\right) \\ \tilde{z}_2 = \frac{1}{8}\left(4z^- + \kappa_2 \pm \sqrt{8\kappa_2 z^- + \kappa_2^2}\right) \\ \tilde{z}_3 = \frac{1}{8}\left(4z^0 + \kappa_3 \pm \sqrt{8\kappa_3 z^0 + \kappa_3^2}\right). \end{cases}
\end{aligned} \tag{18}$$

Observe that each $\tilde{z}_i$, for $i = 1, 2, 3$ has two possible values. However, recalling that $z^+ - 2\tilde{z}_1, z^- - 2\tilde{z}_2, z^0 - 2\tilde{z}_3 \geq 0$ allows us to rule out one the two solutions to obtain

$$\begin{cases} \tilde{z}_1 = \frac{1}{8}\left(4z^+ + \kappa_1 - \sqrt{8\kappa_1 z^+ + \kappa_1^2}\right) \\ \tilde{z}_2 = \frac{1}{8}\left(4z^- + \kappa_2 - \sqrt{8\kappa_2 z^- + \kappa_2^2}\right) \\ \tilde{z}_3 = \frac{1}{8}\left(4z^0 + \kappa_3 - \sqrt{8\kappa_3 z^0 + \kappa_3^2}\right). \end{cases}$$

As a result, one can think of the control action u as a function $\mathcal{U}(z^+, z^-, z^0)$ defined explicitly as

$$\begin{aligned}
u = \mathcal{U}(z^+, z^-, z^0) &= \frac{k\tilde{z}_1}{1 + \frac{\tilde{z}_2}{\kappa_u} + \frac{\tilde{z}_3}{\kappa_u'}} \\
&= \frac{8k\kappa_u\kappa_u'\left(4z^+ + \kappa_1 - \sqrt{8\kappa_1 z^+ + \kappa_1^2}\right)}{8\kappa_u\kappa_u' + \kappa_u'\left(4z^- + \kappa_2 - \sqrt{8\kappa_2 z^- + \kappa_2^2}\right) + \kappa_u\left(4z^0 + \kappa_3 - \sqrt{8\kappa_3 z^0 + \kappa_3^2}\right)}.
\end{aligned}$$

4.1.3 Monotonicity of Actuation

Next, we study the monotonicity of the control action u with respect to z^+, z^- and z^0, that is the monotonicity of the actuation function $\mathcal{U}$. We have

$$\begin{aligned}
\frac{\partial \tilde{z}_1}{\partial z^+} &= \frac{1}{2}\left(1 - \frac{1}{\sqrt{1 + 8z^+/\kappa_1}}\right) > 0 \\
\frac{\partial \tilde{z}_2}{\partial z^-} &= \frac{1}{2}\left(1 - \frac{1}{\sqrt{1 + 8z^-/\kappa_2}}\right) > 0 \\
\frac{\partial \tilde{z}_1}{\partial z^0} &= \frac{1}{2}\left(1 - \frac{1}{\sqrt{1 + 8z^0/\kappa_3}}\right) > 0.
\end{aligned}$$

Therefore, the control action $u = \mathcal{U}(z^+, z^-, z^0)$ is monotonically increasing in z^+; whereas, it is monotonically decreasing in z^- and z^0.

4.1.4 Simulation Results

To demonstrate the accuracy of the model reduction result via simulations, we use the two regulated networks depicted in Supplementary Fig. 7(a) as examples. Regulated Network 1 is comprised of two species $\mathbf{X_1}$ and $\mathbf{X_2}$ and may represent a simple model for gene expression; whereas Regulated Network 2 is more complex and is comprised of six species $\mathbf{X_1}$ through $\mathbf{X_6}$ (taken from [2, 6]). The simulation results for both regulated networks are depicted in Supplementary Fig. 7(c) demonstrating that the reduced model captures the dynamics of the full model to a high degree of accuracy as expected.

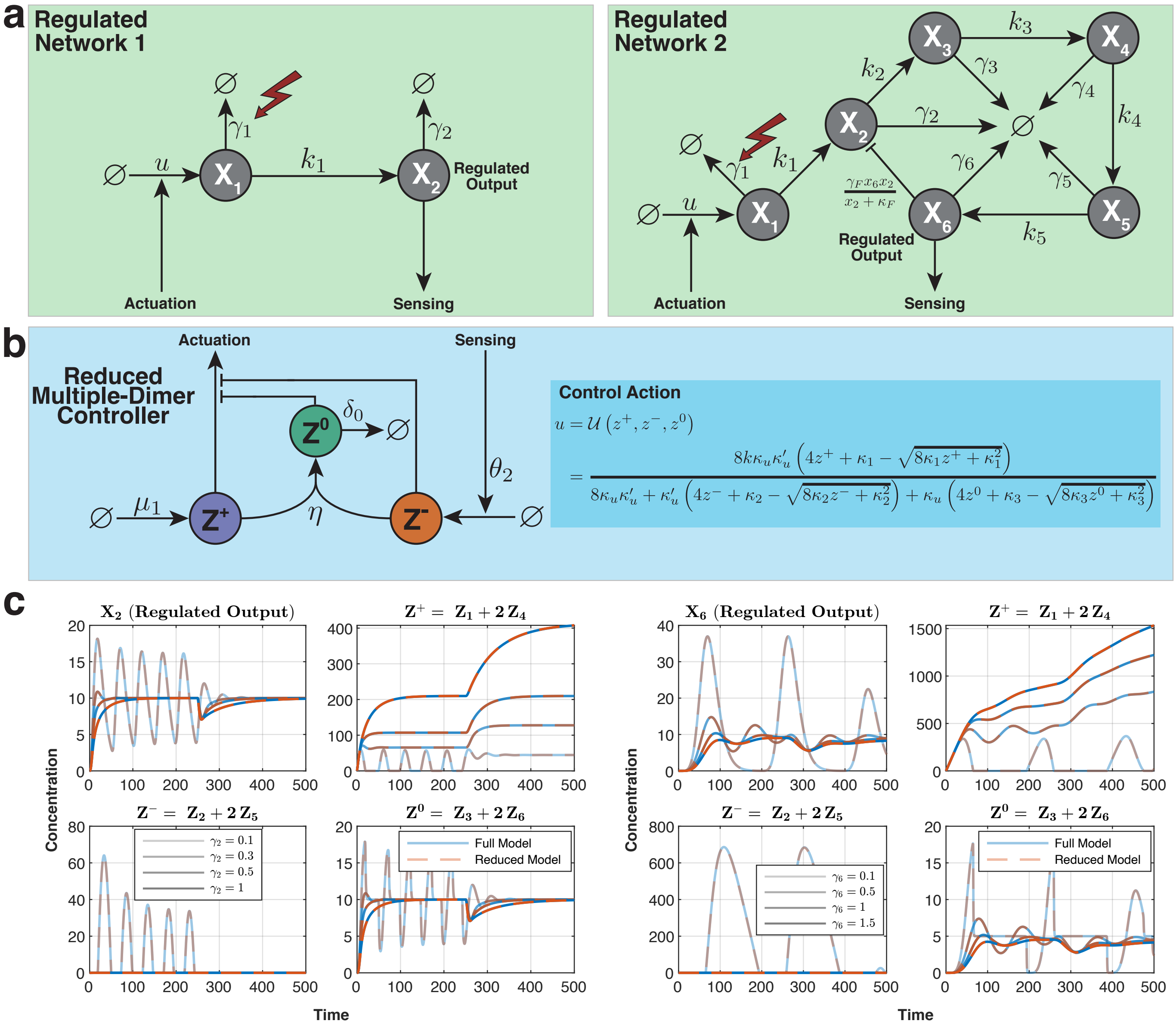

Supplementary Figure 7: Accuracy of the reduced model of the Multiple-Dimer controller. (a) Two test regulated networks. The first regulated network is comprised of two species $\mathbf{X_1}$ and $\mathbf{X_2}$, where $\mathbf{X_2}$ denotes the regulated output. The second regulated network is comprised of six species $\mathbf{X_1}$ through $\mathbf{X_6}$, where $\mathbf{X_6}$ denotes the regulated output. Both regulated networks are actuated by the control action u entering the dynamics as a production rate of the input species $\mathbf{X_1}$. (b) The reduced model of the Multiple-Dimer controller. The motif of the reduced model is comprised of the three effective species $\mathbf{Z^+}$, $\mathbf{Z^-}$ and $\mathbf{Z^0}$, as per Theorem 2. (c) Simulations demonstrating the accuracy of the reduced model of the controller. The simulations are carried out for the closed-loops where the Multiple-Dimer controller is connected in a feedback interconnection with each of the two regulated networks. The simulations are carried out starting from zero initial conditions at $t = 0$ and by doubling the degradation rate γ_1 of the input species $\mathbf{X_1}$ as a disturbance at $t = 250$. The simulations are also carried out for four different (initial) values of the degradation rates of the regulated outputs. The simulation results show that the reduced model is capable of accurately capturing the full dynamics of the full controller.

Numerical values. Regulated Network 1: $\gamma_1 = 0.1, k_1 = 1, \mu_1 = 10, \theta_2 = 1, k = 0.05, \kappa_u = \kappa_u' = \delta_0 = 1, \eta = 10, a_i = d_i = 100$ for $i = 1, 2, 3$. Regulated Network 2: $\gamma_i = k_i = 0.1$ for $i = 1, \cdots, 5$, $\gamma_F = 0.3, \kappa_F = 1, \mu_1 = 10, \theta_2 = 1, k = 0.1, \kappa_u = \kappa_u' = 1, \delta_0 = 2, \eta = 10, a_i = d_i = 100$ for $i = 1, 2, 3$.

4.2 Conversion Controller Circuit

4.2.1 Full Model Description

Consider the various controller reactions listed in Supplementary Table 15 and schematically illustrated in Supplementary Fig. 8. Observe that, in this example controller, $\mathbf{Z_1}$ and $\mathbf{Z_2}$ are reversible converted to $\mathbf{Z_4}$ and $\mathbf{Z_5}$, and a negative actuation reaction is present where the input species $\mathbf{X_1}$ is removed at a rate u^- that is proportional to the concentration of $\mathbf{Z_5}$.

Reaction	Mechanism	Constants
Set-Point 1	$\varnothing \xrightarrow{\mu_1} \mathbf{Z_1}$	
Set-Point 2	$\varnothing \xrightarrow{\mu_4} \mathbf{Z_4}$	
Sensing 1	$\mathbf{X_L} \xrightarrow{\theta_2} \mathbf{X_L} + \mathbf{Z_2}$	
Sensing 2	$\mathbf{X_L} \xrightarrow{\theta_5} \mathbf{X_L} + \mathbf{Z_5}$	
Conversion 1	$\mathbf{Z_1} \underset{c_{21}}{\overset{c_{11}}{\rightleftharpoons}} \mathbf{Z_4}$	$\rho_1 := \frac{c_{21}}{c_{11}}$
Conversion 2	$\mathbf{Z_2} \underset{c_{22}}{\overset{c_{12}}{\rightleftharpoons}} \mathbf{Z_5}$	$\rho_2 := \frac{c_{22}}{c_{12}}$
Sequestration 1	$\mathbf{Z_1} + \mathbf{Z_2} \xrightarrow{\eta} \mathbf{Z_3}$	
Sequestration 2	$\mathbf{Z_4} + \mathbf{Z_5} \xrightarrow{\eta} \mathbf{Z_3}$	
Sequestration 3	$\mathbf{Z_4} + \mathbf{Z_2} \xrightarrow{\eta} \mathbf{Z_3}$	
Sequestration 4	$\mathbf{Z_1} + \mathbf{Z_5} \xrightarrow{\eta} \mathbf{Z_3}$	
Degradation	$\mathbf{Z_3} \xrightarrow{\delta_0} \varnothing$	
Actuation 1	$\varnothing \xrightarrow{u^+} \mathbf{X_1}$	$u^+ := kz_4$
Actuation 2	$\mathbf{X_1} \xrightarrow{u^-} \varnothing$	$u^- := \gamma z_5$

Supplementary Table 15: List of Reactions

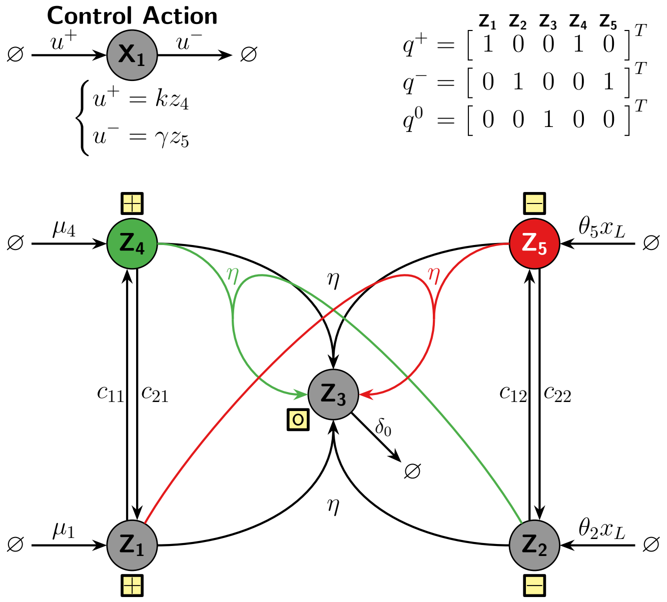

Supplementary Figure 8: Controller network with conversion reactions.

4.2.2 Model Reduction

In this section, we apply Supplementary Theorem 2 to obtain a reduced model for the detailed controller described in Supplementary Table 15. The full controller model is schematically presented in Supplementary Fig. 8 where $\mathbf{Z_4}$ acts as an activator while $\mathbf{Z_5}$ acts as an inhibitor that degrades the input species $\mathbf{X_1}$. These actuation mechanisms are indicated in the actuation functions $u^+ = h^+(z) = kz_4$ and $u^- = h^-(z) = \gamma z_5$, such that the total actuation function is given by

$$u = u^+ - u^- x_1 = kz_4 - \gamma z_5 x_1.$$

Under the assumption that the conversion reactions are fast (i.e. $c_{1i} = \tilde{c}_{1i}/\epsilon, c_{2i} = \tilde{c}_{2i}/\epsilon$, for $i = 1, 2$, as $\epsilon \to 0$), one can easily check that all the conditions of Supplementary Theorem 2 are satisfied using the charge vectors q^+, q^- and q^0 given in Supplementary Fig. 8. Applying Supplementary Theorem 2 yields the following state variables

$$\begin{cases} z^+ &= z_1 + z_4 \\ z^- &= z_2 + z_5 \\ z^0 &= z_3, \end{cases}$$

to arrive at the reduced model given by

$$\begin{cases} \dot{z}^+ &= \mu_1 + \mu_4 - \eta z^+ z^- \\ \dot{z}^- &= (\theta_2 + \theta_5) x_L - \eta z^+ z^- \\ \dot{z}^0 &= \eta z^+ z^- - \delta_0 z^0 \,, \end{cases}$$

since $q^{\pm,0} = \mathbb{1}(q^+ + q^-) \circ q^0 = 0$, and the total control action u is calculated as

$$u = h^+\left(W_1^T z^{\text{tot}} + W_2^T \tilde{z}\right) - h^-\left(W_1^T z^{\text{tot}} + W_2^T \tilde{z}\right) x_1 = h^+\left(\begin{bmatrix} z^+ - \tilde{z}_1 \\ z^- - 2\tilde{z}_2 \\ z^0 \\ \tilde{z}_1 \\ \tilde{z}_2 \end{bmatrix}\right) - h^-\left(\begin{bmatrix} z^+ - \tilde{z}_1 \\ z^- - 2\tilde{z}_2 \\ z^0 \\ \tilde{z}_1 \\ \tilde{z}_2 \end{bmatrix}\right) x_1 = k\tilde{z}_1 - \gamma\tilde{z}_2 x_1,$$

where $\tilde{z} := \begin{bmatrix}\tilde{z}_1 & \tilde{z}_2\end{bmatrix}^T$ is given implicitly by the following algebraic equation

$$\tilde{C} S_C \tilde{\lambda}_C \left(W_1^T z^{\text{tot}} + W_2^T \tilde{z}\right) = 0 \implies \begin{cases} \tilde{c}_{11}\left(z^+ - \tilde{z}_1\right) - \tilde{c}_{21}\tilde{z}_1 = 0 \\ \tilde{c}_{12}\left(z^- - \tilde{z}_2\right) - \tilde{c}_{22}\tilde{z}_2 = 0 \end{cases} \implies \begin{cases} \tilde{z}_1 = \dfrac{z^+}{1+\kappa_1} \\ \tilde{z}_2 = \dfrac{z^-}{1+\kappa_2}. \end{cases} \tag{19}$$

As a result, one can think of the control action u as a function $\mathcal{U}(z^+, z^-)$ defined explicitly as

$$u = \mathcal{U}(z^+, z^-) = \frac{kz^+}{1+\kappa_1} - \frac{\gamma z^- x_1}{1+\kappa_2}.$$

4.2.3 Monotonicity of Actuation

It is straightforward to see that the total control action u is strictly monotonically increasing in z^+ and strictly monotonically decreasing in z^-.

4.2.4 Simulation Results

To demonstrate the accuracy of the model reduction result via simulations, we use the two regulated networks depicted in Supplementary Fig. 9(a) as examples. Regulated Network 1 is comprised of two species $\mathbf{X_1}$ and $\mathbf{X_2}$ and may represent a simple model for gene expression; whereas Regulated Network 2 is more complex and is comprised of six species $\mathbf{X_1}$ through $\mathbf{X_6}$ (taken from [2, 6]). The simulation results for both regulated networks are depicted in Supplementary Fig. 9(c) demonstrating that the reduced model captures the dynamics of the full model to a high degree of accuracy as expected.

4.3 Trimer Controller Circuit

4.3.1 Full Model Description

Consider the various controller reactions listed in Supplementary Table 16 and schematically depicted in Supplementary Fig. 10(a). Observe that, in this example controller, two consecutive binding reactions occur to produce the trimer $\mathbf{Z_6}$.

4.3.2 Model Reduction

In this section, we apply Supplementary Theorem 2 to obtain a reduced model for the detailed controller described in Supplementary Table 16. The full controller model is schematically presented in Supplementary Fig. 10(a) where $\mathbf{Z_6}$ and acts as an activator as indicated in the actuation function $u = h^+(z) = kz_6$. Under the assumption that the dimerization reactions are fast (i.e. $a_i = \tilde{a}_i/\epsilon, d_i = \tilde{d}_i/\epsilon$, for $i = 1, 2, 3$, as $\epsilon \to 0$), one can easily check that all the conditions of Supplementary Theorem 2 are satisfied using the charge vectors q^+, q^- and q^0 given in Supplementary Fig. 10(a). Applying Supplementary Theorem 2 yields the following state variables

$$\begin{cases} z^+ = z_1 + 2z_4 + z_6 \\ z^- = z_2 \\ z^0 = z_3 + 2z_5 + 2z_6, \end{cases}$$

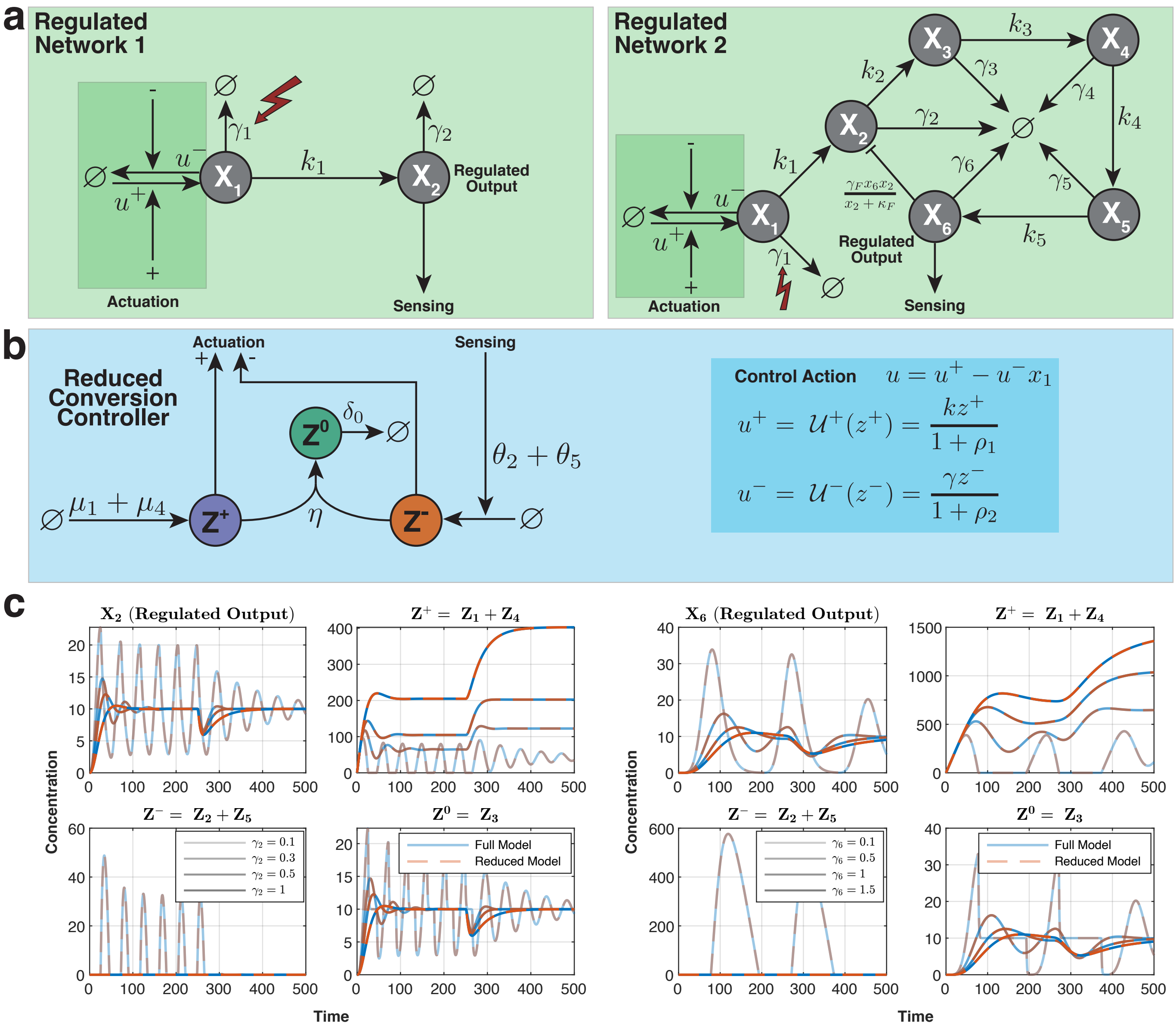

Supplementary Figure 9: Accuracy of the reduced model of the conversion controller. (a) Two test regulated networks. The first regulated network is comprised of two species $\mathbf{X_1}$ and $\mathbf{X_2}$, where $\mathbf{X_2}$ denotes the regulated output. The second regulated network is comprised of six species $\mathbf{X_1}$ through $\mathbf{X_6}$, where $\mathbf{X_6}$ denotes the regulated output. Both regulated networks are actuated by the control action $u = u^+ - u^- x_1$ entering the dynamics as production u^+ and degradation u^- rates of the input species $\mathbf{X_1}$. (b) The reduced model of the conversion controller. The motif of the reduced model is comprised of the three effective species $\mathbf{Z^+}$, $\mathbf{Z^-}$ and $\mathbf{Z^0}$, as per Supplementary Theorem 2. (c) Simulations demonstrating the accuracy of the reduced model of the controller. The simulations are carried out for the closed-loops where the conversion controller is connected in a feedback interconnection with each of the two regulated networks. The simulations are carried out starting from zero initial conditions at $t = 0$ and by doubling the degradation rate γ_1 of the input species as a disturbance at $t = 250$. The simulations are also carried out for four different (initial) values of the degradation rates of the regulated outputs. The simulation results show that the reduced model is capable of accurately capturing the full dynamics of the full controller.
Numerical values. Regulated Network 1: $\gamma_1 = 0.01, k_1 = 1, \mu_1 = \mu_4 = 5, \theta_2 = \theta_5 = 0.5, k = 0.01, \delta_0 = \gamma = 1, \eta = 10, c_{1i} = c_{2i} = 100$ for $i = 1, 2$.
Regulated Network 2: $\gamma_i = k_i = 0.1$ for $i = 1, \cdots, 5$, $\gamma_F = 0.3, \kappa_F = 1, \mu_1 = \mu_4 = 5, \theta_2 = \theta_5 = 0.5, k = 0.05, \delta_0 = 1, \gamma = 5, \eta = 10, c_{1i} = c_{2i} = 100$ for $i = 1, 2$.

to arrive at the reduced model given by

$$\begin{cases} \dot{z}^+ = \mu_1 - \eta z^+ z^- \\ \dot{z}^- = \theta_2 x_L - \eta z^+ z^- \\ \dot{z}^0 = \eta z^+ z^- - \delta_0 z^0 + 2\delta_0 \tilde{z}_3, \end{cases}$$

Reaction	Mechanism	Constants
Set-Point	$\varnothing \xrightarrow{\mu_1} \mathbf{Z_1}$	
Sensing	$\mathbf{X_L} \xrightarrow{\theta_2} \mathbf{X_L} + \mathbf{Z_2}$	
Binding 1	$\mathbf{Z_1} + \mathbf{Z_1} \underset{d_1}{\overset{a_1}{\rightleftharpoons}} \mathbf{Z_4}$	$\kappa_1 := \frac{d_1}{a_1}$
Binding 2	$\mathbf{Z_3} + \mathbf{Z_3} \underset{d_2}{\overset{a_2}{\rightleftharpoons}} \mathbf{Z_5}$	$\kappa_2 := \frac{d_2}{a_2}$
Binding 3	$\mathbf{Z_1} + \mathbf{Z_5} \underset{d_3}{\overset{a_3}{\rightleftharpoons}} \mathbf{Z_6}$	$\kappa_3 := \frac{d_3}{a_3}$
Sequestration 1	$\mathbf{Z_1} + \mathbf{Z_2} \xrightarrow{\eta} \mathbf{Z_3}$	
Sequestration 2	$\mathbf{Z_4} + \mathbf{Z_2} \xrightarrow{2\eta} \mathbf{Z_3} + \mathbf{Z_1}$	
Sequestration 3	$\mathbf{Z_6} + \mathbf{Z_2} \xrightarrow{\eta} \mathbf{Z_3} + \mathbf{Z_5}$	
Degradation	$\mathbf{Z}_i \xrightarrow{\delta_0} \varnothing, \qquad i = 3, 5$	
Actuation	$\varnothing \xrightarrow{u} \mathbf{X_1}$	$u := kz_6$

Supplementary Table 16: List of Reactions

since $\delta_0(q^{\pm,0})^T\psi(z^{\text{tot}}) = 2\delta_0\tilde{z}_3$, and the control action u is calculated as

$$u = h^+\left(W_1^T z^{\text{tot}} + W_2^T \tilde{z}\right) = h^+\left(\begin{bmatrix} z^+ - 2\tilde{z}_1 - \tilde{z}_3 \\ z^- \\ z^0 - 2\tilde{z}_2 - 2\tilde{z}_3 \\ \tilde{z}_1 \\ \tilde{z}_2 \\ \tilde{z}_3 \end{bmatrix}, x_L\right) = k\tilde{z}_3,$$

where $\tilde{z} := \begin{bmatrix}\tilde{z}_1 & \tilde{z}_2 & \tilde{z}_3\end{bmatrix}^T$ is given implicitly by the following algebraic equation

$$\tilde{C}S_B\tilde{\lambda}_B\left(W_1^T z^{\text{tot}} + W_2^T\tilde{z}\right) = 0 \implies \begin{cases} \tilde{a}_1\left(z^+ - 2\tilde{z}_1 - \tilde{z}_3\right)^2 - \tilde{d}_1\tilde{z}_1 = 0 \\ \tilde{a}_2\left(z^0 - 2\tilde{z}_2 - 2\tilde{z}_3\right)^2 - \tilde{d}_2\tilde{z}_2 = 0 \\ \tilde{a}_3\tilde{z}_2(z^+ - 2\tilde{z}_1 - \tilde{z}_3) - \tilde{d}_3\tilde{z}_3 = 0 \end{cases}$$

$$\implies \begin{cases} \left(z^+ - 2\tilde{z}_1 - \tilde{z}_3\right)^2 = \kappa_1\tilde{z}_1 \\ \left(z^0 - 2\tilde{z}_2 - 2\tilde{z}_3\right)^2 = \kappa_2\tilde{z}_2 \\ \tilde{z}_2(z^+ - 2\tilde{z}_1 - \tilde{z}_3) = \kappa_3\tilde{z}_3. \end{cases} \tag{20}$$

As a result, one can think of the control action u as a function $\mathcal{U}(z^+, z^0)$ defined implicitly as

$$u = \mathcal{U}(z^+, z^0) = k\tilde{z}_3,$$

where $\tilde{z}_3$ solves the algebraic equations (20).

Remark 7. *The Differential Algebraic Equations (DAEs) describing the reduced model can be easily solved numerically. In fact, the algebraic equations can be recast as a single sixth order polynomial in $y_2 := \sqrt{\tilde{z}_2}$ as*

$$2\,\kappa_1\,y_2^6 + \kappa_1\sqrt{\kappa_2}\,y_2^5 + \left(-4\,\kappa_3^2 + 2\,\kappa_1\,\kappa_3 - \kappa_1\,z^0 + 2\,\kappa_1\,z^+\right)y_2^4 + \left(\kappa_1\sqrt{\kappa_2}\,\kappa_3 - 4\sqrt{\kappa_2}\,\kappa_3^2\right)y_2^3 + \cdots$$
$$\cdots + \left(4\,\kappa_3^2\,z^0 - \kappa_2\,\kappa_3^2 - \kappa_1\,\kappa_3\,z^0\right)y_2^2 + \left(2\sqrt{\kappa_2}\,\kappa_3^2\,z^0\right)y_2 - (\kappa_3 z^0)^2 = 0,$$

which can be numerically solved in MATLAB using the command "roots" within each iteration of an ODE solver without having to provide an initial guess. This gives us $\tilde{z}_2$. The other two variables $\tilde{z}_1$ and $\tilde{z}_3$ can thus be calculated as

$$\tilde{z}_1 = \frac{\kappa_3^2\left(\sqrt{\kappa_2\tilde{z}_2} - z^0 + 2\tilde{z}_2\right)^2}{4\kappa_1\tilde{z}_2^2} \quad \textit{and} \quad \tilde{z}_3 = \frac{\tilde{z}_2}{\kappa_3}\sqrt{\kappa_1 * \tilde{z}_1}.$$

4.3.3 Monotonicity of Actuation

Next, we study the monotonicity of the control action u with respect to z^+ and z^0 , that is the monotonicity of the actuation function $\mathcal{U}$. For convenience, we rewrite the three equations in (20) as

$$\begin{cases} 2\tilde{z}_1 + \tilde{z}_3 + \sqrt{\kappa_1 \tilde{z}_1} = z^+ \\ 2\tilde{z}_2 + 2\tilde{z}_3 + \sqrt{\kappa_2 \tilde{z}_2} = z^0 \\ -\kappa_3 \tilde{z}_3 + \tilde{z}_2 \sqrt{\kappa_1 \tilde{z}_1} = 0. \end{cases}$$

By differentiating the three equations with respect to z^+, we obtain the following linear system of equations

$$\underbrace{\begin{bmatrix} 2 + \frac{1}{2}\sqrt{\frac{\kappa_1}{\tilde{z}_1}} & 0 & 1 \\ 0 & 2 + \frac{1}{2}\sqrt{\frac{\kappa_2}{\tilde{z}_2}} & 2 \\ \frac{\tilde{z}_2}{2}\sqrt{\frac{\kappa_1}{\tilde{z}_1}} & \sqrt{\kappa_1 \tilde{z}_1} & -\kappa_3 \end{bmatrix}}_{A} \begin{bmatrix} \frac{\partial \tilde{z}_1}{\partial z^+} \\ \frac{\partial \tilde{z}_2}{\partial z^+} \\ \frac{\partial \tilde{z}_3}{\partial z^+} \end{bmatrix} = \underbrace{\begin{bmatrix} 1 \\ 0 \\ 0 \end{bmatrix}}_{b^+}.$$

Then by Cramer's rule we obtain

$$\frac{\partial \tilde{z}_1}{\partial z^+} = -\frac{\kappa_3\left(2 + \frac{1}{2}\sqrt{\frac{\kappa_2}{\tilde{z}_2}}\right) + 2\sqrt{\kappa_1 \tilde{z}_1}}{\det(A)}, \qquad \frac{\partial \tilde{z}_2}{\partial z^+} = \frac{\tilde{z}_2 \sqrt{\frac{\kappa_1}{\tilde{z}_1}}}{\det(A)}, \qquad \frac{\partial \tilde{z}_3}{\partial z^+} = -\frac{\frac{\tilde{z}_2}{2}\sqrt{\frac{\kappa_1}{\tilde{z}_1}}\left(2 + \frac{1}{2}\sqrt{\frac{\kappa_2}{\tilde{z}_2}}\right)}{\det(A)},$$

where

$$\det(A) = -\left(2 + \frac{1}{2}\sqrt{\frac{\kappa_1}{\tilde{z}_1}}\right)\left[\kappa_3\left(2 + \frac{1}{2}\sqrt{\frac{\kappa_2}{\tilde{z}_2}}\right) + 2\sqrt{\kappa_1 \tilde{z}_1}\right] - \frac{\tilde{z}_2}{2}\sqrt{\frac{\kappa_1}{\tilde{z}_1}}\left(2 + \frac{1}{2}\sqrt{\frac{\kappa_2}{\tilde{z}_2}}\right) < 0.$$

Hence, we have

$$\frac{\partial \tilde{z}_1}{\partial z^+} > 0, \qquad \frac{\partial \tilde{z}_2}{\partial z^+} < 0, \qquad \text{and} \qquad \frac{\partial \tilde{z}_3}{\partial z^+} > 0 \qquad \Longrightarrow \frac{\partial \mathcal{U}(z^+, z^0)}{\partial z^+} > 0.$$

This means that the control action $u = \mathcal{U}(z^+, z^0)$ is a strictly monotonically increasing function of z^+.

Next, by differentiating the three equations with respect to z^0 , we obtain the following linear system of equations

$$\underbrace{\begin{bmatrix} 2 + \frac{1}{2}\sqrt{\frac{\kappa_1}{\tilde{z}_1}} & 0 & 1 \\ 0 & 2 + \frac{1}{2}\sqrt{\frac{\kappa_2}{\tilde{z}_2}} & 2 \\ \frac{\tilde{z}_2}{2}\sqrt{\frac{\kappa_1}{\tilde{z}_1}} & \sqrt{\kappa_1 \tilde{z}_1} & -\kappa_3 \end{bmatrix}}_{A} \begin{bmatrix} \frac{\partial \tilde{z}_1}{\partial z^0} \\ \frac{\partial \tilde{z}_2}{\partial z^0} \\ \frac{\partial \tilde{z}_3}{\partial z^0} \end{bmatrix} = \underbrace{\begin{bmatrix} 0 \\ 1 \\ 0 \end{bmatrix}}_{b^0}.$$

Then by Cramer's rule we obtain

$$\frac{\partial \tilde{z}_1}{\partial z^0} = \frac{\sqrt{\kappa_1 \tilde{z}_1}}{\det(A)}, \qquad \frac{\partial \tilde{z}_2}{\partial z^0} = -\frac{\kappa_3\left(2 + \frac{1}{2}\sqrt{\frac{\kappa_1}{\tilde{z}_1}}\right) + \frac{\tilde{z}_2}{2}\sqrt{\frac{\kappa_1}{\tilde{z}_1}}}{\det(A)}, \qquad \frac{\partial \tilde{z}_3}{\partial z^0} = -\frac{\frac{1}{2}\sqrt{\kappa_1 \tilde{z}_1}\left(2 + \frac{1}{2}\sqrt{\frac{\kappa_1}{\tilde{z}_1}}\right)}{\det(A)}.$$

Hence, we have

$$\frac{\partial \tilde{z}_1}{\partial z^0} < 0, \qquad \frac{\partial \tilde{z}_2}{\partial z^0} > 0, \qquad \text{and} \qquad \frac{\partial \tilde{z}_3}{\partial z^0} < 0 \qquad \Longrightarrow \frac{\partial \mathcal{U}(z^+, z^0)}{\partial z^0} < 0.$$

This means that the control action $u = \mathcal{U}(z^+, z^0)$ is a strictly monotonically decreasing function of z^0 .

4.3.4 Simulation Results

To demonstrate the accuracy of the model reduction result via simulations, we use the regulated network depicted in Supplementary Fig. 10(a) as an example. The regulated Network is comprised of two species $\mathbf{X_1}$ and $\mathbf{X_2}$ and may represent a simple model for gene expression. The simulation results are depicted in Supplementary Fig. 10(c) demonstrating that the reduced model captures the dynamics of the full model to a high degree of accuracy as expected.

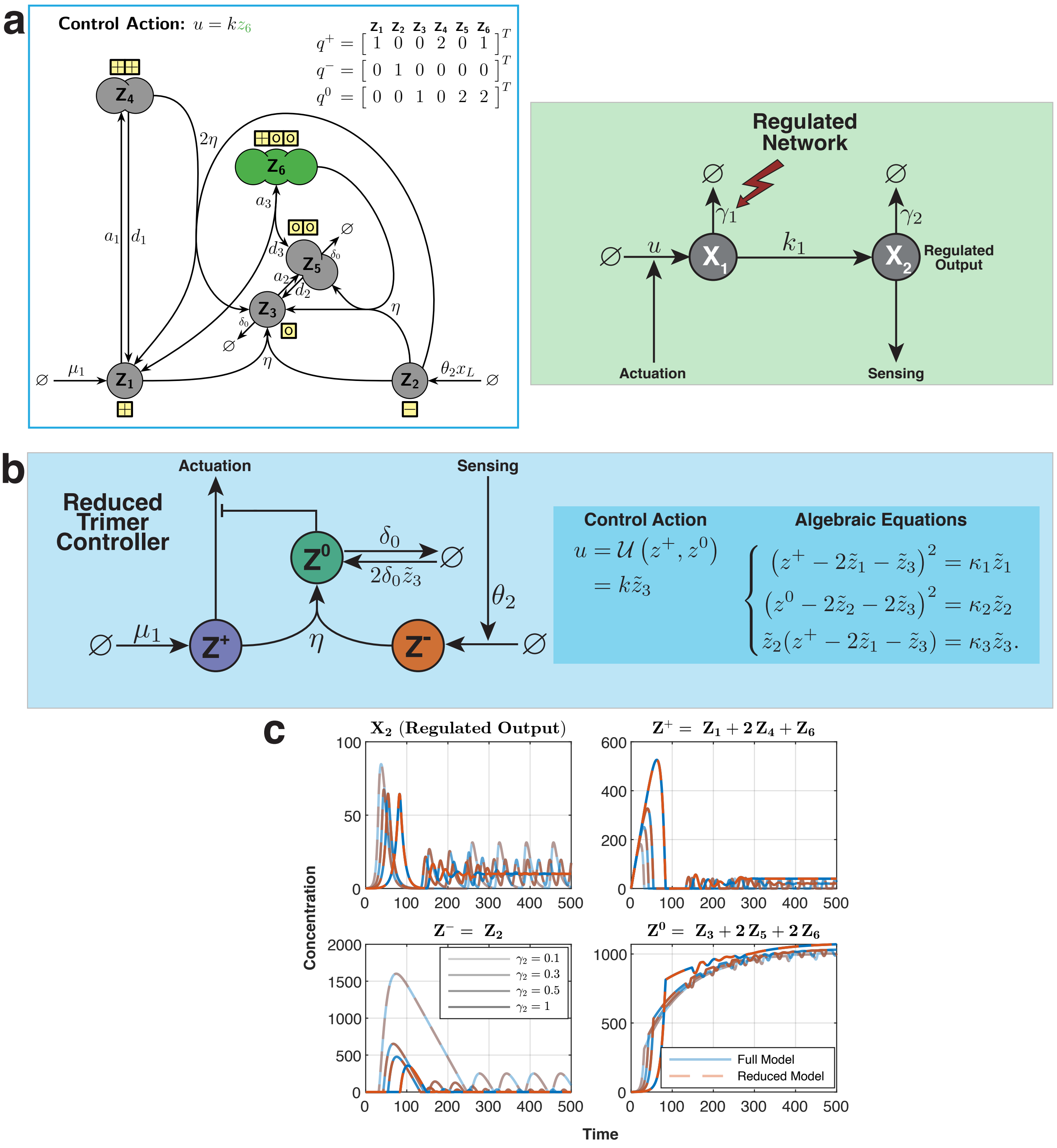

Supplementary Figure 10: Accuracy of the reduced model of the Trimer controller. (a) The full controller model and an example regulated network. The regulated network is comprised of two species $\mathbf{X_1}$ and $\mathbf{X_2}$, where $\mathbf{X_2}$ denotes the regulated output. The regulated network is actuated by the control action u entering the dynamics as a production rate of the input species $\mathbf{X_1}$. (b) The reduced model of the Trimer controller. The motif of the reduced model is comprised of the three effective species $\mathbf{Z^+}$, $\mathbf{Z^-}$ and $\mathbf{Z^0}$, as per Supplementary Theorem 2, coupled to the three algebraic equations in $\tilde{z}_1, \tilde{z}_2$ and $\tilde{z}_3$. (c) Simulations demonstrating the accuracy of the reduced model of the controller. The simulations are carried out for the closed-loops where the Trimer controller is connected in a feedback interconnection with the regulated network. The simulations are carried out by doubling the degradation rate of the regulated outputs as a disturbance at $t = 250$. The simulations are also carried out for four different (initial) values of the degradation rates of the regulated outputs. The simulation results show that the reduced model is capable of accurately capturing the full dynamics of the full controller.
Numerical values. Regulated Network 1: $\gamma_1 = 0.1, k_1 = 1, k = 0.05, \delta_0 = 0.01, \theta_2 = 1, \mu_1 = \eta = 10, a_i = d_i = 100$ for $i = 1, 2, 3$.

5 Design & Implementation Flexibility of Inteins: Examples of Intein-based Integral Controller Circuits

In this section, we highlight the exquisite flexibility offered by inteins to design integral controllers that enable RPA. We do so, by proposing a collection of controllers that are not limited only to transcription factors, but also proteases and receptors. We also demonstrate the advantage of fusing multiple inteins sequentially in a single gene.

5.1 Integral Controllers Based on Transcription Factors & Proteases

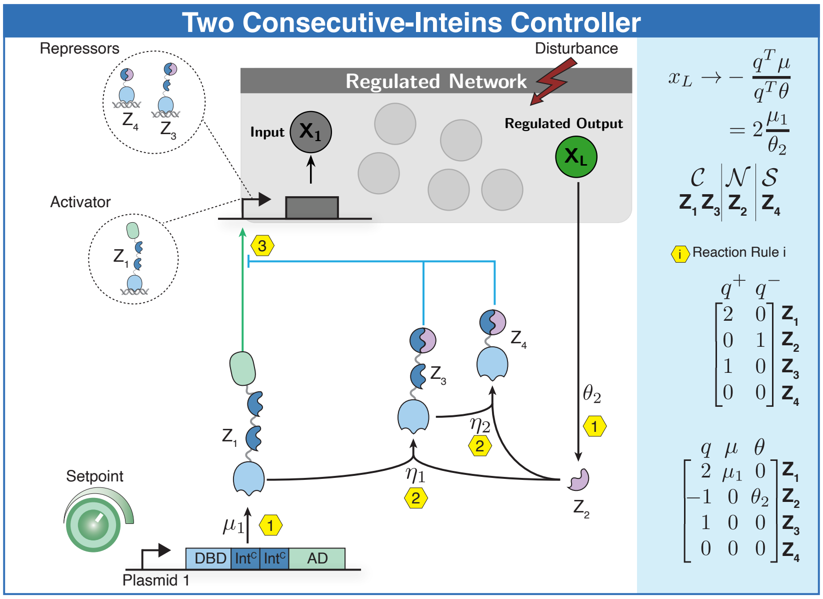

Supplementary Figure 11: This controller example illustrates that it is possible to use two consecutive inteins within the same protein which leads to two sequential intein-splicing (sequestration) reactions. This has the effect of doubling the setpoint to yield $2\mu_1/\theta_2$ which can be shown by a direct application of Theorem 1. Note that, for simplicity the first sequestration is shown with the C-terminal Int^{C}; however, the first splicing can happen with either of the two Int^{C}, which leads to a different set of products.

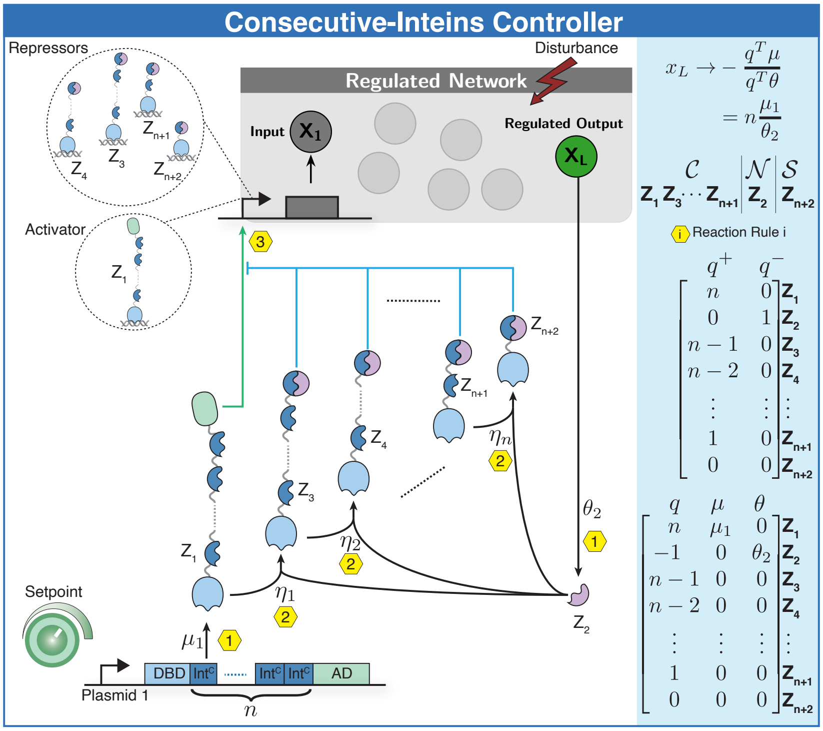

Supplementary Figure 12: This controller example illustrates that the controller circuit presented in Supplementary Fig. 11 can be generalized to multiple inteins with $n = 2, 3, \cdots$. This has the effect of multiplying the setpoint by n to yield $n\mu_1/\theta_2$ which, once again, can be shown by a direct application of Theorem 1. Note that, for simplicity the first sequestration is shown with the C-terminal $\mathrm{Int}^{\mathrm{C}}$; however, the first splicing can happen with either of the n $\mathrm{Int}^{\mathrm{C}}$, which leads to a different set of products.

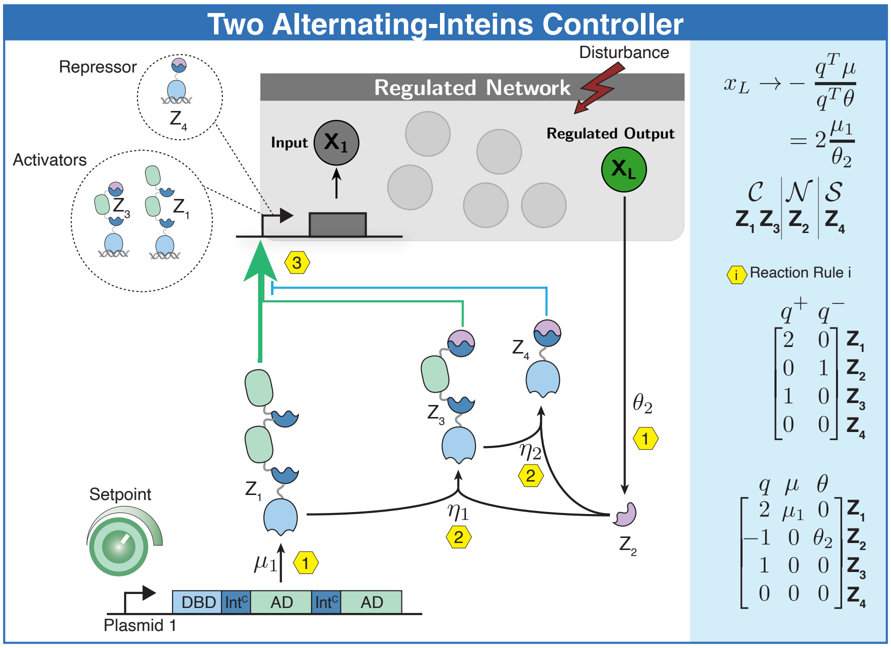

Supplementary Figure 13: This controller example illustrates that it is possible to use multiple inteins within the same protein, which are separated by additional protein domains. The positioning, the effector domains and the order of splicing can lead to a variety of effectors and actuation mechanisms. Note that the spliced products have different activities in comparison to the example in Supplementary Fig. 11; nonetheless, the setpoint is also doubled and is given by $2\mu_1\theta_2$. For simplicity the first sequestration is shown with the C-terminal $\mathrm{Int}^{\mathrm{C}}$; however, the first splicing can happen with either of the two $\mathrm{Int}^{\mathrm{C}}$, which will lead to a different set of products.

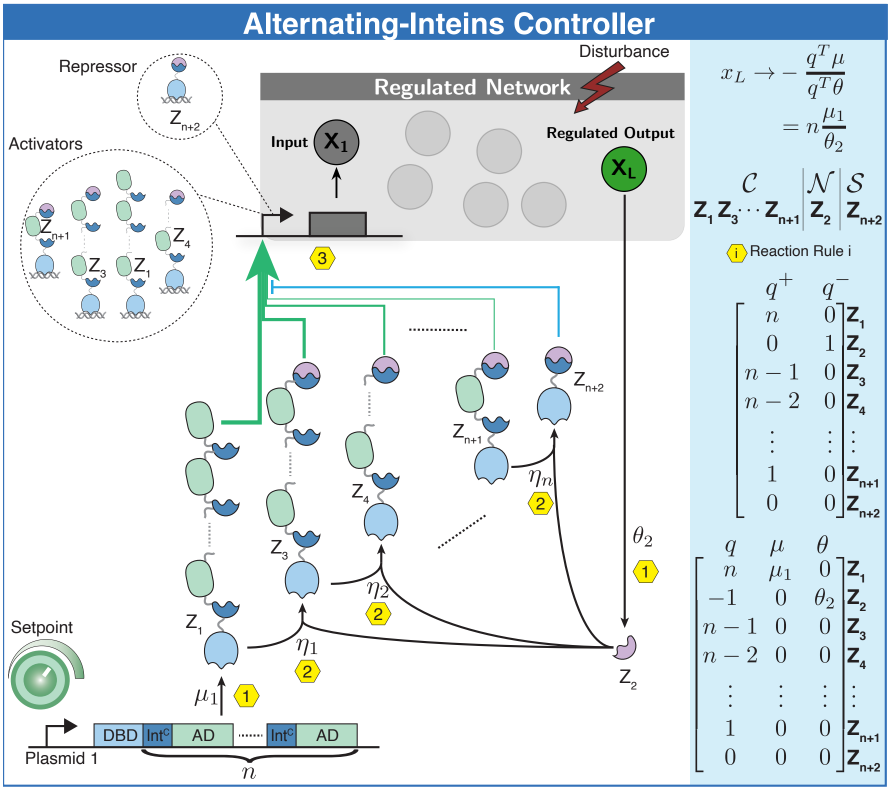

Supplementary Figure 14: This controller example illustrates that the controller circuit presented in Supplementary Fig. 13 can be generalized to multiple inteins with $n = 2, 3, \cdots$. This has the effect of multiplying the setpoint by n to yield $n\mu_1/\theta_2$ which, once again, can be shown by a direct application of Theorem 1. Note that, for simplicity the first sequestration is shown with the C-terminal Int^{C}; however, the first splicing can happen with either of the n Int^{C}, which leads to a different set of products.

5.2 Integral Controllers Based on Receptors

5.2.1 Full Model Description

Consider the receptor-based controller network depicted schematically in Supplementary Fig. 19, where the details of the various controller species and reactions are listed in Supplementary Tables 17 and 18, respectively.

Species	Symbol	Species	Symbol
ECD:TMD:Int^{C}:STD	$\mathbf{Z_1}$	$\mathbf{Z_1}$:$\mathbf{Z_1}$	$\mathbf{Z_5}$
Int^{N}	$\mathbf{Z_2}$	ECD:TMD:Int^{C}:Int^{N}	$\mathbf{Z_3}$
$\mathbf{Z_3}$:$\mathbf{Z_3}$	$\mathbf{Z_4}$	$\mathbf{Z_3}$:$\mathbf{Z_1}$	$\mathbf{Z_6}$

Supplementary Table 17: List of Biochemical Species

Observe that the mathematical model of this controller is almost exactly the same as the Gal4 controller presented in Section 3.2. The only difference is that the dimerization (or binding) reactions are controlled by an extra parameter w that reflects the concentration of the ligands or light intensity in the case of light-based receptors. Furthermore, the dissociation constants κ for all of the three binding reactions of Supplementary Table 18 are assumed to be equal.

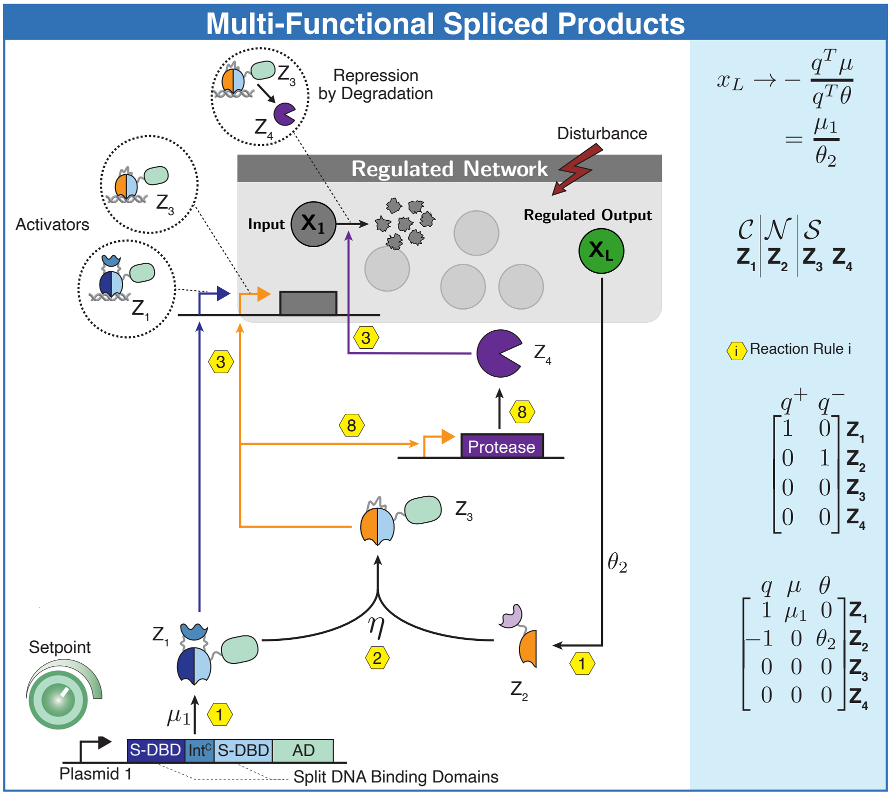

Supplementary Figure 15: This controller example illustrates that the splicing product can simultaneously positively and negatively actuate the regulated network. The controller species $\mathbf{Z_1}$ consists of a dimer-dependent transcription factor [7], which are held together by an $\mathrm{Int}^{\mathrm{C}}$ and an AD. The splicing reaction exchanges part of the DBD domain, which alters the binding affinity to the promoter. The newly formed activator actuates the network through a separate orthogonal promoter and also produces a protease that degrades the input species. The splicing product contributes to the dynamics via an incoherent feedforward loop due to the delayed production of the protease.

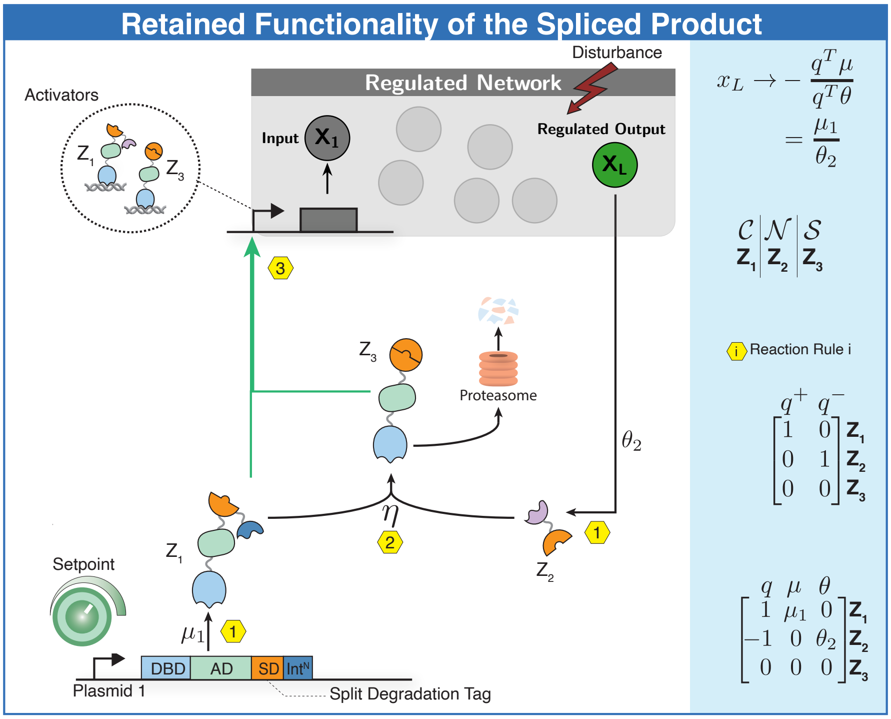

Supplementary Figure 16: This controller example illustrates that the splicing product may still retain the original function of its educts. Here we have a TF with a split degradation tag, which is by itself inactive. Upon the splicing reaction, the full length degradation tag is restored leading to a higher degradation rate of the TF. However, until the protein gets degraded it will still positively actuate the plant.

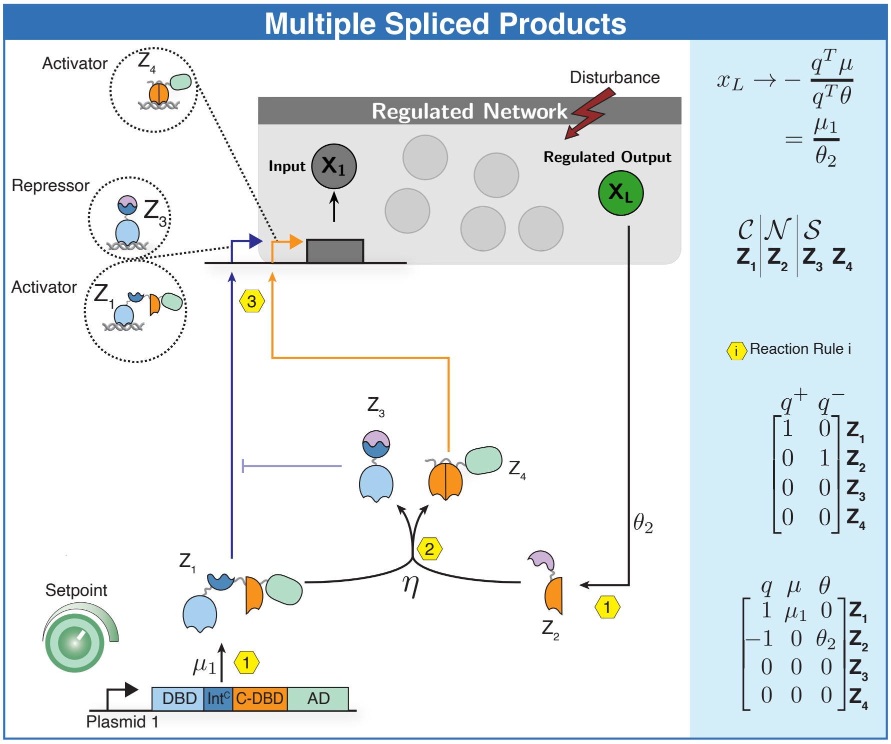

Supplementary Figure 17: This controller example demonstrates that the intein-splicing reaction can be engineered to produce multiple products, both of which have their own functionalities. In all the other examples of this section, we have only tracked one of the products as the other one was non-functional. In this example, we have a DBD domain, which is linked to a split DBD and an AD via an Int^{C}. The splicing reaction detaches the full length DBD, which now acts as a repressor through competition. Furthermore it reconstitutes the two split DBD, which now acts as an activator due to the AD. This results in two functional products: a transcriptional repressor and a transcriptional activator.

Reaction	Mechanism	Constants
Set-Point	$\varnothing \xrightarrow{\mu_1} \mathbf{Z_1}$	
Sensing	$\mathbf{X_L} \xrightarrow{\theta_2} \mathbf{X_L} + \mathbf{Z_2}$	
Binding 1	$\mathbf{Z_1} + \mathbf{Z_1} \underset{d}{\overset{wa}{\rightleftharpoons}} \mathbf{Z_5}$	$\kappa(w) := \frac{d}{wa}$
Binding 2	$\mathbf{Z_1} + \mathbf{Z_3} \underset{d}{\overset{wa}{\rightleftharpoons}} \mathbf{Z_6}$	$\kappa(w) := \frac{d}{wa}$
Binding 3	$\mathbf{Z_3} + \mathbf{Z_3} \underset{d}{\overset{wa}{\rightleftharpoons}} \mathbf{Z_4}$	$\kappa(w) := \frac{d}{wa}$
Intein-Splicing 1	$\mathbf{Z_1} + \mathbf{Z_2} \xrightarrow{\eta} \mathbf{Z_3}$	
Intein-Splicing 2	$\mathbf{Z_5} + \mathbf{Z_2} \xrightarrow{2\eta} \mathbf{Z_6}$	
Intein-Splicing 3	$\mathbf{Z_6} + \mathbf{Z_2} \xrightarrow{\eta} \mathbf{Z_4}$	
Degradation	$\mathbf{Z}_i \xrightarrow{\delta_0} \varnothing, \qquad i = 3, 4$	
Dilution	$\mathbf{Z}_i \xrightarrow{\delta} \varnothing, \qquad i = 1, \cdots, 6$	
Actuation	$\varnothing \xrightarrow{u} \mathbf{X_1}$	$u := kz_5$

Supplementary Table 18: List of Biochemical Reactions

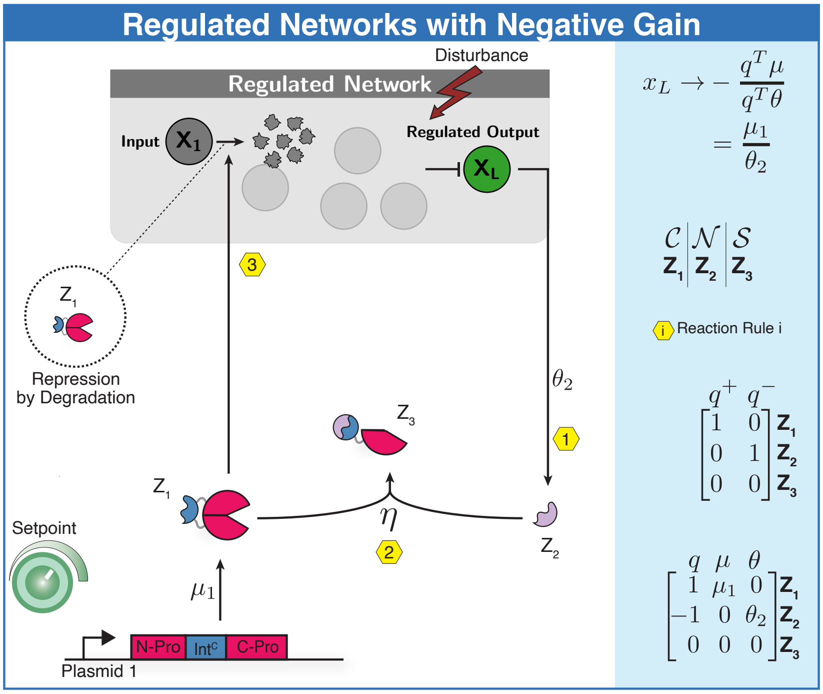

Supplementary Figure 18: In all the controller circuits presented so far, we have only considered regulated networks with positive gains, that is producing more input species $\mathbf{X_1}$ leads to producing more output species $\mathbf{X_L}$. To favor closed-loop stability, such regulated networks require controllers to exert positive actuation by$\mathbf{Z_1}$ in order to implement an overall negative feedback. However, exerted negative actuation by $\mathbf{Z_1}$ is favored for closed-loop stability if the regulated network has a negative gain, that is producing more input species $\mathbf{X_1}$ leads to producing less output species $\mathbf{X_L}$ (see P-Type and N-Type controllers in [2]. This negative actuation can be simply achieved by introducing an intein in a repressor or in this example a protease (Pro). A split protease is connected here with a linker containing an Int^{N}. Upon splicing the linker is cut and the protease is rendered non-functional.

5.2.2 Model Reduction

Since the mathematical model of the receptor-based controller is the same as that of the Gal4 controller presented in Section 3.2, the reduced model is also the same and is given by

$$\begin{aligned}
&\text{Differential Equations:} && \begin{cases} \dot{z}^+ = \mu_1 - \eta z^+ z^- - \delta z^+ \\ \dot{z}^- = \theta_2 x_L - \eta z^+ z^- - \delta z^- \\ \dot{z}^0 = \eta z^+ z^- - (\delta + \delta_0) z^0 + \delta_0 \tilde{z}_3 \end{cases} \\
&\text{Control Action:} && u = \mathcal{U}(z^+, z^0; w) = k\tilde{z}_2 \\
&\text{Algebraic Equations:} && \begin{cases} \left(z^0 - 2\tilde{z}_1 - \tilde{z}_3\right)^2 = \kappa(w)\tilde{z}_1 \\ \left(z^+ - 2\tilde{z}_2 - \tilde{z}_3\right)^2 = \kappa(w)\tilde{z}_2 \\ \tilde{z}_1\tilde{z}_2 = \tilde{z}_3^2. \end{cases}
\end{aligned} \tag{21}$$

5.2.3 Monotonicity of Actuation

Once again, since the reduced model here is the same as that of the Gal4 controller presented in Section 3.2, then the monotonicities of the control action with respect to the effective species concentrations z^+ and z^0 are the same.

Next, we establish that the control action is a monotonically increasing function of w which acts as a tuning knob for the controller gain. For convenience, we rewrite the three equations in (21) as

$$\begin{cases} 2\tilde{z}_1 + \sqrt{\tilde{z}_1\tilde{z}_2} + \sqrt{\kappa(w)\tilde{z}_1} = z^0 \\ 2\tilde{z}_2 + \sqrt{\tilde{z}_1\tilde{z}_2} + \sqrt{\kappa(w)\tilde{z}_2} = z^+. \end{cases} \tag{22}$$

The goal is to examine the sign of the $\frac{\partial u}{\partial w} = k\frac{\partial \tilde{z}_2}{\partial w}$. To simplify the analysis, we define

$$y_i := \sqrt{\tilde{z}_i} \quad \text{and} \quad \alpha(w) := \sqrt{\kappa(w)}, \quad \text{for} \quad i = 1, 2,$$

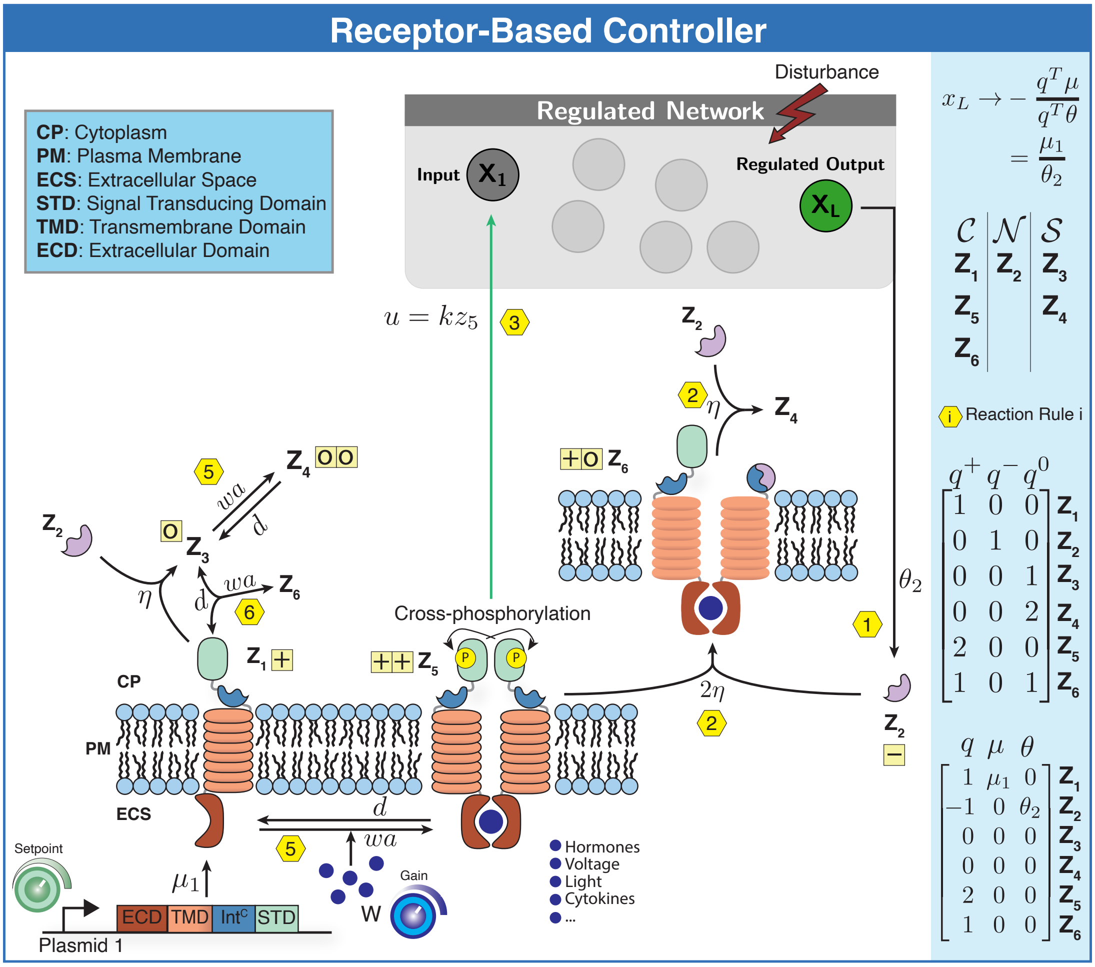

Supplementary Figure 19: This example illustrates the flexibility of inteins in converting different types of proteins into controller species that realize integral control. The example here is based on a generic receptor consisting of an extra cellular domain (ECD), a transmembrane domain (TMD) and a signal transducing domain (STD). The intein is inserted in between the TMD and STD and the entire construct is constitutively expressed. The ligand concentration is sensed by the receptor. Upon ligand binding the receptor dimerizes and leads to a cross phosphorylation of the STD, which eventually actuates the regulated network through a cascade of protein-protein interactions. The splicing reactions removes the STD domain from the remaining receptor. The truncated receptor dimer is still able to bind to the ligand but it is unable to actuate the network. Note that the ligands do not have to be restricted to be physical molecules such as hormones or cytokine, but also the dimerization of the receptor can also be induced through light or voltage.

to rewrite (22) as

$$\begin{cases} 2y_1^2 + y_1 y_2 + \alpha(w) y_1 = z^0 \\ 2y_2^2 + y_1 y_2 + \alpha(w) y_2 = z^+. \end{cases} \tag{23}$$

Then we have $\frac{\partial u}{\partial w} = 2ky_2 \frac{\partial y_2}{\partial w}$ and thus $\frac{\partial u}{\partial w}$ takes the sign of $\frac{\partial y_2}{\partial w}$ which can be calculated by differentiating the two equations in (23) with respect to w to obtain the following linear system of equations

$$\begin{bmatrix} 4y_1 + y_2 + \alpha(w) & y_1 \\ y_2 & 4y_2 + y_1 + \alpha(w) \end{bmatrix} \begin{bmatrix} \frac{\partial y_1}{\partial w} \\ \frac{\partial y_2}{\partial w} \end{bmatrix} = \begin{bmatrix} \alpha'(w) y_1 \\ \alpha'(w) y_2 \end{bmatrix} \implies \frac{\partial y_2}{\partial w} = -\frac{\alpha'(w) y_2 (\alpha(w) + 3y_1 + y_2)}{\alpha^2(w) + 5\alpha(w)(y_1 + y_2) + 4(y_1 + y_2)^2 + 8y_1 y_2}.$$

By observing that $\alpha'(w) < 0$, we conclude that $\frac{\partial y_2}{\partial w} \leq 0$, and thus the control action is monotonically increasing in w.

5.2.4 Simulation Results

In this section, we demonstrate, via numerical simulations, the accuracy of the model reduction and the effect of w on tuning the controller gain. To do se, we use the regulated network depicted in Supplementary Fig. 20(a) as an example. Here, the regulated network is comprised of two species $\mathbf{X_1}$ and $\mathbf{X_2}$ and may represent a simple model for gene expression. The simulation results for both regulated networks are depicted in Supplementary Fig. 20(b) for increasing values of w. The results demonstrate that the reduced model captures the dynamics of the full model to a high degree of accuracy as expected. They time responses along with the control maps shown in Supplementary Fig. 20(c) for the five different values of w illustrate that increasing w increases the controller gain and thus speeds up the response.

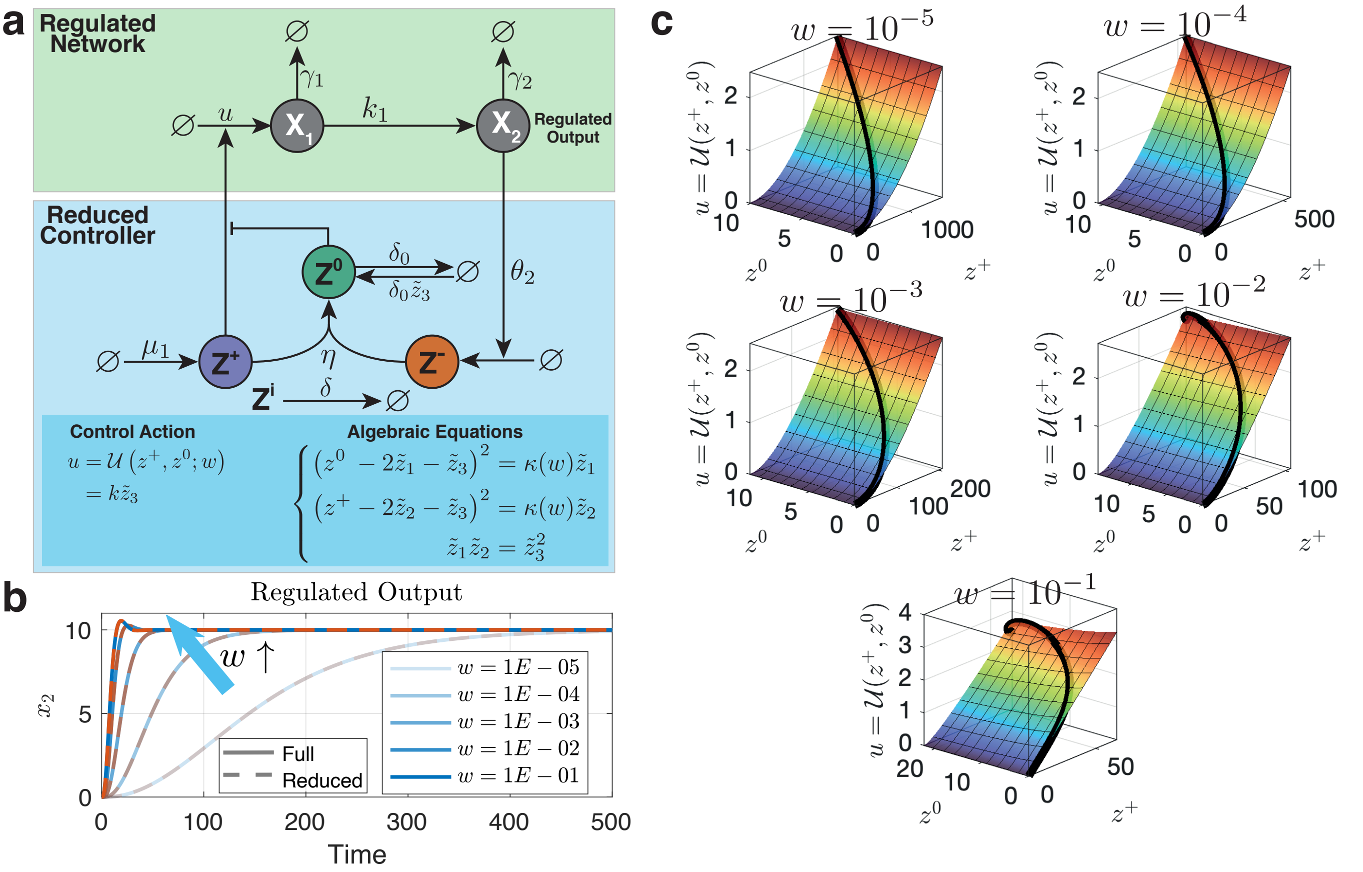

Supplementary Figure 20: The receptor-based controller. (a) The reduced model of the receptor-based controller connected in a feedback interconnection with a regulated network comprised of two species $\mathbf{X_1}$ and $\mathbf{X_2}$ as an example, where $\mathbf{X_2}$ is the regulated output. (b) Simulation of the closed-loop dynamics for different values of w. The concentration response of the regulated output is plotted for $w \in \{10^-5, 10^{-4}, 10^{-3}, 10^{-2}, 10^{-1}\}$. The parameter w, which reflects the concentration of a ligand, or light intensity in the case of light-based receptors, can be conveniently tuned to increase the controller gain and thus speed up the response. (c) The control map $u = \mathcal{U}(z^+, z^0\ ; w)$ plotted as a function of z^+ and z^0 for the various values of w used for the numerical simulations in panel b. The solid black line shows how the control action evolves for each of the associated simulations shown in panel b. The control map is strictly monotonically increasing in z^+ and strictly monotonically decreasing in z^0. As w is increased, the control map becomes more aggressive. Observe that for all five values of w, the steady-state control action $\bar{u} = 2.5$ is the same. The difference between the control actions in the five cases is how the control action evolves transiently on the control map to reach the steady-state value. The aggressiveness of the control map for higher values of w allows the control action to increase more aggressively towards the steady-state value. This leads to speeding up the regulated output response as demonstrated in panel b.
Numerical values. $k_1 = 1, \gamma_1 = \gamma_2 = 0.5, \theta_2 = \delta_0 = 1, \kappa_1 = \kappa_2 = \kappa_3 = 1, \mu_1 = \eta = 10, k = 0.1, \delta = 0.$

6 A Case Study: Model Reduction in the Stochastic Setting

Although the RPA result presented in Supplementary Theorem 1 extends to the stochastic setting, it remains unclear whether the model reduction result presented in Supplementary Theorem 2 extends to the stochastic setting. To rigorously establish such a result, a different set of mathematical tools, compared to those used for the deterministic setting, need to be exploited here. This is left for future work; however, in this section, we perform a preliminary simulation case-study for one controller, as an example, to briefly explore our developed model reduction technique in the stochastic setting.

Consider the intraDD controller, shown in Fig. 3, which is thoroughly studied in Section 3.3 in the deterministic setting. The controller circuit is now considered in the stochastic setting and is repeated here in Supplementary Fig. 21(a) for convenience. The general set of reactions can be found in Supplementary Table 9. The only difference with the deterministic setting is the propensity function of the homodimerization reaction given by

$$\mathrm{Z}_1 + \mathrm{Z}_1 \underset{d}{\overset{a}{\rightleftharpoons}} \mathrm{Z}_4 \qquad \text{with propensities:} \qquad \begin{cases} \text{Deterministic Setting:} & \left(az_1^2, dz_4\right) \\ \text{Stochastic Setting:} & \left(a\dfrac{z_1(z_1-1)}{2}, dz_4\right). \end{cases}$$

By replacing the deterministic propensity function of the homodimerization reaction with its stochastic counterpart and repeating the calculations carried out in Section 3.3.2, we obtain the same reduced model in the stochastic setting but with a slightly different reduced control action u shown in Supplementary Fig. 21(b).

To test the validity of the reduced stochastic model, we carry out stochastic simulations [8] to generate 10^5 stochastic trajectories for both the full and reduced models of the controller regulating the gene expression network depicted in Supplementary Fig. 21(c) (top left). The mean dynamics, variance dynamics and stationary distribution of the regulated output $\mathbf{X_2}$ are empirically estimated from the generated trajectories, for the full and reduced models, and are depicted in Supplementary Fig. 21(c). The simulations demonstrate that that the reduced model accurately captures the aforementioned attributes of the full model. This suggests that the model reduction approach seems to be possibly extendable to the stochastic setting. Of course, this remains a speculation based on simulations and is yet to be rigorously established.

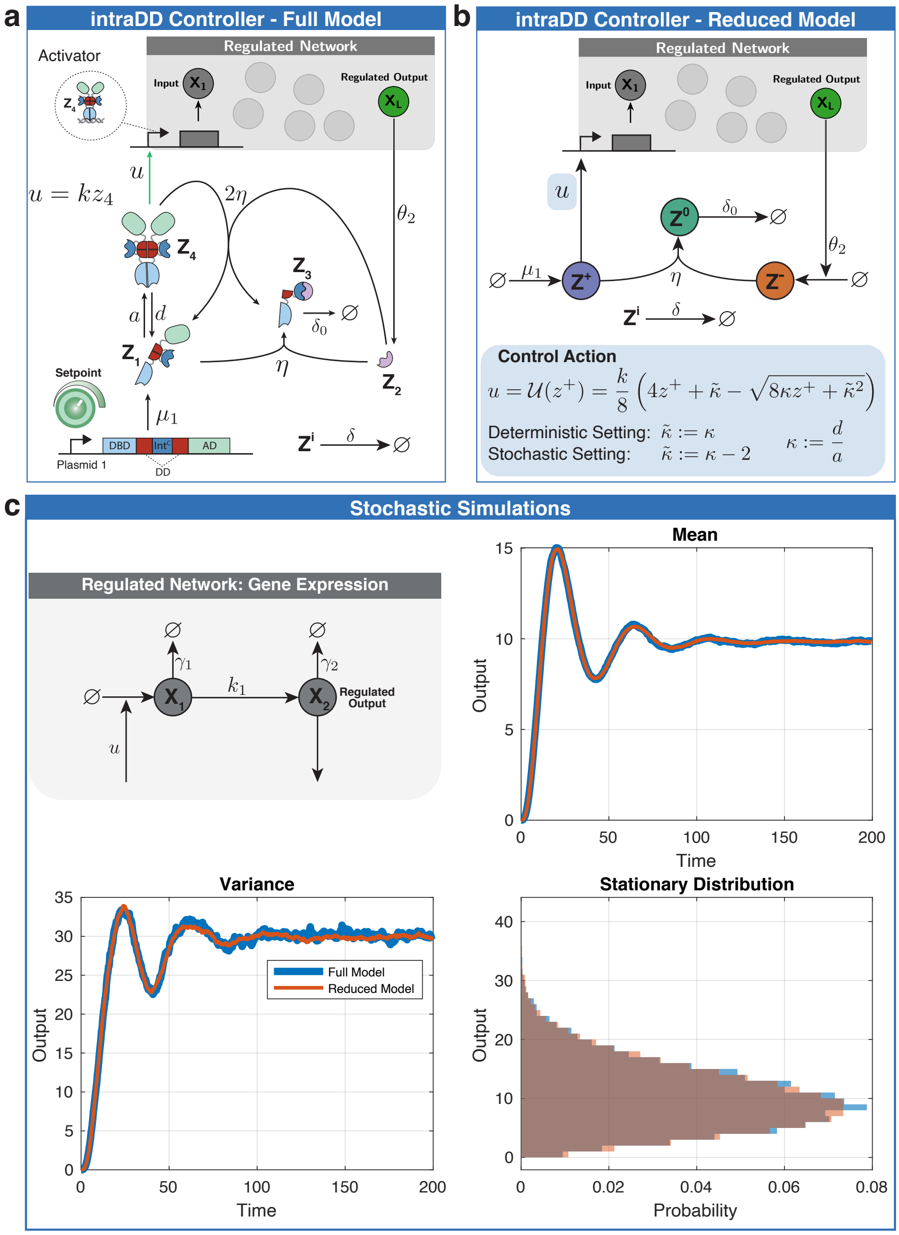

Supplementary Figure 21: Stochastic simulations of the intraDD controller. (a) The full model of the intraDD controller. The controller circuit is the same as that shown in Fig. 3; however, the stochastic setting is considered here. Since the RPA result presented in Theorem 1 extends to the stochastic setting, this controller in the ideal case ($\delta = 0$) ensures RPA under the assumption that the closed-loop network is ergodic. In fact, the setpoint is given by $\mathbb{E}_\pi [X_2] = \mu_1/\theta_2$. Observe that a degradation reaction for $\mathbf{Z_3}$ is added here, compared to Fig. 3, to consider the more general case studied in Supplementary Table 9. (b) The reduced model of the intraDD controller operating in the stochastic setting. Compared to the deterministic setting (see Supplementary Fig. 6(b)), the reduced model in the stochastic setting has only one difference that appears in the reduced control action u. This difference stems from the fact that the functional forms of the mass-action propensity of homodimerization reactions are slightly different between deterministic and stochastic settings. (c) Stochastic simulation results. To perform stochastic simulations for the full and reduced models, we consider a particular regulated network as an example. The regulated network here is comprised of two species $\mathbf{X_1}$ and $\mathbf{X_2}$ such that $\mathbf{X_2}$ is catalytically produced by $\mathbf{X_1}$ at a rate k_1, and both species degrade at a rate γ_1 and γ_2. The regulated network is actuated by producing $\mathbf{X_1}$ at a rate u which is the control action and the regulated output is $\mathbf{X_2}$ that is sensed by the controller. This regulated network can be thought of as a simple model for gene expression where $\mathbf{X_1}$ models the mRNA while $\mathbf{X_2}$ models the associated protein. The stochastic simulation algorithm [8] is used here to generate 10^5 stochastic trajectories for both the full and reduced models. The mean (population) dynamics, variance dynamics and stationary distribution of the regulated output $\mathbf{X_2}$ are then empirically approximated from the generated trajectories for both models (full model:blue, reduced model: red). This simulation study suggests that the reduced model accurately captures the features of the full model, while reducing the simulation time from around 40 hours to 40 minutes. The simulations were carried out using MATLAB on the Euler computing cluster of ETH Zurich [9]. Numerical values: $\gamma_1 = 0.8, \gamma_2 = k_1 = \theta = \delta_0 = 1, \mu = \eta = 10, a = d = 100, \delta_0 = 1, \delta = 0.005, k = 0.05$.

4

7 Supplementary Figures

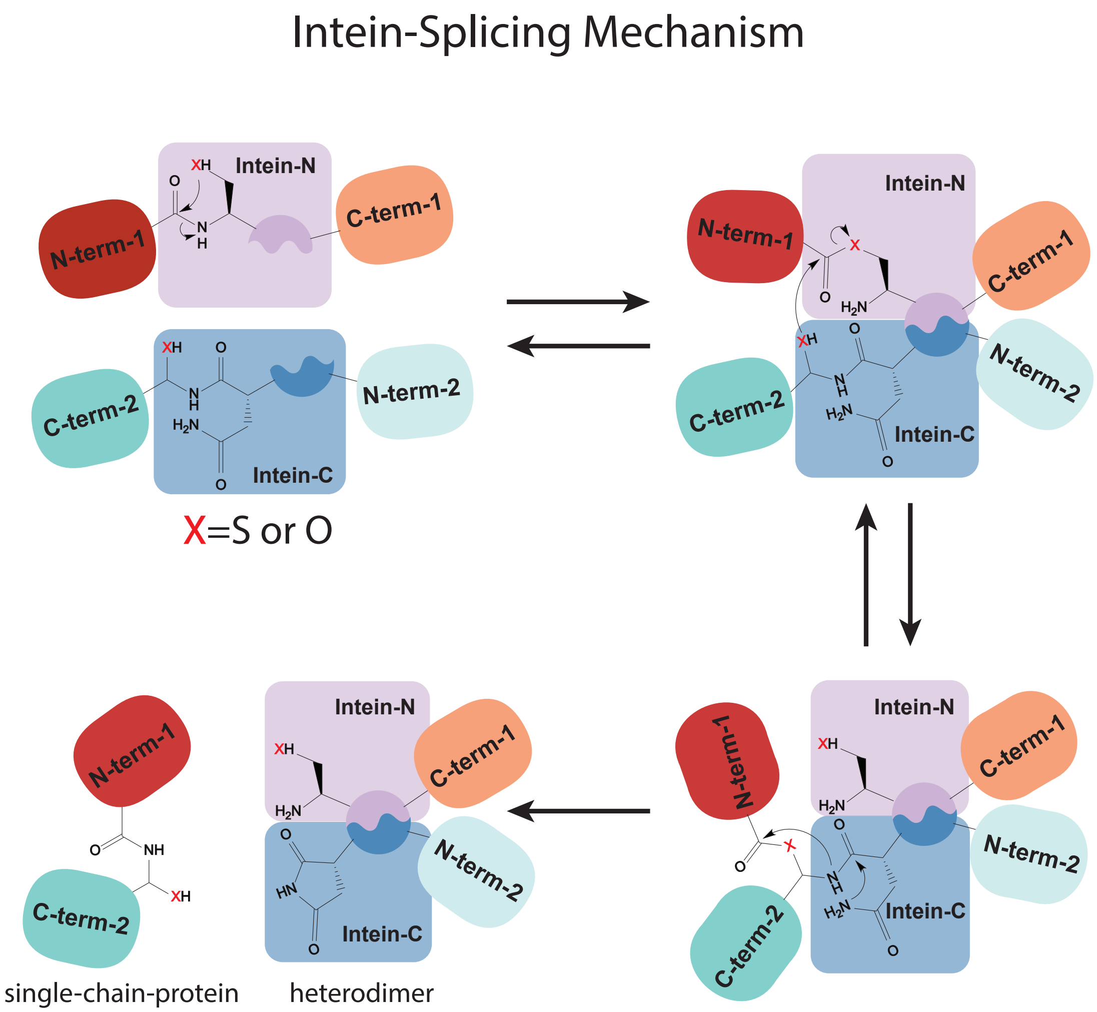

Supplementary Figure 22: Intein-Splicing Mechanism. An illustration of the canonical intein-splicing reaction is shown. The inteins are represented by the shaded squares. The chemical structure inside the square is only shown for sequences that directly participate in the biomolecular reaction. The split intein parts, which are responsible for dimerization are depicted as matching half circles. Both inteins are flanked with an N-terminal and a C-terminal domain. Note that redX is either a sulfur or oxygen atom originating either from a cysteine or serine. Only the last step in the intein splicing reaction is irreversible. The two initial proteins lead to the production of three separate products: a single-chain-protein and a strongly bound heterodimer. Note that after the reaction N-term-1 is covalently bound to C-term-2 instead of to C-term-1. Furthermore, the essential sequences in the inteins are altered. These biochemical changes make it impossible for the intein to react a second time.

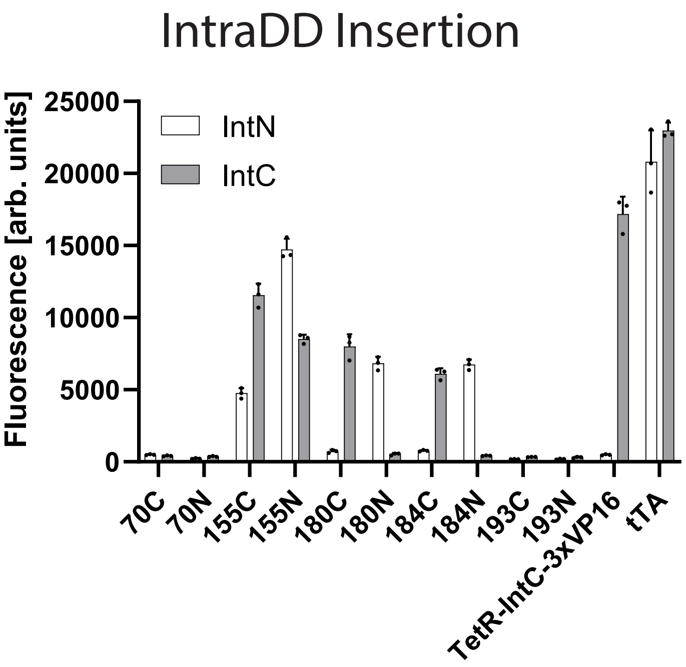

Supplementary Figure 23: Intein Insertion sites in the DD of TetR. The fluorescent reporter miRFP670 under the TRE promoter was used for testing the functionality of TetR. tTA and TetR-IntC-3xVP16 were used as references to which the intraDD constructs were compared. The number on the x-axis, for the intraDD constructs, indicates the amino acid position of TetR at which the split intein was introduced. Split sites were selected based on the crystal structure and the study by Zeng et al. [10]. The letter indicates whether $\mathrm{Int}^{\mathrm{N}}$ (N) or $\mathrm{Int}^{\mathrm{C}}$ (C) was inserted. Every TF was transfected together with either a constitutively expressed $\mathrm{Int}^{\mathrm{N}}$ (white bars) or $\mathrm{Int}^{\mathrm{C}}$ (grey bars). tTA is a strong activator in both measurements as it is incapable of interacting with either of the inteins. A decrease in the TetR-IntC-3xVP16 measurement was observed only with $\mathrm{Int}^{\mathrm{N}}$. This construct was used for the TetR circuit. Insertion sites 70 and 193 did not lead to functional tTAs and therefore violated Building Consideration 1. In contrast, insertion site 155 fulfilled Building Consideration 1, however, it violated Building Consideration 2 and/or 3 as the inhibition was less than two folds. 180 and 184 fulfilled all building considerations independent of whether $\mathrm{Int}^{\mathrm{N}}$ or $\mathrm{Int}^{\mathrm{C}}$ was introduced. The construct 184C was eventually used for the intraDD circuit. Transfection tables are given in Supplementary Tables 26 and 27. For all the data, the HEK293T cells were measured using flow cytometry 48 h after transfection, and the normalized data are shown as mean + SD for n = 3 technical replicates.

Building Consideration 1

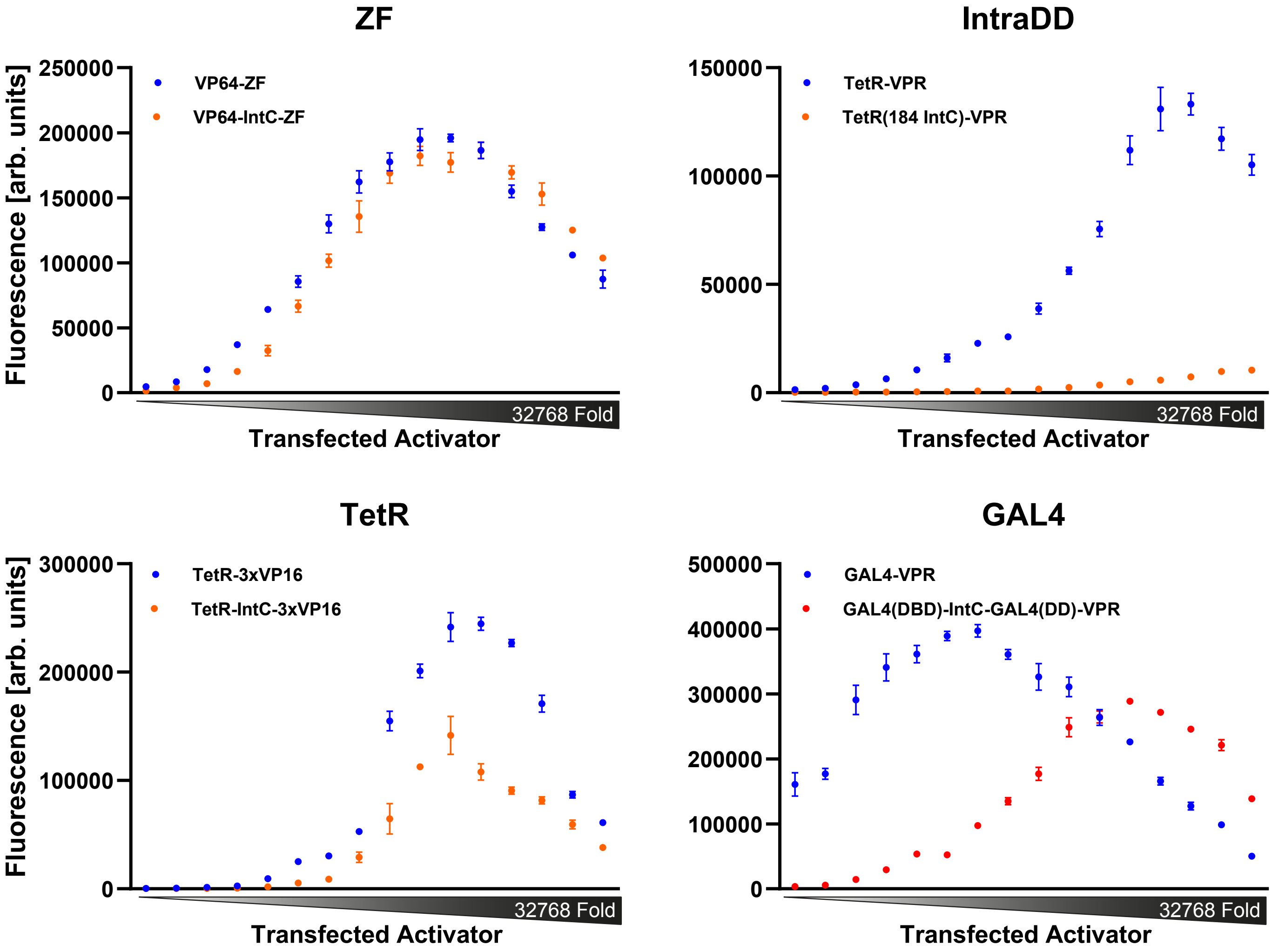

Supplementary Figure 24: Testing for Building Consideration 1. The activity of the four unmodified TF (blue) were quantified by observing the fluorescent reporter moxVenus. The experiment was repeated with all TF after the insertion of the split intein (orange). The x-axis illustrates the amount of transfected plasmid that encode the transcription factors. Transfection tables are given in Supplementary Tables 28 and 29. For all the data, the HEK293T cells were measured using flow cytometry 48 h after transfection, and the normalized data are shown as mean +/- SD for n = 3 technical replicates.

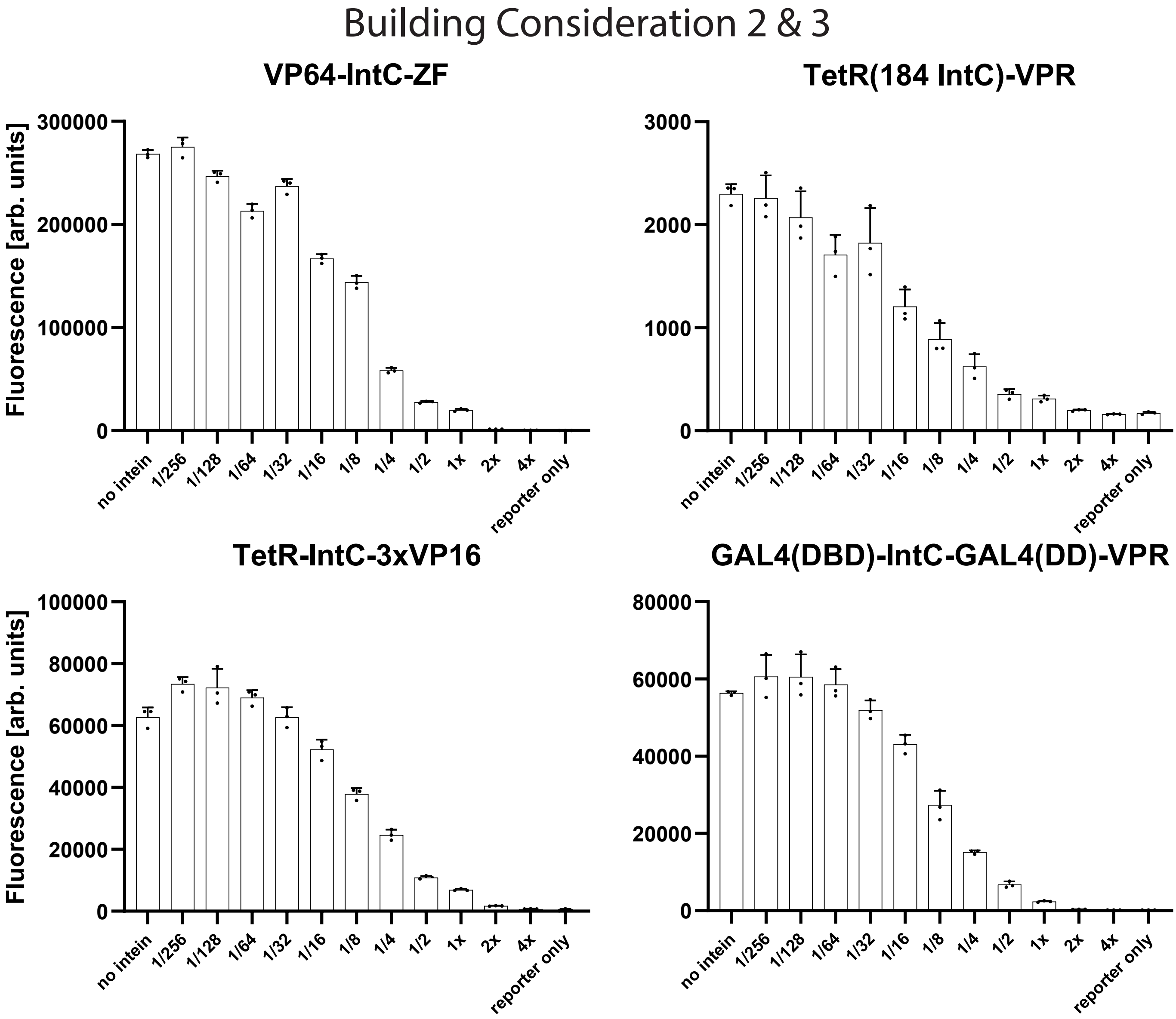

Supplementary Figure 25: Testing for Building Consideration 2 & 3. The y-axis denotes the fluorescent reporter levels of moxVenus, which is regulated by a TF carrying an Int^{C}. A separate plasmid encoding an Int^{N} was transfected together with the reporter plasmid and the TF plasmid. The x-axis illustrates the ratio between the TF plasmid and the Int^{N} plasmid. Two additional measurements were added for reference, one without the Int^{N} plasmid and one with only the reporter plasmid. The reference measurments indicate the maximum expression for this TF concentration and the minimal expression due to reporter leakiness. Complete inhibition towards leakiness levels were reached upon high Int^{N} levels for all TF designs. Transfection tables are given in Supplementary Tables 30 and 31. For all the data, the HEK293T cells were measured using flow cytometry 48 h after transfection, and the normalized data are shown as mean + SD for n = 3 technical replicates.

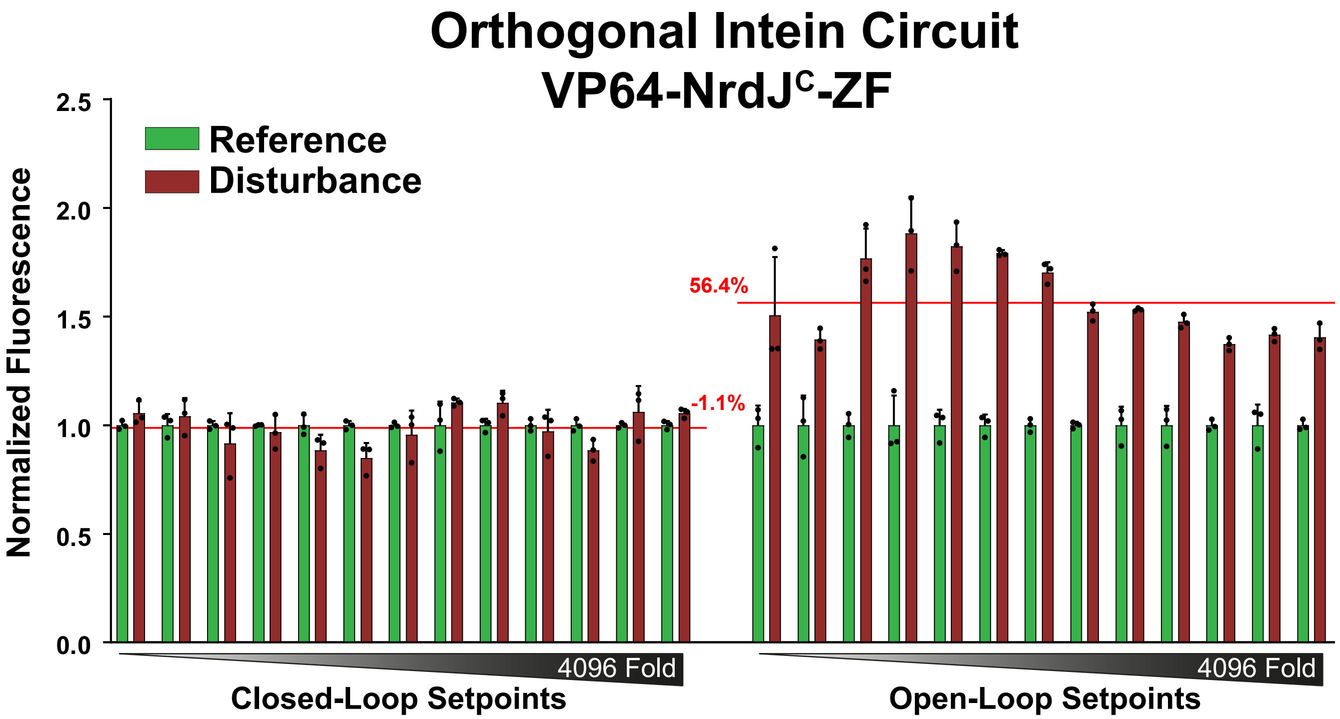

Supplementary Figure 26: Orthogonal Intein. The bar graph shows the normalized fluorescence of the proxy reporter with (disturbance, red) and without (reference, green) disturbance and for both the closed-loop (left) and open-loop (right) settings. In the closed-loop the output produced the matching Int^{N} of NrdJ-1 and in the open-loop the output produced the orthogonal Int^{N} of Gp41-1. The disturbed and reference triplicate measurements were normalized to the mean fluorescence of the reference data for each setpoint. The x-axis follows a $\log_2$-scale and shows the amount of plasmid 1 transfected within every well. The red horizontal lines give the normalized output averaged over all the setpoints, and the numbers above the lines indicate the averaged error of the disturbed output relative to the reference. The transfection table is given in Supplementary Table 32. For all the data, the HEK293T cells were measured using flow cytometry 48 h after transfection, and the normalized data are shown as mean + SD for n = 3 technical replicates.

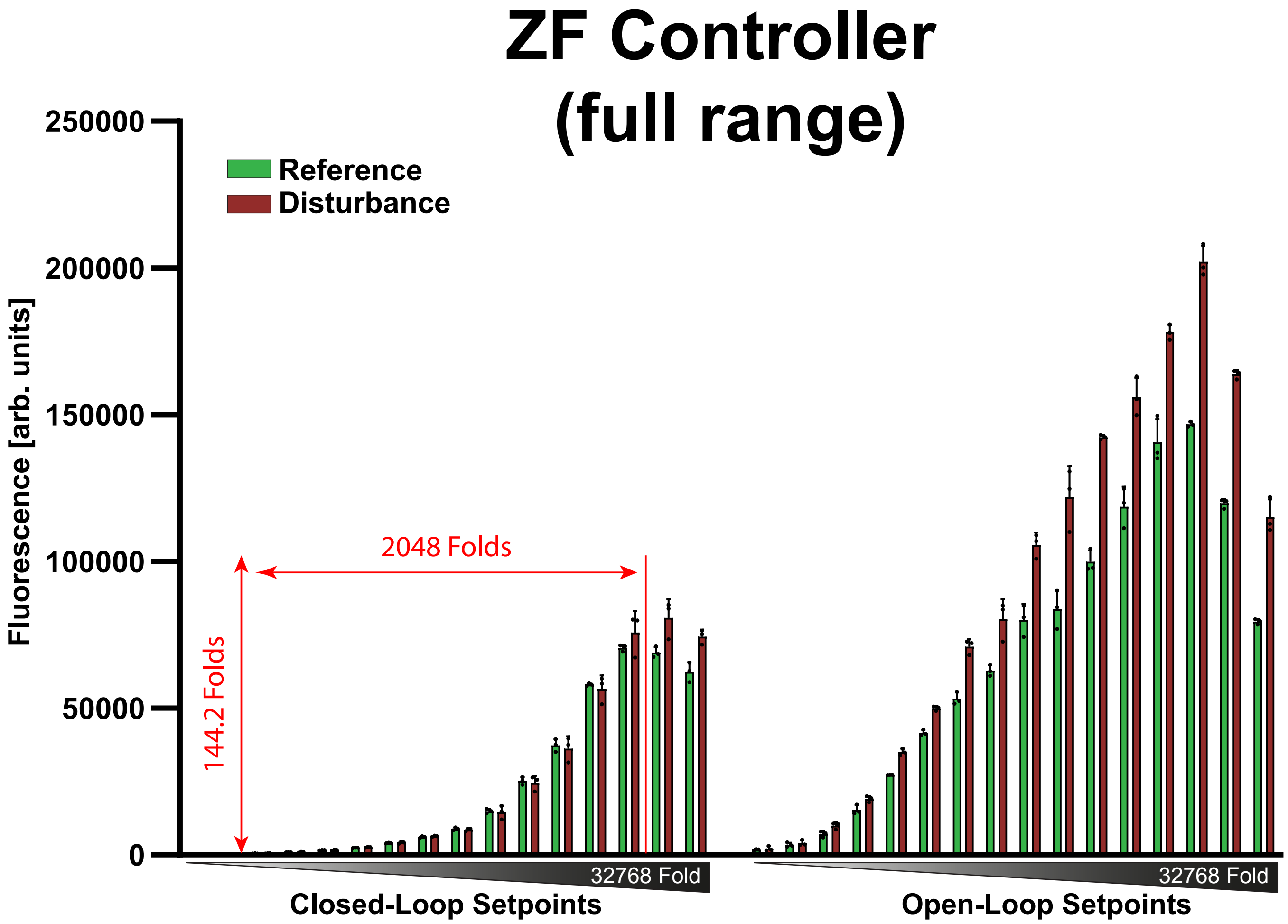

Supplementary Figure 27: Full Range of the ZF Controller. A representative measurement for the entire range experimentally tested for the ZF circuit. Setpoint that were too close to the background were not included in the dynamic range. High setpoints were excluded as well due to their lack of RPA. At high setpoints a drop in fluorescence can be observed, which is most likely due to a combination of saturation and burden. The dynamic range of the ZF circuit is shown in the red arrows in the closed loop. The horizontal value depicts the input dynamic range (fold change in transfected plasmid 1) and the vertical value depicts the output dynamic range (the fold change in the fluorescence). The transfection table is given in Supplementary Tables 21. For all the data, the HEK293T cells were measured using flow cytometry 48 h after transfection, and the normalized data are shown as mean + SD for n = 3 technical replicates.

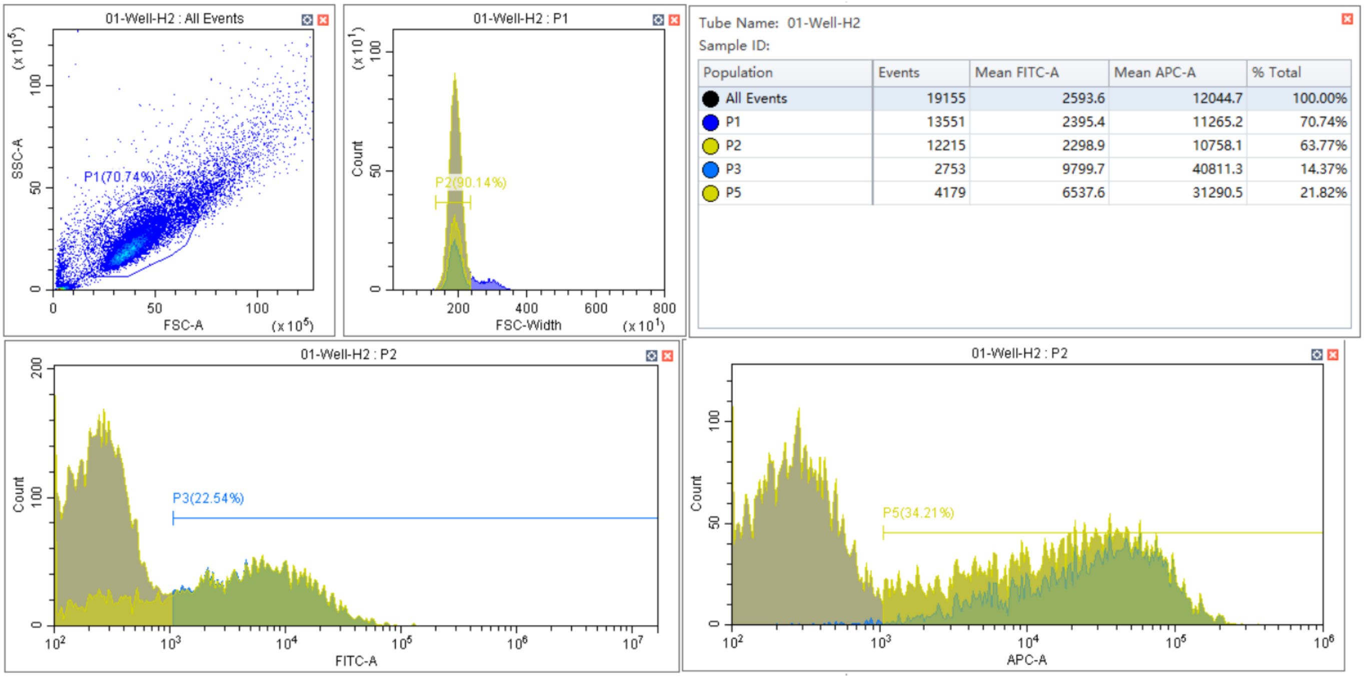

Population	Events	Mean FITC-A	Mean APC-A	% Total
All Events	19155	2593.6	12044.7	100.00%
P1	13551	2395.4	11265.2	70.74%
P2	12215	2298.9	10758.1	63.77%
P3	2753	9799.7	40811.3	14.37%
P5	4179	6537.6	31290.5	21.82%

Supplementary Figure 28: A representative example illustrating the gating strategy. The first gate P1 excludes debris and dead cells based on their size in the SSC-A and the FSC-A. The gate P2 is applied on the P1 subset and selects only single cells based on the FSC-Width. This population is used for the gating of positively transfected cells in the FITC-A channel and the APC-A channel, respectively.

8 Amino Acid Sequences

Controllers/Co-Controllers:
VP64-**IntC(GP41-1)**-ZF
MPKKKRKVGSGEFDALDDFDLDMLGSDALDDFDLDMLGSDALDDFDLDMLGSDALDDFDLDM
LGSGGGGSGGGGSGGGGSATM**MLKKILKIEELDERELIDIEVSGNHLFYANDILTHNS**NRGGG
GSGGGGSGGGGSGTARPGERPFQCRICMRNFSRQDRLDRHTRTHTGEKPFQCRICMRNFSQ
KEHLAGHLRTHTGEKPFQCRICMRNFSRRDNLNRHLKTHLRGS

VP64-**IntC(NrdJ-1)** -ZF

MPKKKRKVGSGEFDALDDFDLDMLGSDALDDFDLDMLGSDALDDFDLDMLGSDALDDFDLDM
LGSGGGGSGGGGSGGGGSATMG**SMEAKTYIGKLKSRKIVSNEDTYDIQTSTHNFFANDILVH
NSEI**GSNRGGGGSGGGGSGGGGSGTARPGERPFQCRICMRNFSRQDRLDRHTRTHTGEKPF
QCRICMRNFSQKEHLAGHLRTHTGEKPFQCRICMRNFSRRDNLNRHLKTHLRGS

GAL4(DBD)**_IntC(GP41-1)_**GAL4(DD)-**VPR(212)**
MKLLSSIEQACDICRLKKLKCSKEKPKCAKCLKNNWECRYSPKTKRSPLTRAHLTEVESRLERL
EGS**MLKKILKIEELDERELIDIEVSGNHLFYANDILTHNS**GSGSQLFLLIFPREDLDMILKMDSLQ
DIKALLTGLFVQDNVNKDAVTDRLASVETDMPLTLRQHRISATSSSEESSNKGQRQLTVSGSS**E
ASGSGRADALDDFDLDMLGSDALDDFDLDMLGSDALDDFDLDMLGSDALDDFDLDMLINSR
SSGSPKKKRKVGSQYLPDTDDRHRIEEKRKRTYETFKSIMKKSPFSGPTDPRPPPRRIAVPSR
SSASVPKPAPQPYPFTSSLSTINYDEFPTMVFPSGQISQASALAPAPPQVLPQAPAPAPAPAM
VSALAQAPAPVPVLAPGPPQAVAPPAPKPTQAGEGTLSEALLQLQFDDEDLGALLGNSTDP
AVFTDLASVDNSEFQQLLNQGIPVAPHTTEPMLMEYPEAITRLVTGAQRPPDPAPAPLGAPGL
PNGLLSGDEDFSSIADMDFSALLGSGSGSRDSREGMFLPKPEAGSAISDVFEGREVCQPKRI**

tetR**_IntC(GP41-1)**_VP64

MSRLDKSKVINSALELLNEVGIEGLTTRKLAQKLGVEQPTLYWHVKNKRALLDALAIEMLDRHHT
HFCPLEGESWQDFLRNKAKSFRCALLSHRDGAKVHLGTRPTEKQYETLENQLAFLCQQGFSL
ENALYALSAVGHFTLGCVLEDQEHQVAKEERETPTTDSMPPLLRQAIELFDHQGAEPAFLFGLE
LIICGLEKQLKCESGDSGGGGS**MLKKILKIEELDERELIDIEVSGNHLFYANDILTHNS**GSGSGR
ADALDDFDLDMLGSDALDDFDLDMLGSDALDDFDLDMLGSDALDDFDLDMLIN

tetR(1-183)**_IntC(GP41-1)**_tetR(184-212)**_VPR**
MSRLDKSKVINSALELLNEVGIEGLTTRKLAQKLGVEQPTLYWHVKNKRALLDALAIEMLDRHHT
HFCPLEGESWQDFLRNNAKSFRCALLSHRDGAKVHLGTRPTEKQYETLENQLAFLCQQGFSL
ENALYALSAVGHFTLGCVLEDQEHQVAKEERETPTTDSMPPLLRQAIELFDHQGAEPGS**MLKK
ILKIEELDERELIDIEVSGNHLFYANDILTHNS**GSGSAFLFGLELIICGLEKQLKCESGSPADALDD
FDLDMLPADALDDFDLDMLPADALDDFDLDMLPGQPGSS**EASGSGRADALDDFDLDMLGSD
ALDDFDLDMLGSDALDDFDLDMLGSDALDDFDLDMLINSRSSGSPKKKRKVGSQYLPDTDD
RHRIEEKRKRTYETFKSIMKKSPFSGPTDPRPPPRRIAVPSRSSASVPKPAPQPYPFTSSLSTI
NYDEFPTMVFPSGQISQASALAPAPPQVLPQAPAPAPAPAMVSALAQAPAPVPVLAPGPPQ
AVAPPAPKPTQAGEGTLSEALLQLQFDDEDLGALLGNSTDPAVFTDLASVDNSEFQQLLNQ
GIPVAPHTTEPMLMEYPEAITRLVTGAQRPPDPAPAPLGAPGLPNGLLSGDEDFSSIADMDFS
ALLGSGSGSRDSREGMFLPKPEAGSAISDVFEGREVCQPKRIRPFHPPGSPWANRPLPASLA
PTPTGPVHEPVGSLTPAPVPQPLDPAPAVTPEASHLLEDPDEETSQAVKALREMADTVIPQK**

Supplementary Figure 29: Sequences

Controllers/Co-Controllers:

TetR-**dead IntN**

MSRLDKSKVINSALELLNEVGIEGLTTRKLAQKLGVEQPTLYWHVKNKRALLDALAIEMLDRHHT
HFCPLEGESWQDFLRNNAKSFRCALLSHRDGAKVHLGTRPTEKQYETLENQLAFLCQQGFSL
ENALYALSAVGHFTLGCVLEDQEHQVAKEERETPTTDSMPPLLRQAIELFDHQGAEPAFLFGLE
LIICGLEKQLKCESGSGGGGSGGGGTGGGGTG**TGYNEVLNVFPKSKKKSYKITLEDGKEIICS
EEHLFPTQTGEMNISGGLKEGMCLYVKEGST**

IntC-**VPR**

MMLKKILKIEELDERELIDIEVSGNHLFYANDILTHNSGSGSS**EASGSGRADALDDFDLDMLGS
DALDDFDLDMLGSDALDDFDLDMLGSDALDDFDLDMLINSRSSGSPKKKRKVGSQYLPDTD
DRHRIEEKRKRTYETFKSIMKKSPFSGPTDPRPPPRRIAVPSRSSASVPKPAPQPYPFTSSLST
INYDEFPTMVFPSGQISQASALAPAPPQVLPQAPAPAPAPAMVSALAQAPAPVPVLAPGPPQ
AVAPPAPKPTQAGEGTLSEALLQLQFDDEDLGALLGNSTDPAVFTDLASVDNSEFQQLLNQ
GIPVAPHTTEPMLMEYPEAITRLVTGAQRPPDPAPAPLGAPGLPNGLLSGDEDFSSIADMDFS
ALLGSGSGSRDSREGMFLPKPEAGSAISDVFEGREVCQPKRIRPFHPPGSPWANRPLPASLA
PTPTGPVHEPVGSLTPAPVPQPLDPAPAVTPEASHLLEDPDEETSQAVKALREMADTVIPQK
EEAAICGQMDLSHPPPRGHLDELTTTLESMTEDLNLDSPLTPELNEILDTFLNDECLLHAMHIS**

Supplementary Figure 30: Sequences

Anticontrollers/Open-loop controls + Proxy-Reporter:

IntN(Gp41-1)_**p2A-t2A**_moxVenus

MGGGGSGGGGTGGGGTGTRSGYCLDLKTQVQTPQGMKEISNIQVGDLVLSNTGYNEVLNVF
PKSKKKSYKITLEDGKEIICSEEHLFPTQTGEMNISGGLKEGMCLYVKEG**SGSGATNFSLLKQA
GDVEENPGPGSGEGRGSLLTCGDVEENPGPIDGGSG**GSATMVSKGEELFTGVVPILVELDGD
VNGHKFSVSGEGEGDATYGKLTLKLICTTGKLPVPWPTLVTTLGYGLQCFARYPDHMKQHDFF
KSAMPEGYVQERTIFFKDDGNYKTRAEVKFDGDTLVNRIELKGIDFKEDGNILGHKLEYNYNSH
NVYITADKQKNGIKANFKIRHNIEDGGVQLADHYQQNTPIGDGPVLLPDNHYLSYQSKLSKDPN
EKRDHMVLLEFVTAAGITLGMDELYKGSGSGS

IntC(Gp41-1)_**p2A-t2A**_moxVenus

MMLKKILKIEELDERELIDIEVSGNHLFYANDILTHNSGSG**SGSGATNFSLLKQAGDVEENPGPG
SGEGRGSLLTCGDVEENPGPIDGGSG**GSATMVSKGEELFTGVVPILVELDGDVNGHKFSVSG
EGEGDATYGKLTLKLICTTGKLPVPWPTLVTTLGYGLQCFARYPDHMKQHDFFKSAMPEGYVQ
ERTIFFKDDGNYKTRAEVKFDGDTLVNRIELKGIDFKEDGNILGHKLEYNYNSHNVYITADKQKN
GIKANFKIRHNIEDGGVQLADHYQQNTPIGDGPVLLPDNHYLSYQSKLSKDPNEKRDHMVLLEF
VTAAGITLGMDELYKGSGSGS

IntN(NrdJ-1)**_p2A-t2A**_moxVenus

MNPCCLVGSSEIITRNYGKTTIKEVVEIFDNDKNIQVLAFNTHTDNIEWAPIKAAQLTRPNAELVE
LEIDTLHGVKTIRCTPDHPVYTKNRGYVRADELTDDDELVVAIG**SGSGATNFSLLKQAGDVEEN
PGPGSGEGRGSLLTCGDVEENPGPIDGGSG**GSATMVSKGEELFTGVVPILVELDGDVNGHKF
SVSGEGEGDATYGKLTLKLICTTGKLPVPWPTLVTTLGYGLQCFARYPDHMKQHDFFKSAMPE
GYVQERTIFFKDDGNYKTRAEVKFDGDTLVNRIELKGIDFKEDGNILGHKLEYNYNSHNVYITAD
KQKNGIKANFKIRHNIEDGGVQLADHYQQNTPIGDGPVLLPDNHYLSYQSKLSKDPNEKRDHM
VLLEFVTAAGITLGMDELYKGSGSGS

IntC(NrdJ-1)_p**2A-t2A**_moxVenus

MEAKTYIGKLKSRKIVSNEDTYDIQTSTHNFFANDILVHNSEIG**SGSGATNFSLLKQAGDVEENP
GPGSGEGRGSLLTCGDVEENPGPIDGGSG**GSATMVSKGEELFTGVVPILVELDGDVNGHKFS
VSGEGEGDATYGKLTLKLICTTGKLPVPWPTLVTTLGYGLQCFARYPDHMKQHDFFKSAMPEG
YVQERTIFFKDDGNYKTRAEVKFDGDTLVNRIELKGIDFKEDGNILGHKLEYNYNSHNVYITADK
QKNGIKANFKIRHNIEDGGVQLADHYQQNTPIGDGPVLLPDNHYLSYQSKLSKDPNEKRDHMV
LLEFVTAAGITLGMDELYKGSGSGS

Supplementary Figure 31: Sequences

9 Supplementary Tables

ZF-Controller									Deviation from Reference in %		
Closed Loop	6.1	1.0	2.2	7.7	5.3	4.8	-3.4	-2.7	-2.9	-3.1	-2.6	7.4
Open Loop	39.8	24.1	28.4	20.1	33.3	28.2	31.9	45.4	42.5	31.5	26.7	37.7

IntraDD-Controller					Deviation from Reference in %		
IntraDD-circuit	7.3	1.5	10.3	10.9	-8.3	10.2	11.1	3.0
IntraDD-circuit	40.9	54.2	53.4	31.3	18.8	37.8	38.3	72.5

GAL4-Controller					Deviation from Reference in %		
Closed Loop	-1.9	1.3	4.6	4.2	-7.9	-4.0	4.4
Open Loop	29.3	39.3	32.0	38.3	36.2	18.3	25.8

TetR-Controller						Deviation from Reference in %		
Closed Loop	-4.3	-13.0	1.7	-12.6	-7.3	-3.3	-1.9	7.9
Open Loop	49.4	31.4	38.9	29.5	35.2	31.3	18.6	17.6

inactive-Controller								Deviation from Reference in %		
Closed Loop	6.2	5.9	-1.3	4.1	2.2	6.6	8.1	0.7	1.9	5.1
Open Loop	37.1	37.3	39.0	42.1	40.6	34.8	47.3	38.2	33.5	59.5

NrdJ-Controller											Deviation from Reference in %		
Closed Loop	5.5	4.2	-8.3	-3.2	-11.6	-15.1	-4.4	10.6	10.4	-2.8	-11.5	6.2	5.5
Open Loop	50.6	39.5	76.9	88.4	82.5	79.2	70.3	52.1	53.3	47.6	37.3	41.7	40.4

Supplementary Table 19: The percentage deviation from the setpoint for all the tested genetic circuits.

ID	Description	kDa	Source
SA152	EF1a_mCitrine-IntC(Gp41-1)_lateSV40polyA	2806	This Study
SA153	EF1a_mCitrine-IntN(Gp41-1)_lateSV40polyA	2936	This Study
SA172	EF1a_VP64-IntC(Gp41-1)-ZF_lateSV40polyA	2708	This Study
SA193	EF1a_TetR-IntC(Gp41-1)-VP64_lateSV40polyA	2855	This Study
SA231	EF1a_tTA-IntN(Gp41-1)(insert at 70aa)_lateSV40polyA	2960	This Study
SA232	EF1a_tTA-IntC(Gp41-1)(insert at 180aa)_lateSV40polyA	2830	This Study
SA233	EF1a_tTA-IntN(Gp41-1)(insert at 180aa)_lateSV40polyA	2960	This Study
SA234	EF1a_tTA-IntC(Gp41-1)(insert at 184aa)_lateSV40polyA	2830	This Study
SA235	EF1a_tTA-IntC(Gp41-1)(insert at 193aa)_lateSV40polyA	2830	This Study
SA236	EF1a_tTA-IntN(Gp41-1)(insert at 193aa)_lateSV40polyA	2960	This Study
SA237	EF1a_tTA-IntC(Gp41-1)(insert at 70aa)_lateSV40polyA	2830	This Study
SA238	EF1a_tTA-IntN(Gp41-1)(insert at 184aa)_lateSV40polyA	2960	This Study
SA239	EF1a_tTA-IntN(Gp41-1)(insert at 155aa)_lateSV40polyA	2960	This Study
SA247	EF1a_tTA-IntC(Gp41-1)(insert at 155aa)_lateSV40polyA	2830	This Study
SA263	12xpZF_IntN(Gp41-1)(SA255)-P2A-T2A-mVenus_lateSV40polyA	2459	This Study
SA264	5xpUAS_IntC(Gp41-1)-P2A-T2A-mVenus_lateSV40polyA	2354	This Study
SA265	5xpUAS_IntN(Gp41-1)(SA255)-P2A-T2A-mVenus_lateSV40polyA	2483	This Study
SA266	7xpTRE_IntC(Gp41-1)-P2A-T2A-mVenus_lateSV40polyA	2399	This Study
SA267	7xpTRE_IntN(Gp41-1)-P2A-T2A-mVenus_lateSV40polyA	2529	This Study
SA272	12xpZF_IntC(Gp41-1)-P2A-T2A-mVenus_lateSV40polyA	2329	This Study
SA286	EF1a_Gal4(DBD)_IntC(Gp41-1)_Gal4(DD)-VPR_lateSV40polyA	3629	This Study
SA324	EF1a_TetR-IntC(Gp41-1)(insert at 184aa)-VPR_lateSV40polyA	3820	This Study
SA478	7xpTRE_moxVenus_SV40t	2281	This Study
SA479	12xpZF_moxVenus_SV40t	2210	This Study
SA480	5xpUAS_moxVenus_SV40t	2235	This Study
SA489	EF1a_VP64-IntC(NrdJ-1S2)-ZF_lateSV40polyA	2726	This Study
SA497	12xpZF_IntN(NrdJ-1S2)-P2A-T2A-mVenus_lateSV40polyA	2457	This Study
SA506	7xpTRE_IntC(NrdJ-1S2)-P2A-T2A-mVenus_lateSV40polyA	2405	This Study
MTK833	7xpTRE_miRFP670_lateSV40polyA	2358	This Study
MTK837	EF1a_tTA_lateSV40polyA	2750	This Study
MTK1138	EF1a_IntN(Gp41-1)_lateSV40polyA	2491	This Study
GLM171	empty backbone	1954	[11]
TF138	EF1a_miRFP670_lateSV40polyA	3286	[12]
CH335	EF1a_TetR-IntN(Δ1-29)_lateSV40polyA	3208	This Study
CH340	EF1a_IntC-VPR_lateSV40polyA	3414	This Study

Supplementary Table 20: Plasmid List.

Closed Loop

Reference														Units:	ng
Activator	SA172	0.0025	0.0050	0.0100	0.0199	0.0398	0.0797	0.1594	0.3188	0.6375	1.2750	2.5500	5.1000	10.2000	20.3000	40.6000	81.2310
Reporter	SA263	4.4222	4.4222	4.4222	4.4222	4.4222	4.4222	4.4222	4.4222	4.4222	4.4222	4.4222	4.4222	4.4222	4.4222	4.4222	4.4222
Const. Color	TF138	5.0000	5.0000	5.0000	5.0000	5.0000	5.0000	5.0000	5.0000	5.0000	5.0000	5.0000	5.0000	5.0000	5.0000	5.0000	5.0000
Stuffer	GLM171	90.5753	90.5728	90.5678	90.5578	90.5379	90.4981	90.4184	90.2590	89.9403	89.3028	88.0278	85.4778	80.3778	70.2778	49.9778	9.3468

Disturbance																Units:	ng
Activator	SA172	0.0025	0.0050	0.0100	0.0199	0.0398	0.0797	0.1594	0.3188	0.6375	1.2750	2.5500	5.1000	10.2000	20.3000	40.6000	81.2310
Reporter	SA263	7.3761	7.3761	7.3761	7.3761	7.3761	7.3761	7.3761	7.3761	7.3761	7.3761	7.3761	7.3761	7.3761	7.3761	7.3761	7.3761
Const. Color	TF138	5.0000	5.0000	5.0000	5.0000	5.0000	5.0000	5.0000	5.0000	5.0000	5.0000	5.0000	5.0000	5.0000	5.0000	5.0000	5.0000
Stuffer	GLM171	87.6214	87.6189	87.6139	87.6040	87.5841	87.5442	87.4645	87.3052	86.9864	86.3489	85.0739	82.5239	77.4239	67.3239	47.0239	6.3929

Open Loop

Reference																Units:	ng
Activator	SA172	0.0025	0.0050	0.0100	0.0199	0.0398	0.0797	0.1594	0.3188	0.6375	1.2750	2.5500	5.1000	10.2000	20.3000	40.6000	81.2310
Reporter	SA272	4.1920	4.1920	4.1920	4.1920	4.1920	4.1920	4.1920	4.1920	4.1920	4.1920	4.1920	4.1920	4.1920	4.1920	4.1920	4.1920
Const. Color	TF138	5.0000	5.0000	5.0000	5.0000	5.0000	5.0000	5.0000	5.0000	5.0000	5.0000	5.0000	5.0000	5.0000	5.0000	5.0000	5.0000
Stuffer	GLM171	90.8055	90.8030	90.7980	90.7881	90.7681	90.7283	90.6486	90.4892	90.1705	89.5330	88.2580	85.7080	80.6080	70.5080	50.2080	9.5770

Disturbance																Units:	ng
Activator	SA172	0.0025	0.0050	0.0100	0.0199	0.0398	0.0797	0.1594	0.3188	0.6375	1.2750	2.5500	5.1000	10.2000	20.3000	40.6000	81.2310
Reporter	SA272	6.9444	6.9444	6.9444	6.9444	6.9444	6.9444	6.9444	6.9444	6.9444	6.9444	6.9444	6.9444	6.9444	6.9444	6.9444	6.9444
Const. Color	TF138	5.0000	5.0000	5.0000	5.0000	5.0000	5.0000	5.0000	5.0000	5.0000	5.0000	5.0000	5.0000	5.0000	5.0000	5.0000	5.0000
Stuffer	GLM171	88.0531	88.0506	88.0456	88.0356	88.0157	87.9759	87.8962	87.7368	87.4181	86.7806	85.5056	82.9556	77.8556	67.7556	47.4556	6.8246

Supplementary Table 21: Transfection Table for the ZF Controller. This transfection table shows all the tested setpoints for the ZF controller presented in Fig. 4. The normalized data plots for the ZF controller depicted in Fig. 4 show only the values in black that correspond to the setpoints exhibiting RPA when operating in closed loop. The greyed out values denote the setpoints that do not exhibit RPA when operating in closed loop.

Closed Loop

Reference																Units:	ng
Activator	SA193	0.0026	0.0052	0.0105	0.0209	0.0418	0.0836	0.1673	0.3345	0.6691	1.3381	2.6763	5.3526	10.7051	21.4103	42.8205	85.6410
Reporter	SA267	5.6905	5.6905	5.6905	5.6905	5.6905	5.6905	5.6905	5.6905	5.6905	5.6905	5.6905	5.6905	5.6905	5.6905	5.6905	5.6905
Const. Color	TF138	5.0000	5.0000	5.0000	5.0000	5.0000	5.0000	5.0000	5.0000	5.0000	5.0000	5.0000	5.0000	5.0000	5.0000	5.0000	5.0000
Stuffer	GLM171	89.3069	89.3043	89.2991	89.2886	89.2677	89.2259	89.1423	88.9750	88.6405	87.9714	86.6332	83.9570	78.6044	67.8993	46.4890	3.6685

Disturbance																Units:	ng
Activator	SA193	0.0026	0.0052	0.0105	0.0209	0.0418	0.0836	0.1673	0.3345	0.6691	1.3381	2.6763	5.3526	10.7051	21.4103	42.8205	85.6410
Reporter	SA267	7.5873	7.5873	7.5873	7.5873	7.5873	7.5873	7.5873	7.5873	7.5873	7.5873	7.5873	7.5873	7.5873	7.5873	7.5873	7.5873
Const. Color	TF138	5.0000	5.0000	5.0000	5.0000	5.0000	5.0000	5.0000	5.0000	5.0000	5.0000	5.0000	5.0000	5.0000	5.0000	5.0000	5.0000
Stuffer	GLM171	87.4101	87.4075	87.4022	87.3918	87.3709	87.3291	87.2454	87.0782	86.7436	86.0746	84.7364	82.0601	76.7076	66.0025	44.5922	1.7717

Open Loop

Reference																Units:	ng
Activator	SA193	0.0026	0.0052	0.0105	0.0209	0.0418	0.0836	0.1673	0.3345	0.6691	1.3381	2.6763	5.3526	10.7051	21.4103	42.8205	85.6410
Reporter	SA266	5.6905	5.6905	5.6905	5.6905	5.6905	5.6905	5.6905	5.6905	5.6905	5.6905	5.6905	5.6905	5.6905	5.6905	5.6905	5.3984
Const. Color	TF138	5.0000	5.0000	5.0000	5.0000	5.0000	5.0000	5.0000	5.0000	5.0000	5.0000	5.0000	5.0000	5.0000	5.0000	5.0000	5.0000
Stuffer	GLM171	89.3069	89.3043	89.2991	89.2886	89.2677	89.2259	89.1423	88.9750	88.6405	87.9714	86.6332	83.9570	78.6044	67.8993	46.4890	3.9606

Disturbance																Units:	ng
Activator	SA193	0.0026	0.0052	0.0105	0.0209	0.0418	0.0836	0.1673	0.3345	0.6691	1.3381	2.6763	5.3526	10.7051	21.4103	42.8205	85.6410
Reporter	SA266	7.1979	7.1979	7.1979	7.1979	7.1979	7.1979	7.1979	7.1979	7.1979	7.1979	7.1979	7.1979	7.1979	7.1979	7.1979	7.1979
Const. Color	TF138	5.0000	5.0000	5.0000	5.0000	5.0000	5.0000	5.0000	5.0000	5.0000	5.0000	5.0000	5.0000	5.0000	5.0000	5.0000	5.0000
Stuffer	GLM171	87.7995	87.7969	87.7916	87.7812	87.7603	87.7185	87.6348	87.4676	87.1330	86.4640	85.1258	82.4495	77.0970	66.3919	44.9816	2.1611

Supplementary Table 22: Transfection Table for the intraDD Controller. This transfection table shows all the tested setpoints for the intraDD controller presented in Fig. 4. The normalized data plots for the intraDD controller depicted in Fig. 4 show only the values in black that correspond to the setpoints exhibiting RPA when operating in closed loop. The greyed out values denote the setpoints that do not exhibit RPA when operating in closed loop. Note that greyed out values of the open loop circuit are also used in Fig. 4 for the matching setpoints by fluorescence levels.

Closed Loop

Reference																Units:	ng
Activator	SA324	0.0023	0.0047	0.0093	0.0187	0.0373	0.0746	0.1492	0.2984	0.5969	1.1938	2.3875	4.7750	9.5500	19.1000	38.2000	76.4000
Reporter	SA267	5.6905	5.6905	5.6905	5.6905	5.6905	5.6905	5.6905	5.6905	5.6905	5.6905	5.6905	5.6905	5.6905	5.6905	5.6905	5.6905
Const. Color	TF138	5.0000	5.0000	5.0000	5.0000	5.0000	5.0000	5.0000	5.0000	5.0000	5.0000	5.0000	5.0000	5.0000	5.0000	5.0000	5.0000
Stuffer	GLM171	89.3072	89.3049	89.3002	89.2909	89.2722	89.2349	89.1603	89.0111	88.7127	88.1158	86.9220	84.5345	79.7595	70.2095	51.1095	12.9095

Disturbance																Units:	ng
Activator	SA324	0.0023	0.0047	0.0093	0.0187	0.0373	0.0746	0.1492	0.2984	0.5969	1.1938	2.3875	4.7750	9.5500	19.1000	38.2000	76.4000
Reporter	SA267	7.5873	7.5873	7.5873	7.5873	7.5873	7.5873	7.5873	7.5873	7.5873	7.5873	7.5873	7.5873	7.5873	7.5873	7.5873	7.5873
Const. Color	TF138	5.0000	5.0000	5.0000	5.0000	5.0000	5.0000	5.0000	5.0000	5.0000	5.0000	5.0000	5.0000	5.0000	5.0000	5.0000	5.0000
Stuffer	GLM171	87.4104	87.4080	87.4034	87.3940	87.3754	87.3381	87.2635	87.1143	86.8158	86.2190	85.0252	82.6377	77.8627	68.3127	49.2127	11.0127

Open Loop

Reference																Units:	ng
Activator	SA324	0.0023	0.0047	0.0093	0.0187	0.0373	0.0746	0.1492	0.2984	0.5969	1.1938	2.3875	4.7750	9.5500	19.1000	38.2000	76.4000
Reporter	SA266	5.6905	5.6905	5.6905	5.6905	5.6905	5.6905	5.6905	5.6905	5.6905	5.6905	5.6905	5.6905	5.6905	5.6905	5.6905	5.3984
Const. Color	TF138	5.0000	5.0000	5.0000	5.0000	5.0000	5.0000	5.0000	5.0000	5.0000	5.0000	5.0000	5.0000	5.0000	5.0000	5.0000	5.0000
Stuffer	GLM171	89.3072	89.3049	89.3002	89.2909	89.2722	89.2349	89.1603	89.0111	88.7127	88.1158	86.9220	84.5345	79.7595	70.2095	51.1095	13.2016

Disturbance																Units:	ng
Activator	SA324	0.0023	0.0047	0.0093	0.0187	0.0373	0.0746	0.1492	0.2984	0.5969	1.1938	2.3875	4.7750	9.5500	19.1000	38.2000	76.4000
Reporter	SA266	7.1979	7.1979	7.1979	7.1979	7.1979	7.1979	7.1979	7.1979	7.1979	7.1979	7.1979	7.1979	7.1979	7.1979	7.1979	7.1979
Const. Color	TF138	5.0000	5.0000	5.0000	5.0000	5.0000	5.0000	5.0000	5.0000	5.0000	5.0000	5.0000	5.0000	5.0000	5.0000	5.0000	5.0000
Stuffer	GLM171	87.7998	87.7974	87.7928	87.7834	87.7648	87.7275	87.6529	87.5037	87.2052	86.6084	85.4146	83.0271	78.2521	68.7021	49.6021	11.4021

Supplementary Table 23: This transfection table shows all the tested setpoints for the TetR controller presented in Fig. 4. The normalized data plots for the TetR controller depicted in Fig. 4 show only the values in black that correspond to the setpoints exhibiting RPA when operating in closed loop. The greyed out values denote the setpoints that do not exhibit RPA when operating in closed loop.

Closed Loop

Reference																Units:	ng
Activator	SA286	0.0025	0.0050	0.0100	0.0199	0.0399	0.0797	0.1595	0.3190	0.6379	1.2759	2.5517	5.1034	10.2068	20.4137	40.8274	81.6548
Reporter	SA265	5.2151	5.2151	5.2151	5.2151	5.2151	5.2151	5.2151	5.2151	5.2151	5.2151	5.2151	5.2151	5.2151	5.2151	5.2151	5.2151
Const. Color	TF138	5.0000	5.0000	5.0000	5.0000	5.0000	5.0000	5.0000	5.0000	5.0000	5.0000	5.0000	5.0000	5.0000	5.0000	5.0000	5.0000
Stuffer	GLM171	89.7824	89.7799	89.7749	89.7649	89.7450	89.7051	89.6254	89.4659	89.1469	88.5090	87.2331	84.6814	79.5780	69.3712	48.9575	8.1301

Disturbance																Units:	ng
Activator	SA286	0.0025	0.0050	0.0100	0.0199	0.0399	0.0797	0.1595	0.3190	0.6379	1.2759	2.5517	5.1034	10.2068	20.4137	40.8274	81.6548
Reporter	SA265	7.4502	7.4502	7.4502	7.4502	7.4502	7.4502	7.4502	7.4502	7.4502	7.4502	7.4502	7.4502	7.4502	7.4502	7.4502	7.4502
Const. Color	TF138	5.0000	5.0000	5.0000	5.0000	5.0000	5.0000	5.0000	5.0000	5.0000	5.0000	5.0000	5.0000	5.0000	5.0000	5.0000	5.0000
Stuffer	GLM171	87.5473	87.5448	87.5398	87.5299	87.5099	87.4701	87.3903	87.2308	86.9119	86.2739	84.9981	82.4464	77.3430	67.1361	46.7224	5.8951

Open Loop

Reference																Units:	ng
Activator	SA286	0.0025	0.0050	0.0100	0.0199	0.0399	0.0797	0.1595	0.3190	0.6379	1.2759	2.5517	5.1034	10.2068	20.4137	40.8274	81.6548
Reporter	SA264	5.2151	5.2151	5.2151	5.2151	5.2151	5.2151	5.2151	5.2151	5.2151	5.2151	5.2151	5.2151	5.2151	5.2151	5.2151	4.9426
Const. Color	TF138	5.0000	5.0000	5.0000	5.0000	5.0000	5.0000	5.0000	5.0000	5.0000	5.0000	5.0000	5.0000	5.0000	5.0000	5.0000	5.0000
Stuffer	GLM171	89.7824	89.7799	89.7749	89.7649	89.7450	89.7051	89.6254	89.4659	89.1469	88.5090	87.2331	84.6814	79.5780	69.3712	48.9575	8.4027

Disturbance																Units:	ng
Activator	SA286	0.0025	0.0050	0.0100	0.0199	0.0399	0.0797	0.1595	0.3190	0.6379	1.2759	2.5517	5.1034	10.2068	20.4137	40.8274	81.6548
Reporter	SA264	7.0608	7.0608	7.0608	7.0608	7.0608	7.0608	7.0608	7.0608	7.0608	7.0608	7.0608	7.0608	7.0608	7.0608	7.0608	7.0608
Const. Color	TF138	5.0000	5.0000	5.0000	5.0000	5.0000	5.0000	5.0000	5.0000	5.0000	5.0000	5.0000	5.0000	5.0000	5.0000	5.0000	5.0000
Stuffer	GLM171	87.9367	87.9342	87.9292	87.9193	87.8993	87.8595	87.7797	87.6202	87.3013	86.6633	85.3875	82.8358	77.7324	67.5255	47.1118	6.2844

Supplementary Table 24: Transfection Table for the Gal4 Controller. This transfection table shows all the tested setpoints for the Gal4 controller presented in Fig. 4. The normalized data plots for the Gal4 controller depicted in Fig. 4 show only the values in black that correspond to the setpoints exhibiting RPA when operating in closed loop. The greyed out values denote the setpoints that do not exhibit RPA when operating in closed loop. Note that greyed out values of the open loop circuit are also used in Fig. 4 for the matching setpoints by fluorescence levels.

Closed Loop

Reference																Units:	ng
DBD	CH335	32.0800	32.0800	32.0800	32.0800	32.0800	32.0800	32.0800	32.0800	32.0800	32.0800	32.0800	32.0800	32.0800	32.0800	32.0800	32.0800
AD	CH340	0.0008	0.0017	0.0033	0.0067	0.0133	0.0267	0.0533	0.1067	0.2134	0.4268	0.8535	1.7070	3.4140	6.8280	13.6560	27.3120
Reporter	SA267	5.2000	5.2000	5.2000	5.2000	5.2000	5.2000	5.2000	5.2000	5.2000	5.2000	5.2000	5.2000	5.2000	5.2000	5.2000	5.2000
Const. Color	TF138	5.0000	5.0000	5.0000	5.0000	5.0000	5.0000	5.0000	5.0000	5.0000	5.0000	5.0000	5.0000	5.0000	5.0000	5.0000	5.0000
Stuffer	GLM171	57.7192	57.7183	57.7167	57.7133	57.7067	57.6933	57.6667	57.6133	57.5066	57.2933	56.8665	56.0130	54.3060	50.8920	44.0640	30.4080

Disturbance																Units:	ng
DBD	CH335	32.0800	32.0800	32.0800	32.0800	32.0800	32.0800	32.0800	32.0800	32.0800	32.0800	32.0800	32.0800	32.0800	32.0800	32.0800	32.0800
AD	CH340	0.0008	0.0017	0.0033	0.0067	0.0133	0.0267	0.0533	0.1067	0.2134	0.4268	0.8535	1.7070	3.4140	6.8280	13.6560	27.3120
Reporter	SA267	7.5870	7.5870	7.5870	7.5870	7.5870	7.5870	7.5870	7.5870	7.5870	7.5870	7.5870	7.5870	7.5870	7.5870	7.5870	7.5870
Const. Color	TF138	5.0000	5.0000	5.0000	5.0000	5.0000	5.0000	5.0000	5.0000	5.0000	5.0000	5.0000	5.0000	5.0000	5.0000	5.0000	5.0000
Stuffer	GLM171	55.3322	55.3313	55.3297	55.3263	55.3197	55.3063	55.2797	55.2263	55.1196	54.9063	54.4795	53.6260	51.9190	48.5050	41.6770	28.0210

Open Loop

Reference																Units:	ng
DBD	CH335	32.0800	32.0800	32.0800	32.0800	32.0800	32.0800	32.0800	32.0800	32.0800	32.0800	32.0800	32.0800	32.0800	32.0800	32.0800	32.0800
AD	CH340	0.0008	0.0017	0.0033	0.0067	0.0133	0.0267	0.0533	0.1067	0.2134	0.4268	0.8535	1.7070	3.4140	6.8280	13.6560	27.3120
Reporter	SA506	4.8000	4.8000	4.8000	4.8000	4.8000	4.8000	4.8000	4.8000	4.8000	4.8000	4.8000	4.8000	4.8000	4.8000	4.8000	4.8000
Const. Color	TF138	5.0000	5.0000	5.0000	5.0000	5.0000	5.0000	5.0000	5.0000	5.0000	5.0000	5.0000	5.0000	5.0000	5.0000	5.0000	5.0000
Stuffer	GLM171	58.1192	58.1183	58.1167	58.1133	58.1067	58.0933	58.0667	58.0133	57.9066	57.6933	57.2665	56.4130	54.7060	51.2920	44.4640	30.8080

Disturbance																Units:	ng
DBD	CH335	32.0800	32.0800	32.0800	32.0800	32.0800	32.0800	32.0800	32.0800	32.0800	32.0800	32.0800	32.0800	32.0800	32.0800	32.0800	32.0800
AD	CH340	0.0008	0.0017	0.0033	0.0067	0.0133	0.0267	0.0533	0.1067	0.2134	0.4268	0.8535	1.7070	3.4140	6.8280	13.6560	27.3120
Reporter	SA506	7.2150	7.2150	7.2150	7.2150	7.2150	7.2150	7.2150	7.2150	7.2150	7.2150	7.2150	7.2150	7.2150	7.2150	7.2150	7.2150
Const. Color	TF138	5.0000	5.0000	5.0000	5.0000	5.0000	5.0000	5.0000	5.0000	5.0000	5.0000	5.0000	5.0000	5.0000	5.0000	5.0000	5.0000
Stuffer	GLM171	55.7042	55.7033	55.7017	55.6983	55.6917	55.6783	55.6517	55.5983	55.4916	55.2783	54.8515	53.9980	52.2910	48.8770	42.0490	28.3930

Supplementary Table 25: This transfection table shows all the tested setpoints for the inactive-Int controller presented in Fig. 7. The normalized data plots for the TetR controller depicted in Fig. 7 show only the values in black that correspond to the setpoints exhibiting RPA when operating in closed loop. The greyed out values denote the setpoints that do not exhibit RPA when operating in closed loop.

IntN

		70C	70N	155C	155N	180C	180N	184C	184N	193C	193N	TetR-IntC-3xVP16	tTA
Activator	SA237	12.5	-	-	-	-	-	-	-	-	-	-	-
	SA231	-	12.5	-	-	-	-	-	-	-	-	-	-
	SA247	-	-	12.5	-	-	-	-	-	-	-	-	-
	SA239	-	-	-	12.5	-	-	-	-	-	-	-	-
	SA233	-	-	-	-	12.5	-	-	-	-	-	-	-
	SA232	-	-	-	-	-	12.5	-	-	-	-	-	-
	SA234	-	-	-	-	-	-	12.5	-	-	-	-	-
	SA238	-	-	-	-	-	-	-	12.5	-	-	-	-
	SA235	-	-	-	-	-	-	-	-	12.5	-	-	-
	SA236	-	-	-	-	-	-	-	-	-	12.5	-	-
	SA193	-	-	-	-	-	-	-	-	-	-	12.5	-
	MTK837	-	-	-	-	-	-	-	-	-	-	-	12.5
IntN	SA153	12.5	12.5	12.5	12.5	12.5	12.5	12.5	12.5	12.5	12.5	12.5	12.5
Reporter	MTK833	25	25	25	25	25	25	25	25	25	25	25	25
Stuffer	GLM171	50	50	50	50	50	50	50	50	50	50	50	50

Supplementary Table 26: Transfection Table for Supplementary Fig. 23 with constitutive expression of Int^{N}.

IntC

		70C	70N	155C	155N	180C	180N	184C	184N	193C	193N	TetR-IntC-3xVP16	tTA
Activator	SA237	12.5	-	-	-	-	-	-	-	-	-	-	-
	SA231	-	12.5	-	-	-	-	-	-	-	-	-	-
	SA247	-	-	12.5	-	-	-	-	-	-	-	-	-
	SA239	-	-	-	12.5	-	-	-	-	-	-	-	-
	SA233	-	-	-	-	12.5	-	-	-	-	-	-	-
	SA232	-	-	-	-	-	12.5	-	-	-	-	-	-
	SA234	-	-	-	-	-	-	12.5	-	-	-	-	-
	SA238	-	-	-	-	-	-	-	12.5	-	-	-	-
	SA235	-	-	-	-	-	-	-	-	12.5	-	-	-
	SA236	-	-	-	-	-	-	-	-	-	12.5	-	-
	SA193	-	-	-	-	-	-	-	-	-	-	12.5	-
	MTK837	-	-	-	-	-	-	-	-	-	-	-	12.5
IntC	SA152	12.5	12.5	12.5	12.5	12.5	12.5	12.5	12.5	12.5	12.5	12.5	12.5
Reporter	MTK833	25	25	25	25	25	25	25	25	25	25	25	25
Stuffer	GLM171	50	50	50	50	50	50	50	50	50	50	50	50

Supplementary Table 27: Transfection Table for Supplementary Fig. 23 with constitutive expression of Int^{C}.

ZF

TF with IntC															Units:	ng
Activator	SA172	0.0012	0.0025	0.0050	0.0099	0.0198	0.0397	0.0793	0.1587	0.3173	0.6346	1.2692	2.5385	5.0769	10.1539	20.3078	40.6155
Reporter	SA479	6.6306	6.6306	6.6306	6.6306	6.6306	6.6306	6.6306	6.6306	6.6306	6.6306	6.6306	6.6306	6.6306	6.6306	6.6306	6.6306
Const. Color	TF138	5.0000	5.0000	5.0000	5.0000	5.0000	5.0000	5.0000	5.0000	5.0000	5.0000	5.0000	5.0000	5.0000	5.0000	5.0000	5.0000
Stuffer	GLM171	88.3682	88.3669	88.3644	88.3595	88.3496	88.3297	88.2901	88.2107	88.0521	87.7348	87.1002	85.8309	83.2925	78.2155	68.0617	47.7539

TF															Units:	ng
Activator	SA194	0.0024	0.0048	0.0095	0.0191	0.0381	0.0762	0.1525	0.3049	0.6099	1.2197	2.4395	4.8789	9.7579	19.5158	39.0315	78.0630
Reporter	SA479	6.6306	6.6306	6.6306	6.6306	6.6306	6.6306	6.6306	6.6306	6.6306	6.6306	6.6306	6.6306	6.6306	6.6306	6.6306	6.6306
Const. Color	TF138	5.0000	5.0000	5.0000	5.0000	5.0000	5.0000	5.0000	5.0000	5.0000	5.0000	5.0000	5.0000	5.0000	5.0000	5.0000	5.0000
Stuffer	GLM171	88.3670	88.3646	88.3599	88.3503	88.3313	88.2932	88.2169	88.0645	87.7595	87.1497	85.9299	83.4905	78.6115	68.8537	49.3379	10.3064

IntraDD

TF with IntC															Units:	ng
Activator	SA324	0.0024	0.0049	0.0098	0.0196	0.0392	0.0783	0.1567	0.3134	0.6267	1.2534	2.5069	5.0138	10.0275	20.0550	40.1100	80.2200
Reporter	SA478	6.8418	6.8418	6.8418	6.8418	6.8418	6.8418	6.8418	6.8418	6.8418	6.8418	6.8418	6.8418	6.8418	6.8418	6.8418	6.8418
Const. Color	TF138	5.0000	5.0000	5.0000	5.0000	5.0000	5.0000	5.0000	5.0000	5.0000	5.0000	5.0000	5.0000	5.0000	5.0000	5.0000	5.0000
Stuffer	GLM171	88.1558	88.1533	88.1484	88.1386	88.1190	88.0799	88.0015	87.8448	87.5315	86.9048	85.6513	83.1445	78.1307	68.1032	48.0482	7.9382

TF															Units:	ng
Activator	SA494	0.0024	0.0048	0.0096	0.0192	0.0384	0.0767	0.1534	0.3068	0.6136	1.2273	2.4546	4.9091	9.8183	19.6366	39.2732	78.5463
Reporter	SA478	6.8418	6.8418	6.8418	6.8418	6.8418	6.8418	6.8418	6.8418	6.8418	6.8418	6.8418	6.8418	6.8418	6.8418	6.8418	6.8418
Const. Color	TF138	5.0000	5.0000	5.0000	5.0000	5.0000	5.0000	5.0000	5.0000	5.0000	5.0000	5.0000	5.0000	5.0000	5.0000	5.0000	5.0000
Stuffer	GLM171	88.1558	88.1534	88.1486	88.1390	88.1198	88.0815	88.0048	87.8514	87.5446	86.9309	85.7036	83.2491	78.3399	68.5216	48.8851	9.6119

Supplementary Table 28: Transfection Table for Supplementary Fig. 24 corresponding to the ZF and intraDD controllers.

TetR

TF with IntC																Units:	ng
Activator	SA193	0.0026	0.0052	0.0105	0.0209	0.0418	0.0836	0.1673	0.3345	0.6691	1.3381	2.6763	5.3526	10.7051	21.4103	42.8205	85.6410
Reporter	SA478	6.8418	6.8418	6.8418	6.8418	6.8418	6.8418	6.8418	6.8418	6.8418	6.8418	6.8418	6.8418	6.8418	6.8418	6.8418	6.8418
Const. Color	TF138	5.0000	5.0000	5.0000	5.0000	5.0000	5.0000	5.0000	5.0000	5.0000	5.0000	5.0000	5.0000	5.0000	5.0000	5.0000	5.0000
Stuffer	GLM171	88.1556	88.1530	88.1477	88.1373	88.1164	88.0746	87.9909	87.8237	87.4891	86.8201	85.4819	82.8056	77.4531	66.7480	45.3377	2.5172

TF																Units:	ng
Activator	SA477	0.0026	0.0052	0.0104	0.0208	0.0416	0.0831	0.1663	0.3326	0.6652	1.3303	2.6606	5.3213	10.6425	21.2850	42.5700	85.1400
Reporter	SA478	6.8418	6.8418	6.8418	6.8418	6.8418	6.8418	6.8418	6.8418	6.8418	6.8418	6.8418	6.8418	6.8418	6.8418	6.8418	6.8418
Const. Color	TF138	5.0000	5.0000	5.0000	5.0000	5.0000	5.0000	5.0000	5.0000	5.0000	5.0000	5.0000	5.0000	5.0000	5.0000	5.0000	5.0000
Stuffer	GLM171	88.1556	88.1530	88.1478	88.1374	88.1166	88.0751	87.9919	87.8256	87.4930	86.8279	85.4976	82.8370	77.5157	66.8732	45.5882	3.0182

GAL4

TF with IntC																Units:	ng
Activator	SA286	0.0025	0.0050	0.0100	0.0199	0.0399	0.0797	0.1595	0.3190	0.6379	1.2759	2.5517	5.1034	10.2068	20.4137	40.8274	81.6548
Reporter	SA480	6.7047	6.7047	6.7047	6.7047	6.7047	6.7047	6.7047	6.7047	6.7047	6.7047	6.7047	6.7047	6.7047	6.7047	6.7047	6.7047
Const. Color	TF138	5.0000	5.0000	5.0000	5.0000	5.0000	5.0000	5.0000	5.0000	5.0000	5.0000	5.0000	5.0000	5.0000	5.0000	5.0000	5.0000
Stuffer	GLM171	88.2928	88.2903	88.2853	88.2754	88.2554	88.2156	88.1358	87.9763	87.6574	87.0194	85.7436	83.1919	78.0885	67.8816	47.4679	6.6405

TF																Units:	ng
Activator	SA476	0.0024	0.0049	0.0097	0.0195	0.0390	0.0780	0.1560	0.3120	0.6239	1.2478	2.4957	4.9913	9.9827	19.9654	39.9308	79.8615
Reporter	SA480	6.7047	6.7047	6.7047	6.7047	6.7047	6.7047	6.7047	6.7047	6.7047	6.7047	6.7047	6.7047	6.7047	6.7047	6.7047	6.7047
Const. Color	TF138	5.0000	5.0000	5.0000	5.0000	5.0000	5.0000	5.0000	5.0000	5.0000	5.0000	5.0000	5.0000	5.0000	5.0000	5.0000	5.0000
Stuffer	GLM171	88.2929	88.2904	88.2856	88.2758	88.2563	88.2173	88.1393	87.9833	87.6714	87.0475	85.7996	83.3040	78.3126	68.3299	48.3646	8.4338

Supplementary Table 29: Transfection Table for Supplementary Fig. 24 corresponding to the TetR and Gal4 controllers.

ZF

with intein													Units:	ng
disturbed		no intein	1/256	1/128	1/64	1/32	1/16	1/8	1/4	1/2	1x	2x	4x	reporter only
Activator	SA172	1.2692	1.2692	1.2692	1.2692	1.2692	1.2692	1.2692	1.2692	1.2692	1.2692	1.2692	1.2692	-
Const. IntN	pMTK1138	-	0.0050	0.0099	0.0198	0.0397	0.0793	0.1587	0.3173	0.6346	1.2692	2.5385	5.0769	-
Reporter	SA479	6.6306	6.6306	6.6306	6.6306	6.6306	6.6306	6.6306	6.6306	6.6306	6.6306	6.6306	6.6306	6.6306
Const. Color	TF138	5.0000	5.0000	5.0000	5.0000	5.0000	5.0000	5.0000	5.0000	5.0000	5.0000	5.0000	5.0000	5.0000
Stuffer	GLM171	85.8309	85.8260	85.8210	85.8111	85.7913	85.7516	85.6723	85.5136	85.1963	84.5617	83.2925	80.7540	88.3694

IntraDD

with intein													Units:	ng
disturbed		no intein	1/256	1/128	1/64	1/32	1/16	1/8	1/4	1/2	1x	2x	4x	reporter only
Activator	SA324	10.0275	10.0275	10.0275	10.0275	10.0275	10.0275	10.0275	10.0275	10.0275	10.0275	10.0275	10.0275	-
Const. IntN	pMTK1138	-	0.0392	0.0783	0.1567	0.3134	0.6267	1.2534	2.5069	5.0138	10.0275	20.0550	40.1100	-
Reporter	SA478	6.8418	6.8418	6.8418	6.8418	6.8418	6.8418	6.8418	6.8418	6.8418	6.8418	6.8418	6.8418	6.8418
Const. Color	TF138	5.0000	5.0000	5.0000	5.0000	5.0000	5.0000	5.0000	5.0000	5.0000	5.0000	5.0000	5.0000	5.0000
Stuffer	GLM171	68.1032	68.0640	68.0249	67.9465	67.7898	67.4765	66.8498	65.5963	63.0895	58.0757	48.0482	27.9932	88.1582

Supplementary Table 30: Transfection Table for Supplementary Fig. 25 corresponding to the ZF and intraDD controllers.

TetR

with intein												Units:	ng
disturbed		no intein	1/256	1/128	1/64	1/32	1/16	1/8	1/4	1/2	1x	2x	4x	reporter only
Activator	SA193	10.7051	10.7051	10.7051	10.7051	10.7051	10.7051	10.7051	10.7051	10.7051	10.7051	10.7051	10.7051	-
Const. IntN	pMTK1138	-	0.0418	0.0836	0.1673	0.3345	0.6691	1.3381	2.6763	5.3526	10.7051	21.4103	42.8205	-
Reporter	SA478	6.8418	6.8418	6.8418	6.8418	6.8418	6.8418	6.8418	6.8418	6.8418	6.8418	6.8418	6.8418	6.8418
Const. Color	TF138	5.0000	5.0000	5.0000	5.0000	5.0000	5.0000	5.0000	5.0000	5.0000	5.0000	5.0000	5.0000	5.0000
Stuffer	GLM171	66.7480	66.7061	66.6643	66.5807	66.4134	66.0789	65.4098	64.0717	61.3954	56.0428	45.3377	23.9275	88.1582

Gal4

with intein												Units:	ng
disturbed		no intein	1/256	1/128	1/64	1/32	1/16	1/8	1/4	1/2	1x	2x	4x	reporter only
Activator	SA286	1.2759	1.2759	1.2759	1.2759	1.2759	1.2759	1.2759	1.2759	1.2759	1.2759	1.2759	1.2759	-
Const. IntN	pMTK1138	-	0.0050	0.0100	0.0199	0.0399	0.0797	0.1595	0.3190	0.6379	1.2759	2.5517	5.1034	-
Reporter	SA480	6.7047	6.7047	6.7047	6.7047	6.7047	6.7047	6.7047	6.7047	6.7047	6.7047	6.7047	6.7047	6.7047
Const. Color	TF138	5.0000	5.0000	5.0000	5.0000	5.0000	5.0000	5.0000	5.0000	5.0000	5.0000	5.0000	5.0000	5.0000
Stuffer	GLM171	85.7436	85.7386	85.7336	85.7237	85.7037	85.6638	85.5841	85.4246	85.1057	84.4677	83.1919	80.6402	88.2953

Supplementary Table 31: Transfection Table for Supplementary Fig. 25 corresponding to the TetR and Gal4 controllers.

Closed Loop

Reference													Units:	ng
Activator	SA489	0.0025	0.0050	0.0100	0.0200	0.0399	0.0799	0.1597	0.3195	0.6390	1.2780	2.5559	5.1118	10.2236
Reporter	SA497	4.4222	4.4222	4.4222	4.4222	4.4222	4.4222	4.4222	4.4222	4.4222	4.4222	4.4222	4.4222	4.4222
Const. Color	TF138	5.0000	5.0000	5.0000	5.0000	5.0000	5.0000	5.0000	5.0000	5.0000	5.0000	5.0000	5.0000	5.0000
Stuffer	GLM171	90.5753	90.5728	90.5678	90.5578	90.5378	90.4979	90.4180	90.2583	89.9388	89.2998	88.0219	85.4659	80.3541

Disturbance													Units:	ng
Activator	SA489	0.0025	0.0050	0.0100	0.0200	0.0399	0.0799	0.1597	0.3195	0.6390	1.2780	2.5559	5.1118	10.2236
Reporter	SA497	7.3761	7.3761	7.3761	7.3761	7.3761	7.3761	7.3761	7.3761	7.3761	7.3761	7.3761	7.3761	7.3761
Const. Color	TF138	5.0000	5.0000	5.0000	5.0000	5.0000	5.0000	5.0000	5.0000	5.0000	5.0000	5.0000	5.0000	5.0000
Stuffer	GLM171	87.6214	87.6189	87.6139	87.6039	87.5840	87.5440	87.4642	87.3044	86.9849	86.3459	85.0680	82.5121	77.4003

Open Loop

Reference													Units:	ng
Activator	SA489	0.0025	0.0050	0.0100	0.0200	0.0399	0.0799	0.1597	0.3195	0.6390	1.2780	2.5559	5.1118	10.2236
Reporter	SA263	4.4222	4.4222	4.4222	4.4222	4.4222	4.4222	4.4222	4.4222	4.4222	4.4222	4.4222	4.4222	4.4222
Const. Color	TF138	5.0000	5.0000	5.0000	5.0000	5.0000	5.0000	5.0000	5.0000	5.0000	5.0000	5.0000	5.0000	5.0000
Stuffer	GLM171	90.5753	90.5728	90.5678	90.5578	90.5378	90.4979	90.4180	90.2583	89.9388	89.2998	88.0219	85.4659	80.3541

Disturbance													Units:	ng
Activator	SA489	0.0025	0.0050	0.0100	0.0200	0.0399	0.0799	0.1597	0.3195	0.6390	1.2780	2.5559	5.1118	10.2236
Reporter	SA263	7.3761	7.3761	7.3761	7.3761	7.3761	7.3761	7.3761	7.3761	7.3761	7.3761	7.3761	7.3761	7.3761
Const. Color	TF138	5.0000	5.0000	5.0000	5.0000	5.0000	5.0000	5.0000	5.0000	5.0000	5.0000	5.0000	5.0000	5.0000
Stuffer	GLM171	87.6214	87.6189	87.6139	87.6039	87.5840	87.5440	87.4642	87.3044	86.9849	86.3459	85.0680	82.5121	77.4003

Supplementary Table 32: Transfection Table for Supplementary Fig. 26 corresponding to the open- and closed-loop settings.

References

[1] Patricia Carvajal-Vallejos, Roser Pallissé, Henning D Mootz, and Stefan R Schmidt. Unprecedented rates and efficiencies revealed for new natural split inteins from metagenomic sources. *Journal of Biological Chemistry*, 287(34):28686–28696, 2012.

[2] Maurice Filo, Sant Kumar, and Mustafa Khammash. A hierarchy of biomolecular proportional-integral-derivative feedback controllers for robust perfect adaptation and dynamic performance. *Nature Communications*, 13(1):1–19, 2022.

[3] Hassan K Khalil. Nonlinear systems third edition. *Patience Hall*, 115, 2002.

[4] Martin Feinberg. Chemical reaction network structure and the stability of complex isothermal reactors—i. the deficiency zero and deficiency one theorems. *Chemical engineering science*, 42(10):2229–2268, 1987.

[5] Maurice G Filo, Sant Kumar, Stanislav Anastassov, and Mustafa Khammash. Exploiting the nonlinear structure of the antithetic integral controller to enhance dynamic performance. *bioRxiv*, 2022.

[6] Michael Chevalier, Mariana Gómez-Schiavon, Andrew H Ng, and Hana El-Samad. Design and analysis of a proportional-integral-derivative controller with biological molecules. *Cell Systems*, 9(4):338–353, 2019.

[7] Ronghui Zhu, Jesus M del Rio-Salgado, Jordi Garcia-Ojalvo, and Michael B Elowitz. Synthetic multistability in mammalian cells. *Science*, 375(6578):eabg9765, 2022.

[8] Daniel T Gillespie. Exact stochastic simulation of coupled chemical reactions. *The journal of physical chemistry*, 81(25):2340–2361, 1977.

[9] Euler cluster specifications. https://scicomp.ethz.ch/wiki/Euler, 2022.

[10] Yimeng Zeng, Alicia M Jones, Emily E Thomas, Barbara Nassif, Jonathan J Silberg, and Laura Segatori. A split transcriptional repressor that links protein solubility to an orthogonal genetic circuit. *ACS synthetic biology*, 7(9):2126–2138, 2018.

[11] Gabriele Lillacci, Yaakov Benenson, and Mustafa Khammash. Synthetic control systems for high performance gene expression in mammalian cells. *Nucleic acids research*, 46(18):9855–9863, 2018.

[12] Timothy Frei, Federica Cella, Fabiana Tedeschi, Joaquín Gutiérrez, Guy-Bart Stan, Mustafa Khammash, and Velia Siciliano. Characterization and mitigation of gene expression burden in mammalian cells. *Nature communications*, 11(1):1–14, 2020.
