# Supplementary material for: A cybergenetic framework for engineering intein-mediated integral feedback control systems
Source: Nat Commun. 2023 Mar 11;14:1337. doi: 10.1038/s41467-023-36863-9 (PMC10008564; doi:10.1038/s41467-023-36863-9)
Supplement: Supplementary file 2 — Reporting Summary [file 41467_2023_36863_MOESM2_ESM.pdf]

nature portfolio

Corresponding author(s): Professor Mustafa Khammash

Last updated by author(s): Feb 6, 2023

Reporting Summary

Nature Portfolio wishes to improve the reproducibility of the work that we publish. This form provides structure for consistency and transparency in reporting. For further information on Nature Portfolio policies, see our Editorial Policies and the Editorial Policy Checklist.

Statistics

For all statistical analyses, confirm that the following items are present in the figure legend, table legend, main text, or Methods section.

n/a	Confirmed	
☐	☒	The exact sample size (*n*) for each experimental group/condition, given as a discrete number and unit of measurement
☒	☐	A statement on whether measurements were taken from distinct samples or whether the same sample was measured repeatedly
☒	☐	The statistical test(s) used AND whether they are one- or two-sided<br>*Only common tests should be described solely by name; describe more complex techniques in the Methods section.*
☒	☐	A description of all covariates tested
☒	☐	A description of any assumptions or corrections, such as tests of normality and adjustment for multiple comparisons
☐	☒	A full description of the statistical parameters including central tendency (e.g. means) or other basic estimates (e.g. regression coefficient) AND variation (e.g. standard deviation) or associated estimates of uncertainty (e.g. confidence intervals)
☒	☐	For null hypothesis testing, the test statistic (e.g. *F*, *t*, *r*) with confidence intervals, effect sizes, degrees of freedom and *P* value noted<br>*Give P values as exact values whenever suitable.*
☒	☐	For Bayesian analysis, information on the choice of priors and Markov chain Monte Carlo settings
☒	☐	For hierarchical and complex designs, identification of the appropriate level for tests and full reporting of outcomes
☒	☐	Estimates of effect sizes (e.g. Cohen's *d*, Pearson's *r*), indicating how they were calculated

Our web collection on statistics for biologists contains articles on many of the points above.

Software and code

Policy information about availability of computer code

Data collection	CytExpert 2.3 software
Data analysis	All data analyses in this study was performed on CytExpert 2.3 software exported to Excel (Microsoft Office Professional Plus 2016) and plotted with GraphPad 8.2.0. All simulation analyses in this study were performed on MATLAB R2021a (academic use) platform using custom scripts. Manuscript figures were structured and formatted on Illustrator (2022 26.5), MATLAB and TexStudio (v3.1.1, open source). Stochastic simulations shown in the supplementary information file are carried out on the Euler cluster (https://scicomp.ethz.ch/wiki/Euler). Simulation and manuscript figure generating codes are available here: https://github.com/Maurice-Filo/Inteins-in-the-loop

For manuscripts utilizing custom algorithms or software that are central to the research but not yet described in published literature, software must be made available to editors and reviewers. We strongly encourage code deposition in a community repository (e.g. GitHub). See the Nature Portfolio guidelines for submitting code & software for further information.

Data

Policy information about availability of data

All manuscripts must include a data availability statement. This statement should provide the following information, where applicable:

- Accession codes, unique identifiers, or web links for publicly available datasets
- A description of any restrictions on data availability
- For clinical datasets or third party data, please ensure that the statement adheres to our policy

The fluorescence measurements data of Figures 4 and 7 are provided in the Supplementary Information File under Section S9. Plasmid and oligo sequences can be found in the Source Data file.

Human research participants

Policy information about studies involving human research participants and Sex and Gender in Research.

Reporting on sex and gender	Not Applicable.
Population characteristics	Not Applicable.
Recruitment	Not Applicable.
Ethics oversight	Not Applicable.

Note that full information on the approval of the study protocol must also be provided in the manuscript.

Field-specific reporting

Please select the one below that is the best fit for your research. If you are not sure, read the appropriate sections before making your selection.

- [x] Life sciences
- [ ] Behavioural & social sciences
- [ ] Ecological, evolutionary & environmental sciences

For a reference copy of the document with all sections, see nature.com/documents/nr-reporting-summary-flat.pdf

Life sciences study design

All studies must disclose on these points even when the disclosure is negative.

Sample size	All events within the gating strategy collected with the FACS were considered in the analysis. Each experiment was performed at least three times and is on par with standard experiments reported in the field. Data acquisition is described in the Methods section.
Data exclusions	Fluorescent data that was not clearly above background levels were excluded.
Replication	Each experiment was repeated independently at least twice with similar results.
Randomization	The samples were not randomized since position of samples in multi-well plates and order of the flow cytometric acquisiton is not expected to affect the conclusion. Disturbed and Reference samples were always measured back to back for open and closed loop measurements, respectively. Any covariate will affect both open loop and closed loop equally hence rendering the effect not relevant.
Blinding	Knowledge of a samples identity does not affect the experimental conclusion.

Reporting for specific materials, systems and methods

We require information from authors about some types of materials, experimental systems and methods used in many studies. Here, indicate whether each material, system or method listed is relevant to your study. If you are not sure if a list item applies to your research, read the appropriate section before selecting a response.

Materials & experimental systems

n/a	Involved in the study	
☒	☐	Antibodies
☐	☒	Eukaryotic cell lines
☒	☐	Palaeontology and archaeology
☒	☐	Animals and other organisms
☒	☐	Clinical data
☒	☐	Dual use research of concern

Methods

n/a	Involved in the study	
☒	☐	ChIP-seq
☐	☒	Flow cytometry
☒	☐	MRI-based neuroimaging

Eukaryotic cell lines

Policy information about cell lines and Sex and Gender in Research

Cell line source(s)	HEK293T - ATCC
Authentication	None of the cell lines were authenticated
Mycoplasma contamination	Cell lines were tested for mycoplasma contamination and are negative for mycoplasma
Commonly misidentified lines (See ICLAC register)	HEK293T cells are commonly misidentified as HeLa cells. We use them in our study since they are one of the most prominent cell lines.

Flow Cytometry

Plots

Confirm that:

☒ The axis labels state the marker and fluorochrome used (e.g. CD4-FITC).

☒ The axis scales are clearly visible. Include numbers along axes only for bottom left plot of group (a 'group' is an analysis of identical markers).

☒ All plots are contour plots with outliers or pseudocolor plots.

☒ A numerical value for number of cells or percentage (with statistics) is provided.

Methodology

Sample preparation	The cells were detached approximately 48 hours after transfection on the Eppendorf ThermoMixer C at 25°C at 700 rpm with 53 uL Accutase solution (Sigma-Aldrich) per well for 20 min
Instrument	Beckman Coulter CytoFLEX S flow cytometer
Software	CytExpert 2.3 software
Cell population abundance	About 25-40% of all events were analyzed following the gating described below.
Gating strategy	The gates were placed manually. The first gate P1 excludes debris and dead cells based on their size in the SSC-A and the FSC-A. The gate P2 is applied on the P1 subset and selects only single cells based on the FSC- Width. This population is unsed for the gating of positively transfected cells in the FITC-A channel and the APC-A channel, respectively. An example is provided in the supplements.

☒ Tick this box to confirm that a figure exemplifying the gating strategy is provided in the Supplementary Information.
